# Emerging contemporary monetary policy issues in Africa: An application of wavelet and quantile techniques to climatic shocks on inflation

**DOI:** 10.1371/journal.pone.0319797

**Published:** 2025-05-07

**Authors:** Suleiman O. Mamman, Saralees Nadarajah, Jamilu Iliyasu, Mehboob Ul Hassan

**Affiliations:** 1 Graduate School of Economics and Management, Ural Federal University, Russia; 2 School of Mathematics, University of Manchester, Manchester, United Kingdom; 3 Department of Economics, Ahmadu Bello University, Zaria, Nigeria; 4 College of Business Administration, King Saud University, Riyadh, Kingdom of Saudi Arabia; Bilecik University: Bilecik Seyh Edebali Universitesi, TÜRKIYE

## Abstract

Recently, the inflationary impacts of climate change shocks have emerged among key constraints to price and financial stability. In line with this development, some Central banks are incorporating climate change risks in their surveillance activities. Thus, this study examines the asymmetric inflationary impact of climate change shocks on food and general consumer prices in Algeria, Egypt, Nigeria, and South Africa. The study employs a panel quantile via the moment’s method and a wavelet coherency analysis for monthly from 2000M01 to 2023M12. The empirical results reveal that, first, there is a dynamic interconnectedness between climate change shocks and inflation. Secondly, the results show that climate change shocks have an inflationary impact on food and general consumer prices. However, the magnitude and direction of the impact depend on the prevailing inflationary regime. Finally, the analysis shows that climate change shocks raise inflation uncertainty. Collectively, these findings imply that climate change shocks are key sources of inflationary pressures and uncertainty, posing significant challenges to central banks’ inflation management. One implication of these findings is that central banks in these countries will likely face extreme difficulty stabilising inflation since monetary policy instruments are mainly demand management, and thus may be ineffective in dealing with climate change shocks. In line with the findings, the study recommends that these countries should enhance their inflation surveillance and monetary policy strategies but considering the potential climate change risks.

## 1. Introduction

In recent years, the macroeconomic implications of climate change shocks have started shifting the economic landscape and limiting the efficiency of fiscal and monetary policy actions across both developed and developing countries. These shocks have produced profound social and economic impacts on critical components of human existence, requiring swift and coordinated tailored policy responses. For example, the [1] points out that climate change shocks have resulted in widespread degradation of ecosystem structure, function, resilience, and adaptive capacity, as well as changes in seasonal timing, all of which have significant socioeconomic effects. The increasing frequency of climate change shocks can impede the attainment of long-term economic goals due to the increasing intensity and severity of extreme weather events. For example, despite a global increase in agricultural production over the last 50 years, climate change shocks have begun to impede this growth, particularly in mid- and low-latitude regions of the world [2,3].

In addition, there are increasing concerns among Central Banks that climate change shocks are becoming significant threats to price and financial stability. In response, many Central Banks and Apex financial institutions have begun to incorporate climate change risks into their monitoring activities, recognizing their impact on financial stability [4] and inflation [5]. Conceptually, climate change acts primarily as a negative supply shock, whose occurrence and severity lead to inflation and lower output. Therefore, when identifying the key determinants of inflation, it may be counterproductive for central banks to ignore the effects of climate change shocks on consumer prices and output. Moreover, [6] find that the inflationary impact of climate change shocks on food prices and overall inflation presents a significant threat to global price stability.

Contextually, Africa is home to the most vulnerable populations which faces severe impacts of climate change shock, despite not being the primary contributor to global carbon emissions (See WHO. (2024). Climate change is impacting health in Africa. World Health Organization, https://files.aho.afro.who.int/afahobckpcontainer/production/files/iAHO_Climate_change_in_health_Fact_Sheet-April_2024.pdf). The [1] has reported that critical development sectors in Africa have already faced substantial losses and damages because of climate change. For instance, a report in 2020 points out that over the past 60 years, Africa has experienced a rising warming trend exceeding the global average (see footnote 2). In 2022, Africa was responsible for 79 out of 387 global climate disasters and housed 59.6% of the 110.4 million people affected around the world (See WHO. (2024). Climate change is impacting health in Africa. World Health Organization, https://files.aho.afro.who.int/afahobckpcontainer/production/files/iAHO_Climate_change_in_health_Fact_Sheet-April_2024.pdf). Furthermore, climate change has negatively affected agricultural productivity, which has plummeted by 34% since 1961, more than in any other region [7]. Incidentally, food inflation has jumped to its highest levels in three decades, averaging 30%, partly compounded by Russia-Ukraine crisis (See Songwe, V. (2024). The climate crisis: A generational opportunity for Africa. Chapter 2 of Foresight Africa 2024, The Brookings Institution, https://www.brookings.edu/articles/climate-change-foresight-africa-2024/). Despite the profound inflationary impacts of climate change shock on food as well as general consumer prices, its implications for the conduct of monetary policy in Africa remains understudied.

More so, understanding the potential inflationary impacts of climate change shocks on food and general consumer prices is important for effective monetary policy design aiming to enhance the ability of central banks to achieve price stability. Therefore, to guide monetary policy in response to inflation pressures and upside risks, empirical evidence of the magnitude and nature of the inflationary effects of climate change shocks remains paramount. Hence, this study seeks to examine the asymmetric inflationary impact of climate change shocks on food and general consumer prices in Algeria, Egypt, Nigeria, and South Africa. Three key issues drives the motivation of this study. First, the estimates of the asymmetric response of food and general consumer prices could be used to determine the role of inflationary regime in propagating climate change shocks. Secondly, Algeria, Egypt, Nigeria, and South Africa are highly vulnerable to climate-related risks (See Climate Risk Country Profile: Nigeria. *World Bank Group*. https://climateknowledgeportal.worldbank.org/country-profiles) and have the largest GDP (See WBG. (2022). World Development Indicators database, World Bank, 1 July 2022. World Bank Group (WBG), GDP data source: http://data.worldbank.org/data-catalog/world-development-indicators), making them suitable case studies for examining the inflationary impact of climate change shocks in light of the implications for the economies monetary policy. Furthermore, these countries rely heavily on the natural environment for economic activities (such as agriculture and mineral exploration), making them extremely sensitive to climate-related risks. Thirdly, existing studies suggest that climate-change shocks are among the key drivers of inflationary pressures in food and general consumer prices [6,8–15]. While these studies provide invaluable information for monetary policy formulation, they have understudied the role that the inflation regime can play in amplifying or moderating the inflationary effect of climate-change shocks.

Three key findings emerge from empirical analysis. First, the findings show that there is a significant time-dependent dynamic correlation between climate-change shocks and inflation, with the climate change shocks acting as a leading indicator of food and general consumer price movements. By implication, inflation shows that positive climate change shocks may act as early warning indicators of future inflationary pressures in these areas. This information is important for Central Banks because monetary policy is both forward-looking and retroactive actors. Secondly, this study finds that climate change shocks lead to an upside inflation risks in food and general consumer prices. But the severity and direction of the impact depend on the existing inflationary regime. Therefore, this study provides helpful information to monetary authorities in the countries by showing that climate change shocks are significant sources of inflationary pressure. Thus, contributing evidence that suggests the management of inflation may become more difficult in these countries due to their high susceptibility to such external shocks. This is particularly, critical given that traditional monetary policy instruments are not mostly effective in mitigating supply-side shocks introduced by climate change shocks. It also implies achieving inflation targets may be difficult, which is critical for monetary policy to effectively manage inflation. Furthermore, the evidence of asymmetric response suggests that countries with relatively low food and consumer prices may be able to mitigate the inflationary impact of climate change shocks by keeping inflation low. Finally, this analysis shows that climate change shocks raise inflation uncertainty by increasing the volatility of food and other consumer prices. Hence, it contributes to the growing body of work on the inflationary impacts of climate change, focussing on Algeria, Egypt, Nigeria, and South Africa, Africa’s largest economies.

This study is structured as follows. Section 2 examines related literature on the economic impact of climate change. Section 3 explains the method and data used to estimate the inflationary effect of climate change shocks. Section 4 presents and discusses the empirical results. Section 5 concludes the study.

## 2. Review of related literature

In recent times, the rising global temperature anomalies have been caused and amplified by the occurrence of large pollutant emissions, which have elevated the physical risks of climate change shocks to economic growth and inflation upsurge. In addition, a lot of studies have produced empirical evidence indicating that climate change shocks have increasingly become among the key drivers of inflationary surges in food and general consumer prices [6,10,12–14,16–19] These studies have demonstrated that, within the context of time series and panel data analysis, both international and local prices are significantly impacted by environmental climate change shocks. These shocks are associated with a significant increase in food and consumer price inflation in both developed and developing countries. Consequently, this study categorizes the literature into the inflationary impact of climate change shocks on food prices and general consumer prices. In light of the foregoing, evidence shows that food prices respond positively to climate change shocks as detailed in [20]. In addition, empirical studies have confirmed a causal relationship between climate change shocks and food prices [8,9,11]. These studies provide empirical evidence on the response of food prices to climate change shocks. In addition, recent studies also provide estimates showing that climate change shocks raise food prices. For example, [12] show that climate change shocks significantly increase food prices in Turkey. Similarly, [13] find that positive shocks to climate change raise international food prices and draw important implications for the conduct of monetary policy, particularly, in countries with substantial reliance on the environment. In addition, in Egypt, Nigeria, and South Africa, [10] establish evidence showing that climate change shocks are significant sources of inflationary pressures on food and general consumer prices. Also, [6] find that climate change shocks (higher temperatures) raise food and headline inflation persistently over 12 months in both higher- and lower-income countries. The study also predicted an annual upward pressure on food and headline inflation of 0.92-3.23 and 0.32-1.18 percentage points by 2035. Furthermore, [14] show that rising climate change shocks (temperature anomalies) cause substantial increases in food prices in Nigeria.

The evidence from these studies [6,8–14,21] suggests that climate change shocks through their impact on food prices can threatened food security, amplified poverty and complicates monetary policy process. For example, rising food prices can decrease food accessibility by constraining affordability. There is a well-established literature on the causal impact of food prices on food security [22–26]. In addition, rising food prices erode real purchasing power, raise poverty, and make the poor impoverished. Several studies have suggested that high food prices may increase poverty [23–25,27]. Some studies also show that as food prices rise, household welfare tends to fall [28,29]. However, empirical support for the relative impact of high food prices on poverty, welfare, and food security is mixed [24,30,31]. The net impact is determined by whether households are net buyers of staple foods, or the country is a net importer of staple foods.

Furthermore, some studies have demonstrated that climate change shocks drive an upsurge in general consumer prices [15,16,32,33]. For example, [32] show that disaster-related shocks such as floods and hurricanes have caused inflation in 15 Caribbean countries. However, [11] shows that the nature of climate shock and a component of inflation are the primary determinants of the effects of the inflationary impact. Furthermore, evidence indicates that the price impact of climate change shocks is greater and more persistent in developing than in developed economies [16]. Similarly, [19] observe the occurrence of a marginal increase in inflation during the ‘Great Earthquake’ that occurred in East Japan in 2011. In addition, [15] maintained that climate change shocks are still relevant for price stability. In the study, [15] find that while hot summers have a direct and increasing effect on food price increases, especially in the short term within emerging market economies (EMEs), the effect tends to be irrelevant or inverse across a variety of price indices. Similarly, rising temperatures have a significant negative impact on general activities and economic growth, particularly in hotter and less developed economies [34–36].

In summary, the reviewed literature suggests that climate change can causes an upsurge in food and general consumer price inflation. More specifically, the review highlights the paucity of studies on the interaction of climate change shocks with food and general consumer price inflation. In addition, there is little (if any) evidence or studies examining the asymmetric response of food and general consumer price inflation to climate change shocks in Algeria, Egypt, Nigeria, and South Africa. As a result, this study seeks to address this significant gap in the extant literature. In doing so, this study contributes to the existing literature on the response of inflation to climate change shocks, particularly, in Algeria, Egypt, Nigeria, and South Africa, which are the largest economies in Africa. This is important because the potential nonlinear impact of climate change shocks on prices has not been ruled out by existing studies. [15], for instance, indicate that global warming may have a non-linear impact on prices. Furthermore, [37] shows that the pass-through of shocks to prices is affected by the inflationary environment. Based on Taylor’s Hypothesis, the general inflationary environment influences business pricing behaviour and power, which tends to deteriorate more in low-inflation compared to high-inflation environments [37]. The preceding implies that the response of food and general consumer prices to a climate-induced increase in either costs or yield may be affected by the inflation environment.

## 3. Method and data

### 3.1. Theoretical framework

The hybrid new Keynesian Phillips curve model provides a theoretical framework linking the effect of the supply-side shock on output and costs to inflationary pressures. The Keynesian supply shock explains how an asymmetric economic disruption can exert downward pressure on economic activities while driving up prices [38]. In this light, [15] and [16] show that climate change shocks (high-temperature anomalies) represent a productivity shock that could lead to an increase in prices. Conceptually, climate change shocks such as extreme temperatures, are supply shocks and can affect food and general prices through crop yield or input costs such as energy. For example, evidence shows that extreme temperatures hampered crop yield [8,9,39–41] and raise energy [42], which are inputs for the production of many commodities.

On the other hand, the hybrid New Keynesian Philips Curve (NKPC) forecasts a positive response to such shocks emanating from the supply side [43]. Furthermore, Taylor’s Hypothesis predicts that the magnitude of the inflationary impact of shocks will be determined by the inflation regime [37]. Taylor’s hypothesis implies that dynamic responses to inflation at various shocks are regime-dependent and asymmetric. Thus, food and general consumer prices in Africa may not be insulated due to significant reliance of production on the natural environment and its high vulnerability to the impulses of climate-related shocks. Taylor’s Hypothesis predicts that firms in these countries may adjust prices quickly when there is a high-inflation environment and remain cautious in a low-inflation regime due to lower pricing power. This forms the theoretical basis for the current study which seeks to examine the nonlinear response of food prices to extreme temperatures.

### 3.2. Empirical framework

On the one hand, the hybrid NKPC predicts that the effects of climate change shocks on food and general consumer prices would be inflationary due to their effects on productivity and costs of production. On the other hand, Taylor’s Hypothesis predicts that the magnitude and direction of the inflationary impact of climate change shocks would be dependent on the inflation environment and thus, asymmetric. Thus, this study hypothesizes that climate change shocks are likely to be inflationary on food and general consumer prices depending on the environment in Algeria, Egypt, Nigeria, and South Africa. To test this hypothesis, this study cautiously selects a panel quantile via method of moment (MMQR) model of [44]. This model allows the evaluation of the potential asymmetric response of food and general consumer prices to climate-change shocks. This is because the MMQR method estimates regression quantiles by estimating conditional means while still revealing how the regressors affect the entire conditional distribution [44]. Thus, the quantile regression is further presumed to consider different inflation regimes. Following the empirical setup, it expresses that given data $\left\{{\left(Y_{it}X_{it}^{\prime }\right)}^{\prime }\right\}$ from a panel of n individuals i=1,2,…,n over t periods, t=1,…,T, an estimation of the conditional quantiles $Q_{Y}(\tau |X)$ for a location-scale model could be in the form;


$$Y_{it}=\alpha_{i}+X_{it}^{\prime }\beta +\left(\delta_{i}+Z_{it}^{\prime }\gamma \right)U_{it}.$$ (1)

Where $Y_{it}$ is the dependent variable, Inflation (*IF*) represents food and general consumer price changes, whose random conditional quantiles are conditional to a k-vector of co-variates $X_{it}$. By this, $X_{it}$ is the vector of independent variables which include temperature anomalies (TA) and controlled monetary variables of the exchange rate (EX); and treasury bills rate (TR) with $Pr=\left\{\delta_{i}+Z_{it}^{\prime }\gamma >0\right\}=1$. The parameters ($\alpha_{i},\delta_{i}$), i=1,…..,n, capture the individual fixed effects and Z is a *k-vector* of known differentiable (with probability 1) transformations of the components of X. The sequence {$X_{it}$} is strictly exogenous, *iid* for any fixed *i*, and independent across *i*. $U_{it}$ are *iid* (across *i* and *t*), statistically, independent of $X_{it}$, and normalized to satisfy the moment conditions. Thus, model 1 implies:


$$Q_{Y}\left(\tau \;\;\;|\;\;\;X_{it}\right)=\left(\alpha_{i}+\delta_{i}q\left(\tau \right)\right)+X_{it}^{\prime }\beta +Z_{it}^{\prime }\gamma q\left(\tau \right)$$ (2)

The scalar coefficient is the quantile-τ fixed effect for individual i, or the distributional effect at τ. The distributional effect is not, in general, a location shift, unlike the typically fixed effect. In other words, the distributional effect represents the effect of time-invariant individual characteristics that, like other variables, are allowed to have varying effects on various regions of the conditional distribution of Y [44].

In this study, the exchange rate and treasury bills rate control openness as well as monetary policy respectively. Also, following [15], the study measured climate change with temperature anomalies, which [45] are the most appropriate proxy compared to absolute temperatures. In addition, this study follows the leads of [14] to employ Wavelet coherency and phase analysis within the context of 2D and 3D to examine the interaction of climate change shocks with food and general consumer prices. The choice of the wavelet technique allows this study to understand the relationship of climate change shocks with food and general consumer prices within the context of the time and frequency domain. This is important as allows this study to trace the time-varying nature of the interaction and the years of greater correlations. Finally, this study used monthly data from January 2000 to December 2023 for the top five African countries. However, due to the unavailability of data for the fifth country, Ethiopia, this country was excluded from the analysis. The selection of these countries is based on their status as the largest economies in Africa, measured by GDP size. In addition, they represent diverse climatic conditions with varying degrees of vulnerability to climatic shocks. Thus, this selection offers insights into how different climatic challenges affect inflation and monetary policy responses. While it is understood that a larger sample size typically enhances the robustness of the analysis, this limitation is acknowledged as a constraint of the study. Table 1 contains a detailed analysis and description of the data sources. Given that the study is anchored on the NKPC, it considered the exchange rate as a transmission mechanism of monetary policy. A change in exchange rates can directly impact import prices, which in turn affect general price levels and the inflation rate, especially in import-dependent countries. Thus, a depreciated local currency can lead to higher import prices and increased inflation expectations, which is captured in the NKPC framework. On the other hand, the interest rate, proxied by the treasury bill rate, is a key indicator of the interest rate environment. The changes in this rate influence borrowing costs, consumer spending, and investment. It reflects monetary policy decisions and accounts for policy responses to inflation and other economic conditions such as output [46]. The treasury bill rate is selected because, unlike the monetary policy rate, it is market-determined, reflecting the actual cost of short-term borrowing for the government. Also, it reflects the liquidity conditions in the financial market and the demand for safe, short-term government securities [46], making it sensitive to immediate economic developments and policy expectations.

**Table 1:** Data Sources and Description.

Variable	Description	Source
Headline Inflation (HIF) Food Price Inflation (FIF)	The year-on-year percentage changes on the general consumer price index The year-on-year percentage changes on the food price index	FAOSTAT FAOSTAT
Temperature (TA)	Temperatures’ anomalies	FAOSTAT
Exchange rate (EX)	Bilateral exchange rate	IMF International Financial Statistics.
Treasury bills rate (TR)	3-month treasury bills	IMF International Financial Statistics.

**Source:** Author’s compilation

## 4. Empirical results

### 4.1 Statistical properties of the data

Table 2 presents the descriptive statistics for the data used in the analysis. The table reveals that climate change, proxied by temperature anomalies, clusters around the mean values as indicated by the lower value of the standard deviation (0.97) compared to the mean value (1.12). This demonstrates a relatively fair distribution of temperature changes within and across the countries. Similarly, food price inflation and headline inflation are also fairly distributed, with values clustering around their mean values.

**Table 2:** Descriptive Statistics.

Variable	Sample	Mean	Std. dev.	Min	Max	Observations
Climate Change	Overall	1.12	0.97	-2.80	4.33	N = 1104
	Between		0.26	0.94	1.51	n = 4
	Within		0.94	-3.19	4.28	T = 276
Food Price Inflation	Overall	10.43	9.63	-6.01	73.60	N = 1104
	Between		4.98	5.17	14.70	n = 4
	Within		8.60	-9.08	69.34	T = 276
Headline inflation	Overall	8.63	6.24	-2.15	37.92	N = 1104
	Between		4.32	4.66	13.42	n = 4
	Within		5.00	-1.63	35.41	T = 276
Interest rate	Overall	7.64	5.25	0.03	25.62	N = 1099
	Between		4.49	1.67	12.30	n = 4
	Within		3.53	-1.68	22.79	bar = 274.75
Exchange Rate	Overall	82.06	104.11	3.7	770.31	N = 1101
	Between		97.08	10.06	215.80	n = 4
	Within		61.60	-23.73	636.57	T = 275.25

**Source:** Author’s computation

However, we observed a high inflation rate of 73.60, which is notably high and alarming. For macroeconomic variables such as interest rates and exchange rates, while interest rates cluster around the mean (mean (7.64), standard deviation (5.25)), the exchange rate shows significant vulnerability and deviates from the mean, with its standard deviation (104.11) significantly higher than the mean value (82.06). In addition, a scattered plot is analysis is conducted to show the distribution of the key variables (general inflation, food inflation and climate change) overtime (see Appendix A for Figures A1, A2, and A3 in S1 Appendix)

### 4.2. Climate change shocks and inflation dynamics

#### 4.2.1. Climate change shocks and inflation dynamics in Algeria.

Figure B1 (Panels A & B) (see Appendix B in S1 Appendix) shows the results of the wavelet’s phase plot of climate change shocks with general consumer prices (headline inflation) and food price inflation in Algeria. Panels (A) and (B) demonstrate that the phase angles, which demonstrate the lead-lag relationship between climate change shocks and headline and food price inflation, point in opposite directions across time. This highlights the complexities and variations in the links between climate change shocks and headline and food price inflation in Algeria. Some of the arrows in Panels (A) and (B) point to the left, while others face right. Panel (A) reveals that some arrows face North-East at least 64-period horizons from 2001 to 2022 but face North-West at 28 to 32-period horizons from 2020 to 2023.

The first episode suggests that climate change shocks induce a spike in headline inflation (general consumer price) after at least 32 months (approximately 3 years) (see $Lag=\frac{1}{2}\pi =\frac{1}{2}Cycles=\frac{1}{2}\times 64=32$ for computation). This finding suggests that positive climate change shocks can serve as an early warning signal for potential inflationary pressures within a 32-month horizon in Algeria. In addition, Panel (B) shows evidence that food price inflation correlates with climate change shocks. However, Panel (B) indicates that increases in food prices tend to precede climate change shocks by about 16 to 32 months. One possible explanation for this precedence between rising food price inflation and climate change shocks is that increasing living costs may force households in Algeria to rely heavily on the environment for survival and seek out cheaper alternatives. This growing dependency could worsen existing climate change challenges or introduce new ones, leading to increased climate variability, manifesting as global warming and extreme weather events when food prices rise substantially. Overall, this finding suggests that the relationship between climate change shocks and the dynamics of both headline and food price inflation significantly varies in the short and long term.

In addition, Panel (C) shows the three-dimensional (3D) representation of the wavelet coherency of climate change shocks and headline inflation (general consumer prices) from 2000 to 2023. Panel (A) demonstrates that there is a high degree of integration and interconnection between climate change shocks and overall consumer prices in Algeria. This evidence suggests that there is a strong link between these shocks and total consumer prices. However, the strength of the connection varies in frequency (within the year, i.e., monthly) and duration (i.e., over time, say, short-term or long-term). For example, a significant coherence is observed between 2015 and 2018 and 2021 and 2023, particularly in the 1-36-month range. This shows that periods of positive climate change shocks are closely linked to changes in consumer price behaviour in Algeria. Finally, Panel (D) demonstrates a strong and time-varying link between climate change shocks and food price inflation. It shows sizable co-movement from 2006 to 2023 over the horizon of 1 to about 28 months.

#### 4.2.2. Climate change shocks and inflation dynamics in Egypt.

Figure B2 (Panels A and B) (see Appendix B in S1 Appendix) presents the wavelet phase plot results for the link between climate-change shocks and general consumer prices (headline inflation) as well as food price inflation in Egypt. Panels A and B show that the phase angles, which represent the lead-lag relationship between climate change shocks and both types of inflation, move in different directions over time. This alternating direction reveals that there is a complex and different interaction between climate change shocks and Egyptian inflation rates. For example, some arrows in Panels A and B point to the left, and some to the right. This shows that the link between climate change shocks and the dynamics of headline and food price inflation varies significantly in the short and long run.

In addition, Panel (C) depicts the three-dimensional (3D) wavelet coherence of climate change shocks and headline inflation (general consumer prices) from 2000 to 2023. Panel (A) shows a high degree of integration and interconnectedness between climate change shocks and overall consumer prices in Egypt. This shows a substantial link between these shocks and consumer prices; however, the strength varies with frequency (monthly) and duration. For example, between 2008 and 2020, there is a significant coherence in the 10 to 40-month range, demonstrating that periods of positive climate change shocks are closely related to changes in consumer price behaviour. Finally, Panel (D) shows a strong, time-varying link between climate change shocks and food price inflation, with significant co-movement spanning 20 to 50 months.

#### 4.2.3. Climate change shocks and inflation dynamics in Nigeria.

Figure B3 (Panels A and B) (see Appendix B in S1 Appendix) reports the wavelet phase plot results for climate change shocks, general consumer prices (headline inflation), and food price inflation in Nigeria. Panels A and B demonstrate that the phase angles, which represent the lead-lag relationship between climate change shocks with inflation, swing in opposite directions over time. This alternating swing demonstrates a complicated and distinct interaction between climate change shocks and inflation in Nigeria. For instance, some arrows in Panels A and B point to the left, while others point to the right. Furthermore, both Panels (A) and (B) show the prevalence of rightward movement of the arrows near the horizons of the 6-50 period. This implies that the relationship between climate change shocks and the dynamics of headline and food price inflation varies seriously in the short and long run.

Furthermore, Panel (C) shows the three-dimensional (3D) time-varying wavelet coherence between climate change shocks and headline inflation (general consumer prices) in Nigeria. Panel (A) indicates a significant level of integration and interrelation between climate change shocks and overall consumer prices, especially within the medium horizons of 20 to 40 periods. This indicates a significant correlation between these shocks and consumer prices; nonetheless, the degree of impact shifts with frequency (monthly) and duration (short-term and long-term).

#### 4.2.4 Climate change shocks and inflation dynamics in South Africa.

Figure B4 (Panels A and B) (see Appendix B in S1 Appendix) shows the results of the wavelet phase plot, which examines the correlation between climate change shocks and general consumer prices (headline inflation), as well as food price inflation in South Africa. The phase angles shown in Panels A and B represent the lead-lag relationship between climate change shocks and both types of inflation, demonstrating that these relationships shift with time. This alternate structure represents a complicated and varied connection between climate change shocks and inflation in South Africa. For example, some arrows in Panels A and B point to the left, while others point to the right, demonstrating that the relationship between climate change shocks and the dynamics of headline and food price inflation varies significantly in the short and long run.

In addition, Panel (C) reports the three-dimensional (3D) wavelet coherence of climate change shocks and general consumer prices in South Africa. Panel (A) shows a strong integration and interconnection between climate change shocks and overall consumer prices in South Africa. This suggests a strong correlation between these shocks and consumer prices, while their strength varies with frequency (monthly) and duration. For example, between 2004 and 2012, there is significant coherence in the 30 to 40-month range, indicating that periods of positive climate change shocks are closely linked to changes in consumer price behaviour. Finally, Panel (D) demonstrates a strong, time-varying link between climate change shocks and food price inflation, with significant co-movement spanning 10 to 40 months.

### 4.3. Inflationary effects of climate change

#### 4.3.1. Inflationary impact of climate change shocks on food prices.

Table 3 shows the results of the empirical estimation of Equation (2) for the response of food prices to climate-change shocks across various inflation regimes of Algeria, Egypt, Nigeria, and South Africa. Three major responses of food price inflation to climate change shocks are observed in Table 3. First, Table 3 shows that increases in climate-change shocks (rising global warming) raise food price inflation on average. This evidence is indicated by the positive value (0.602) of the location parameter in Table 3. Secondly, Table 3 quantile estimates demonstrate that the response of food prices to climate-change shocks is asymmetric and varies across inflation regimes. This is because the quantile estimates reveal that climate-change shocks cause significant increases in food prices at a higher inflation regime (Q50 to Q90) and lower prices at a very low regime (Q10 to Q40). This means that climate change shocks could have significant inflationary effects when these countries are in a high-inflation environment but experience disinflation in a lower environment. Thirdly, Table 3 estimates also indicates that the rising occurrences of climate change shocks raise food price volatility in these countries. This evidence is indicated by the positive value (0.759) of the scale parameter in Table 3. This means that climate change shocks are significantly associated with increased uncertainty about food price dynamics. Finally, this study observes evidence of a price puzzle in Table 3. The interest rate hike was found to be associated with an increase in food price inflation in these countries. The strength of the monetary policy transmission channel is one plausible reason that can explain the positive response of food price inflation interest hikes as observed in Table 3. The net effect of interest rate hikes on inflation depends on the relative strength of the costs and liquidity channel of the monetary policy transition. When the cost channel significantly predominates the liquidity channel, the net impact of contraction monetary would be inflationary. This is possible when the second-round effect is fully operational and supply-side shocks such as the climate change shocks are significant. In addition, weak connections between policy instruments with targets, undeveloped financial markets, and high monetisation of the economy are other plausible reasons for the dominance of cost channels over liquidity channels.

**Table 3:** Estimates of Inflationary Effect of Climate Change Shocks on Food Price.

Variables	Location	Scale	Qtile_10	Qtile_25	Qtile_50	Qtile_75	Qtile_90
Climate change	0.602	0.759^**^	-0.449	-0.0284	0.505	1.229^*^	2.030^**^
	(0.401)	(0.348)	(0.297)	(0.245)	(0.365)	(0.644)	(0.991)
Interest rate	1.032^***^	0.481^***^	0.367^***^	0.633^***^	0.971^***^	1.429^***^	1.936^***^
	(0.0997)	(0.0866)	(0.0759)	(0.0586)	(0.0883)	(0.147)	(0.229)
Exchange rate	0.0277^***^	0.00332	0.0231^***^	0.0249^***^	0.0272^***^	0.0304^***^	0.0339^***^
	(0.00372)	(0.00323)	(0.00274)	(0.00228)	(0.00339)	(0.00603)	(0.00927)
Constant	-0.453	0.797	-1.556^**^	-1.114^**^	-0.555	0.205	1.046
	(0.913)	(0.793)	(0.673)	(0.564)	(0.84)	(1.502)	(2.304)

Standard errors in parentheses

*** p < 0.01,

** p < 0.05,

* p < 0.1

**Source:** Author’s computation

**Fig 1:**
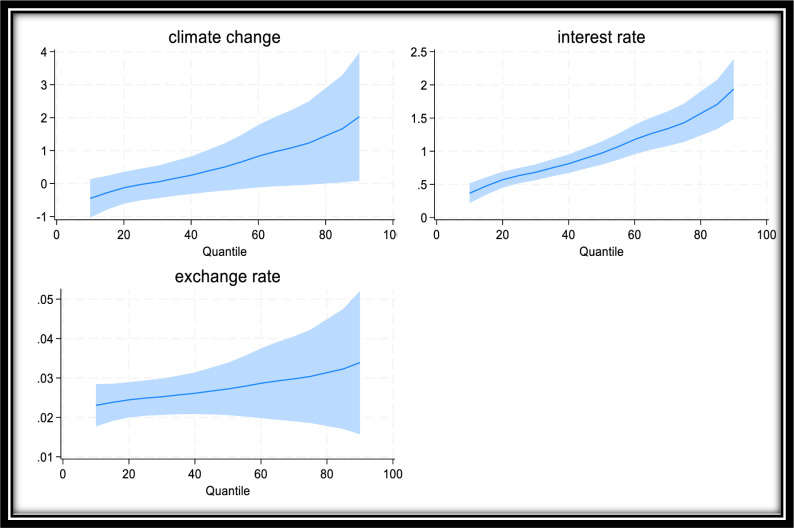
Asymmetric Response of Food Price to Climate Change Shocks.

In addition, Fig 1 presents the asymmetric response of food price inflation to climate change shocks in Algeria, Egypt, Nigeria, and South Africa. Fig 1 shows that climate change shocks have a positive effect on all quantiles except from 40^th^ to 10^th^. Furthermore, Fig 1 reveals that the response of food prices varies significantly across quantiles, with the largest effect observed at the highest inflation (highest quantile) and the smallest effect observed at the lowest prices. Overall, these findings (Table 3 and Fig 1) indicate that climate-change shocks fuel surges in food price inflation, with the magnitude and direction of the impact dependent on the inflation environment. This implies that climate-change shocks tend to have the greatest impact on food prices in high-inflation environments and the least impact in low-inflation environments. This also confirms that the response of food prices to climate change shocks in these countries is nonlinear.

#### 4.3.2. Inflationary impact of climate change on general consumer prices.

Table 4 reports the estimates of the inflationary impact of climate change shocks on general consumer prices derived from the empirical estimation of Equation (2). Table 4 indicates rising climate change shocks raise inflation and price volatility on average. This evidence is indicated by positive coefficient values for the location (0.204) and scale (0.194) parameters reported in Table 4. In addition, Table 4 demonstrates the response of general consumer prices to climate change shocks significantly across quantiles, with the largest effect observed at the 90% quantile (highest inflation regime) and the smallest effect observed at the 10% quantile (lowest inflation regime). This means that rising climate change shocks would likely generate substantial increases in general consumer prices across these countries and the impact is more likely to be large in economies already in a higher inflation environment. Finally, this study observes evidence of a price puzzle in Table 4. The interest rate hike was found to be associated with an increase in food price inflation in these countries. The strength of the monetary policy transmission channel is one plausible reason that can explain the positive response of food price inflation interest hikes as observed in Table 4. The net effect of interest rate hikes on inflation depends on the relative strength of the costs and liquidity channel of the monetary policy transition. When the cost channel significantly predominates the liquidity channel, the net impact of contraction monetary would be inflationary. This is possible when the second-round effect is fully operational and supply-side shocks such as the climate change shocks are significant. In addition, weak connections between policy instruments with targets, undeveloped financial markets, and high monetisation of the economy are other plausible reasons for the dominance of cost channels over liquidity channels.

**Table 4:** Estimates of Inflationary Effect of Climate Change Shocks on General Consumer Prices.

Variables	Location	Scale	Qtile_10	Qtile_25	Qtile_50	Qtile_75	Qtile_90
Climate Change	0.204	0.194^*^	-0.0679	0.0297	0.189	0.351^*^	0.525^*^
	(0.147)	(0.1)	(0.149)	(0.132)	(0.143)	(0.194)	(0.268)
Interest rate	0.737^***^	0.220^***^	0.429^***^	0.540^***^	0.720^***^	0.903^***^	1.100^***^
	(0.0356)	(0.0243)	(0.0352)	(0.0328)	(0.0358)	(0.0456)	(0.0679)
Exchange Rate	0.0282^***^	0.00316^***^	0.0238^***^	0.0254^***^	0.0280^***^	0.0306^***^	0.0334^***^
	(0.00157)	(0.00108)	(0.00159)	(0.00142)	(0.00154)	(0.00207)	(0.00289)
Constant	0.413	1.016^***^	-1.010^***^	-0.499	0.333	1.182^***^	2.092^***^
	(0.34)	(0.232)	(0.349)	(0.306)	(0.334)	(0.454)	(0.622)

Standard errors in parentheses

*** p < 0.01,

** p < 0.05,

* p < 0.1**Source:** Author’s computation

**Fig 2:**
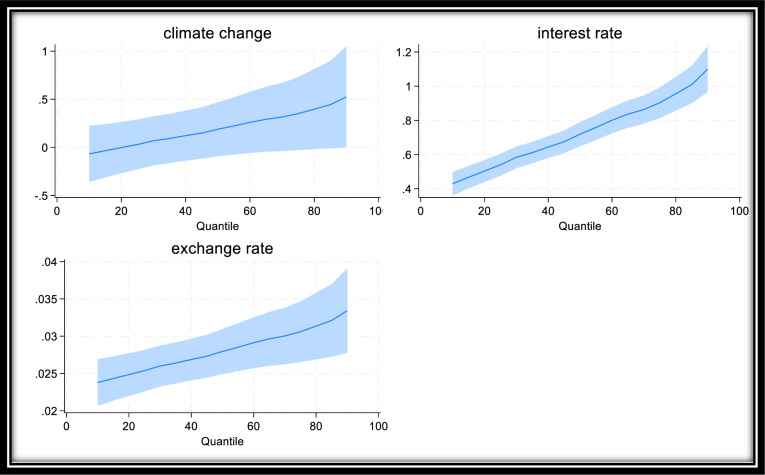
Asymmetric Response of Inflation to Extreme Temperatures. Source: Author’s computation.

Furthermore, Fig 2 presents the asymmetric impact of climate change shocks on the general consumer prices across inflation regimes. From Fig 2, the response of general consumer price climate change shocks varies across regimes, with high regimes experiencing the greatest impact. This result implies that the effect of climate change shock on general consumer price inflation is conditional on the inflation environment, and thus the response is asymmetric. This asymmetric response is confirmed by Table 4.

### 4.4. Sub-sample analysis

A sub-sample analysis was further conducted to determine the effect of varying climatic conditions on the inflation indicators. This analysis used the average climatic anomaly as the benchmark (1.12), with lower values indicating milder climatic conditions and higher values indicating more severe climatic conditions. The analysis was performed for both food inflation (see Tables 5 and 6) and general inflation (see Tables 7 and 8).

**Table 5:** Lower Threshold Estimates of Inflationary Effect of Climate Change Shocks on Food Prices.

Variables	Location	Scale	Qtile_10	Qtile_25	Qtile_50	Qtile_75	Qtile_90
Climate Change	-0.333	-0.0729	-0.224	-0.272	-0.326	-0.394	-0.447
	(0.517)	(0.358)	(0.549)	(0.465)	(0.503)	(0.705)	(0.921)
Interest rate	0.903^***^	0.393^***^	0.317^***^	0.576^***^	0.867^***^	1.231^***^	1.522^***^
	(0.0836)	(0.0579)	(0.0906)	(0.0741)	(0.0827)	(0.112)	(0.149)
Exchange Rate	0.0274^***^	0.00434^*^	0.0209^***^	0.0238^***^	0.0270^***^	0.0310^***^	0.0342^***^
	(0.00351)	(0.00243)	(0.00373)	(0.00315)	(0.00342)	(0.00477)	(0.00625)
Constant	1.132^*^	1.631^***^	-1.294^*^	-0.221	0.984	2.494^***^	3.698^***^
	(0.675)	(0.467)	(0.723)	(0.612)	(0.663)	(0.930)	(1.213)

Standard errors in parentheses

*** p < 0.01,

** p < 0.05,

* p < 0.1

**Table 6:** Upper Threshold Estimates of Inflationary Effect of Climate Change Shocks on Food Prices.

Variables	Location	Scale	Qtile_10	Qtile_25	Qtile_50	Qtile_75	Qtile_90
Climate Change	2.091^**^	1.640^**^	-0.0626	0.735	1.857^**^	3.735^***^	5.379^***^
	(0.861)	(0.640)	(0.690)	(0.624)	(0.806)	(1.377)	(1.959)
Interest rate	1.151^***^	0.551^***^	0.428^***^	0.696^***^	1.073^***^	1.704^***^	2.256^***^
	(0.128)	(0.0951)	(0.110)	(0.0879)	(0.123)	(0.203)	(0.283)
Exchange Rate	0.0302^***^	0.00312	0.0261^***^	0.0276^***^	0.0298^***^	0.0333^***^	0.0365^***^
	(0.00416)	(0.00309)	(0.00329)	(0.00305)	(0.00387)	(0.00667)	(0.00952)
Constant	-4.444^**^	-1.259	-2.791^*^	-3.403^**^	-4.265^**^	-5.706^*^	-6.968
	(2.011)	(1.494)	(1.590)	(1.469)	(1.872)	(3.217)	(4.591)

Standard errors in parentheses

*** p < 0.01,

** p < 0.05,

* p < 0.1

**Table 7:** Lower Threshold Estimates of Inflationary Effect of Climate Change Shocks on General Consumer Prices.

Variables	Location	Scale	Qtile_10	Qtile_25	Qtile_50	Qtile_75	Qtile_90
Climate Change	-0.148	-0.163	0.0903	-0.00285	-0.138	-0.263	-0.388
	(0.301)	(0.211)	(0.328)	(0.280)	(0.296)	(0.388)	(0.518)
Interest rate	0.702^***^	0.186^***^	0.429^***^	0.536^***^	0.691^***^	0.833^***^	0.977^***^
	(0.0461)	(0.0324)	(0.0484)	(0.0437)	(0.0458)	(0.0575)	(0.0821)
Exchange Rate	0.0288^***^	0.00395^**^	0.0230^***^	0.0253^***^	0.0286^***^	0.0316^***^	0.0346^***^
	(0.00221)	(0.00155)	(0.00239)	(0.00206)	(0.00218)	(0.00283)	(0.00382)
Constant	0.901^**^	1.268^***^	-0.960^**^	-0.233	0.826^**^	1.797^***^	2.776^***^
	(0.392)	(0.275)	(0.434)	(0.367)	(0.391)	(0.513)	(0.682)

Standard errors in parentheses

*** p < 0.01,

** p < 0.05, *  p < 0.1

**Table 8:** Upper Threshold Estimates of Inflationary Effect of Climate Change Shocks on General Consumer Prices.

Variables	Location	Scale	Qtile_10	Qtile_25	Qtile_50	Qtile_75	Qtile_90
Climate change	0.934^***^	0.298	0.536^*^	0.671^**^	0.902^***^	1.186^**^	1.477^**^
	(0.339)	(0.222)	(0.324)	(0.297)	(0.329)	(0.461)	(0.642)
Interest rate	0.771^***^	0.244^***^	0.445^***^	0.556^***^	0.745^***^	0.977^***^	1.215^***^
	(0.0527)	(0.0345)	(0.0529)	(0.0464)	(0.0540)	(0.0719)	(0.0998)
Exchange rate	0.0287^***^	0.00249^*^	0.0254^***^	0.0265^***^	0.0285^***^	0.0308^***^	0.0333^***^
	(0.00210)	(0.00138)	(0.00201)	(0.00185)	(0.00205)	(0.00286)	(0.00399)
Constant	-1.342	0.803	-2.414^***^	-2.051^***^	-1.426^*^	-0.661	0.125
	(0.823)	(0.538)	(0.785)	(0.723)	(0.797)	(1.120)	(1.562)

Standard errors in parentheses

*** p < 0.01,

** p < 0.05,

* p < 0.1

Table 5 shows that climatic shocks below the threshold levels (lower anomalies) have a negative and statistically insignificant impact on inflation across all regimes (as indicated by the quantiles). On the other hand, Table 6 reveals that higher climatic anomalies tend to increase food inflation in these countries.

Similarly, the estimates from Tables 7 and 8 for general inflation mirror the outcomes of Tables 5 and 6, respectively. These findings suggest two key points: first, there is an asymmetric impact of climatic shocks on both food and general inflation in these countries. Secondly, lower climatic shocks are associated with an insignificant effect on both inflation indicators, while higher climatic conditions are associated with a positive and significant effect on both inflation indicators, irrespective of the regimes.

### 4.5. Robustness checks of estimate

This study tests the robustness of the estimates in the case of a potential endogeneity bias by estimating Equation (2) using smoothed GMM with quantiles technique (see Table 9 for food price estimate and Table 10 for general price estimates. Consistent with the estimates presented in Table 3, Table 9 shows that there is an asymmetric response of food prices to climate change shocks. More specifically, the occurrence of positive climate change shocks causes an increase in food prices under high inflation environment but lowers it in a lower environment. This is because the estimates in Table 9 indicate that the effect of climate change shock is negative and statistically insignificant at Q10, Q25 and Q50 while it was found to be positive and statistically significant at Q75 and Q90 quantiles. This finding affirms the earlier result that the inflationary impact of climate change on food prices is asymmetric across inflation regimes. This result further suggests that the estimates are robust to alternative estimation techniques that account for the endogeneity problem. Thus, estimates can be reliably used as inputs in decision and policymaking.

**Table 10:** Quantile GMM Estimates of Inflationary Effect of Climate Change Shocks on General Prices.

Variable	Q10	Q25	Q50	Q75	Q90
					
Climate change shocks	1.018	0.393	0.35	1.044	2.821^*^
	(0.702)	(0.721)	(1.668)	(1.093)	(1.659)
Interest rate	0.510^***^	0.461^***^	0.561^***^	0.780^***^	1.078^***^
	(0.0523)	(0.0515)	(0.081)	(0.0541)	(0.0799)
Exchange rate	0.0253^***^	0.0243^***^	0.0282^***^	0.0318^***^	0.0317^***^
	(0.00189)	(0.00251)	(0.00197)	(0.00145)	(0.00408)
Constant	-3.191^**^	-0.257	1.128	1.097	0.818
	(1.398)	(1.112)	(2.232)	(1.325)	(0.679)
Observations	1,088	1,088	1,088	1,088	1,088

Robust standard errors in parentheses

*** p < 0.01,

** p < 0.05,

* p < 0.1

**Source:** Author’s computation

**Table 9:** Quantile GMM Estimates of Inflationary Effect of Climate Change Shocks on Food Price.

Variable	Q10	Q25	Q50	Q75	Q90
Climate change shocks	-0.98	-0.0112	-0.506	1.599^*^	1.875^***^
	(1.389)	(0.643)	(0.732)	(0.852)	(0.725)
Interest rate	0.473^***^	0.542^***^	0.613^***^	1.093^***^	1.533^***^
	(0.0955)	(0.0564)	(0.057)	(0.101)	(0.096)
Exchange rate	0.0347^***^	0.0290^***^	0.0303^***^	0.0324^***^	0.0332^***^
	(0.00405)	(0.00290	(0.00178)	(0.0022)	(0.00299)
Constant	-2.644	-0.821	2.674^***^	1.89	3.147^***^
	(1.677)	(0.927)	(0.976)	(1.233)	(1.015)

Robust standard errors in parentheses

*** p < 0.01,

** p < 0.05,

* p < 0.1

**Source:** Author’s computation.

In addition, Table 10 is also consistent with the estimates presented in Table 4 which shows that there is an asymmetric response of general consumer price to climate change shocks. More specifically, the occurrence of positive climate change shocks causes an increase in general consumer prices but the impact increases with the inflation environment. This is because the estimates in Table 10 indicate that the effect of climate-change shock is lower at Q10, Q25 and Q50 but was found to be positively larger at Q75 and Q90 quantiles. This finding confirms the earlier result that the inflationary impact of climate change on general prices is asymmetric across inflation regimes. This result suggests that the estimates are robust to alternative estimation techniques that account for the endogeneity bias. Thus, estimates can be reliably used as inputs in decision and policymaking.

**Fig 3:**
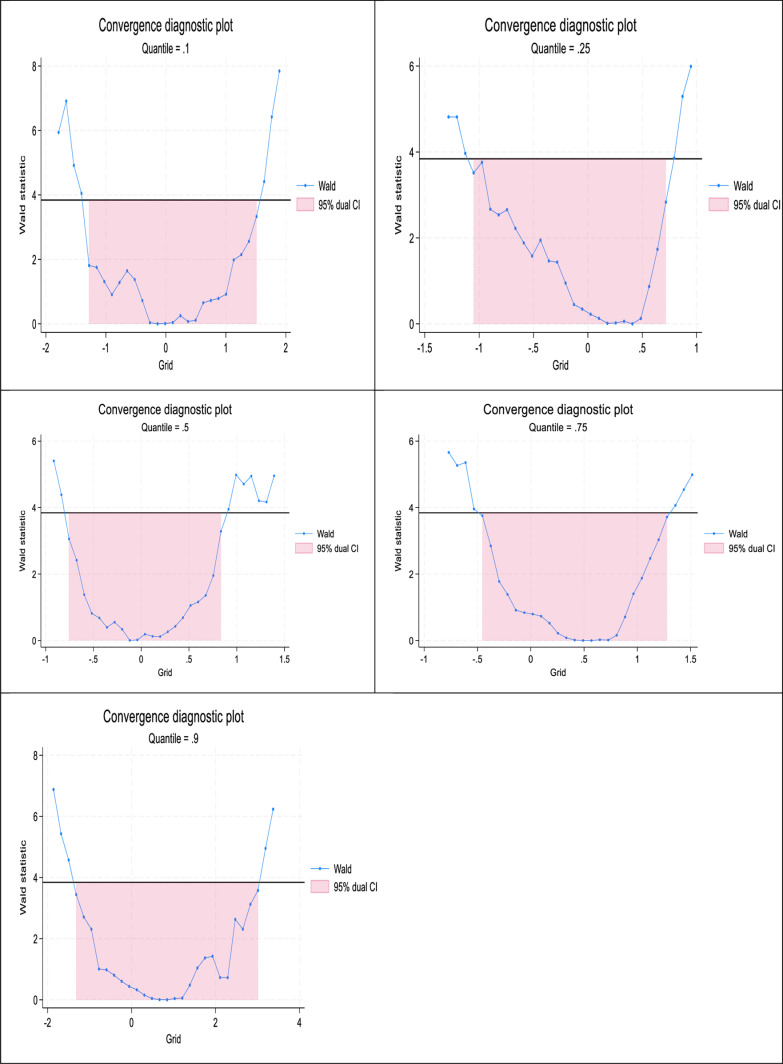
Convergence Plot. **Source:** Author’s computation.

As a diagnostic test, a convergence test was carried using the visual convergence plots under the GMM panel quantile technique (see Fig 3). The convergence test was carried for the 10^th^, 25^th^, 50^th^, 75^th^, and 90^th^ quantiles for Wald statistics using 95% dual confidence interval. The visual illustration reveals a convergence in all the quantile even though the Wald line is not smoothed.

### 4.6 Discussion and Implication of Empirical Results for Monetary Policy

Three key findings emerge from empirical exercise of this study on the interaction of climate-change shocks with general consumer price and food price inflation in Algeria, Egypt, Nigeria, and South Africa. First, the evidence from the wavelet coherency and phase plot demonstrates that climate change shocks drive movements in food and general consumer prices but the strength of the relationship is time-varying. Secondly, the findings show that rising (positive) climate-change shock significantly increases food price inflation in these countries. This finding is consistent with previous studies that found a link between climate-change shocks and an upsurge in food price inflation [6,12–14]. Third, this study finds that positive climate change shocks cause inflation surges in Algeria, Egypt, Nigeria, and South Africa. This finding is consistent with [10,15,16,32] studies, all of which show that climate change shocks trigger increases in the general price level. Finally, this study also observes that the effect of climate change shock on food and general consumer prices is asymmetric indicating that the relationship is nonlinear. It also triggers inflation uncertainty in food and general consumer prices by raising their respective volatilities.

Theoretically, climate change shocks are supply shocks that can influence food prices through their impact on crop yield or input costs such as energy and fertiliser prices. For example, studies have established that rising temperature anomalies reduced crop yield [8,9,39–41] increased both energy and fertiliser prices [42], which are inputs for agricultural commodity production. In addition, the theory of aggregate supply and demand predicts that climate change shocks would cause inflation by acting as a negative shock that reduces output and raises prices. Furthermore, the evidence that the size and direction of food and general consumer price inflation responses to positive climate shock are contingent on the inflation environment is plausible based on Taylor’s Hypothesis. Taylor’s Hypothesis shows that the inflationary environment has an impact on firms’ pricing behaviour and power [37] and thus, adjusts prices faster in high-inflationary regimes than in low-inflationary regimes due to greater pricing power. Therefore, the evidence found here of asymmetric response can be attributed to firms’ low pricing power.

The evidence found in this study of the inflationary impact of climate change shocks on food and general consumer prices has implications for the conduct of monetary policy in these countries. First, the evidence implies that climate change shocks are important sources of inflationary pressures. This implies that both food and general consumer price inflation are highly vulnerable to supply shocks emanating from climate change. This impact can complicate inflation management in these countries because traditional monetary policy tools have limited influence on supply-side shocks originating from climate change. This suggests achieving a target would be difficult, which is required for monetary policy to effectively manage inflation. It also implies climate change shocks may impede price stability in these countries. However, the evidence also implies that policies aimed at moderating climate change through decarbonisation and clean technology may contribute to achieving price stability in these countries.

In addition, the evidence that the inflationary effects of climate change shocks tend to be larger when food and general consumer prices are in a high-inflation environment has implications for the conduct of monetary policy in these countries. One of the implications is that countries that keep food prices and general consumer prices relatively low can mitigate the inflationary impact of climate change shocks on inflation. Thus, achieving low and stable inflation provides immunity against the inflationary impact of climate change shocks. This is because the evidence confirms that the response of food prices and general consumer prices to climate change shocks in these countries is nonlinear and that a country with low inflation may be less affected. However, the result also implies that a country with relatively high inflation may be more vulnerable to the price effects of climate change shock than a country with low inflation. Furthermore, the evidence suggesting that climate change shocks raise inflation uncertainty has implications for inflation management and anchoring of expectations by the monetary policy. Evidence suggests that rising inflation uncertainty makes anchoring inflation expectations difficult [47,48], which is required for monetary policy to effectively manage inflation in these countries. More so, expectations de-anchoring complicate monetary policy and pose a serious challenge to achieving inflation targets.

## 5. Conclusion

This study examines the asymmetric response of food and general price inflation to climate change shocks in Algeria, Egypt, Nigeria, and South Africa. This study employs [44] panel quantile via moments and wavelet techniques estimated with monthly data from 2000M01 to 2020M12. First, the estimates from the wavelet coherency and scalogram show that climate change shocks are an important influence on the inflation process in these countries. Secondly, the estimate from panel quantile via moments indicates that positive climate change shocks have inflationary effects on food and general consumer price, but the size and direction of impact depends on the inflation regime. Finally, the study observes that climate change shocks raise inflation uncertainty by causing elevated increases in food and general price volatility. In general, this study’s analysis suggests that climate change shocks are important sources of inflationary pressures and uncertainty. Thus, represents significant constraints to inflation management by the central banks. One major policy implication of this study’s findings is that central banks in these countries will likely face extreme difficulty stabilising inflation. This is plausible because traditional monetary policy instruments are major demand management tools and thus, would have limited influence on supply-side shocks originating from climate change. Therefore, this study concludes that climate change shocks are important sources of inflation in these countries. Thus, this study recommends that inflation surveillance and monetary policy strategies of these countries consider climate change risks. While this study focuses on macroeconomic analysis using country-level aggregates, future research could explore a micro-level analysis. Specifically, it could investigate how climatic shocks impact individual household consumption patterns due to these shocks.

## Supporting information

S1 Data (XLSX)

S1 Appendix (DOCX)
