# Supplementary material for: Emerging contemporary monetary policy issues in Africa: An application of wavelet and quantile techniques to climatic shocks on inflation
Source: PLoS One. 2025 May 7;20(5):e0319797. doi: 10.1371/journal.pone.0319797 (PMC12057891; doi:10.1371/journal.pone.0319797)
Supplement: S1 Appendix — (DOCX) [file pone.0319797.s002.docx]

**Appendix A**

**Fig A1.** **Scattered plot of food inflation distribution**.

**Source:** Author’s computation

**Fig A2. Scattered plot of general consumer inflation distribution.**

**Source:** Author’s computation

**Fig A3. Scattered plot of temperature anomalies distribution.**

**Source:** Author’s computation

**Appendix B.**

**Fig B1*:* Phase and Coherency Plot of Climate Shocks and Inflation in Algeria.**

**Source:** Author’s computation

**Fig B2: Phase and Coherency Plot of Climate Shocks and Inflation in Egypt.**

**Source:** Author’s computation

**Fig B3: Phase and Coherency Plot of Climate Shocks and Inflation in Nigeria.**

**Source:** Author’s computation

**Fig B4: Phase and Coherency Plot of Climate Shocks and Inflation in South Africa.**

**Source:** Author’s computation

**Data**

| **country** | **period** | **climate** | **cpi_food** | **cpi_head** | **food_inflation** | **TBR** | **exch** | **head_inflation** |
| --- | --- | --- | --- | --- | --- | --- | --- | --- |
| Algeria | Jan-01 | 0.8 | 52.3 | 57.0 | -1.2 | 6 | 74.8127 | -1.0417 |
| Algeria | Feb-01 | -0.5 | 51.6 | 56.3 | -0.3 | 5.96 | 75.745 | -0.1773 |
| Algeria | Mar-01 | 4.3 | 52.2 | 56.9 | 0.9 | 5.94 | 76.4536 | 0.8865 |
| Algeria | Apr-01 | 1.4 | 52.0 | 56.7 | 2.4 | 5.93 | 77.3773 | 2.3466 |
| Algeria | May-01 | 1.3 | 53.4 | 58.1 | 5.6 | 5.93 | 78.3376 | 5.2536 |
| Algeria | Jun-01 | 2.3 | 53.3 | 58.0 | 7.0 | 5.9 | 79.17155 | 6.6176 |
| Algeria | Jul-01 | 2.3 | 52.4 | 57.1 | 6.8 | 5.86 | 79.3581 | 6.3315 |
| Algeria | Aug-01 | 1.6 | 52.7 | 57.4 | 5.7 | 5.8 | 76.9608 | 5.3211 |
| Algeria | Sep-01 | 2.4 | 53.0 | 57.7 | 5.7 | 5.77 | 76.4547 | 5.2920 |
| Algeria | Oct-01 | 3.7 | 53.3 | 58.0 | 6.3 | 5.75 | 76.7019 | 5.8394 |
| Algeria | Nov-01 | 1.3 | 54.9 | 59.6 | 7.8 | 5.69 | 77.6944 | 7.3874 |
| Algeria | Dec-01 | 0.9 | 55.8 | 60.5 | 8.1 | 5.69 | 77.5126 | 7.4600 |
| Algeria | Jan-02 | -0.2 | 53.9 | 60.5 | 3.1 | 4.75 | 77.8893 | 6.1404 |
| Algeria | Feb-02 | 0.6 | 54.3 | 59.8 | 5.3 | 4.5 | 78.9218 | 6.2167 |
| Algeria | Mar-02 | 2.0 | 54.0 | 59.4 | 3.3 | 3.8 | 79.7055 | 4.3937 |
| Algeria | Apr-02 | 1.1 | 53.2 | 58.4 | 2.3 | 2.6 | 80.1848 | 2.9982 |
| Algeria | May-02 | 1.9 | 52.2 | 58.3 | -2.2 | 2.75 | 80.0854 | 0.3442 |
| Algeria | Jun-02 | 1.5 | 51.3 | 57.5 | -3.8 | 2.65 | 79.9995 | -0.8621 |
| Algeria | Jul-02 | 2.0 | 52.2 | 57.2 | -0.4 | 2.6 | 79.9152 | 0.1751 |
| Algeria | Aug-02 | 1.5 | 52.3 | 57.4 | -0.8 | 2.45 | 79.9783 | 0.0000 |
| Algeria | Sep-02 | 1.6 | 52.5 | 57.9 | -1.0 | 2.3 | 79.9378 | 0.3466 |
| Algeria | Oct-02 | 1.3 | 52.8 | 57.8 | -1.1 | 2.15 | 79.9214 | -0.3448 |
| Algeria | Nov-02 | 1.2 | 53.5 | 59.2 | -2.5 | 2 | 79.8512 | -0.6711 |
| Algeria | Dec-02 | 2.3 | 53.1 | 59.6 | -4.8 | 1.8 | 79.7926 | -1.4876 |
| Algeria | Jan-03 | 0.5 | 52.3 | 59.2 | -3.0 | 1.69 | 79.6764 | -2.1488 |
| Algeria | Feb-03 | -0.6 | 53.6 | 59.4 | -1.3 | 1.58 | 79.6406 | -0.6689 |
| Algeria | Mar-03 | 1.2 | 53.1 | 58.8 | -1.7 | 1.5 | 79.6639 | -1.0101 |
| Algeria | Apr-03 | 1.8 | 53.6 | 59.0 | 0.8 | 2.8 | 79.6602 | 1.0274 |
| Algeria | May-03 | 2.1 | 54.4 | 60.1 | 4.1 | 1.75 | 79.4637 | 3.0875 |
| Algeria | Jun-03 | 1.9 | 55.2 | 60.2 | 7.7 | 2 | 79.2982 | 4.6957 |
| Algeria | Jul-03 | 2.5 | 55.0 | 58.8 | 5.3 | 1.39 | 77.8519 | 2.7972 |
| Algeria | Aug-03 | 1.3 | 55.6 | 59.7 | 6.4 | 1.69 | 77.7978 | 4.0070 |
| Algeria | Sep-03 | 1.6 | 55.9 | 60.7 | 6.4 | 1.56 | 77.6679 | 4.8359 |
| Algeria | Oct-03 | 3.5 | 56.6 | 61.0 | 7.4 | 1.5 | 76.3751 | 5.5363 |
| Algeria | Nov-03 | 1.5 | 56.9 | 62.3 | 6.4 | 1.35 | 71.3816 | 5.2365 |
| Algeria | Dec-03 | 0.5 | 55.9 | 62.0 | 5.1 | 1.25 | 70.2624 | 4.0268 |
| Algeria | Jan-04 | 0.8 | 56.6 | 62.9 | 8.2 | 1.18 | 71.6529 | 6.2500 |
| Algeria | Feb-04 | 1.6 | 56.4 | 61.8 | 5.2 | 1.05 | 71.4342 | 4.0404 |
| Algeria | Mar-04 | 1.7 | 57.9 | 62.6 | 9.0 | 1.02 | 71.4562 | 6.4626 |
| Algeria | Apr-04 | 1.0 | 58.1 | 62.2 | 8.3 | 0.95 | 71.5072 | 5.4237 |
| Algeria | May-04 | -0.7 | 57.5 | 62.4 | 5.8 | 0.8 | 71.5148 | 3.8270 |
| Algeria | Jun-04 | 1.0 | 56.7 | 61.5 | 2.8 | 0.55 | 71.459 | 2.1595 |
| Algeria | Jul-04 | 0.7 | 56.9 | 60.6 | 3.4 | 0.38 | 71.7863 | 3.0612 |
| Algeria | Aug-04 | 1.8 | 56.4 | 60.7 | 1.4 | 0.22 | 72.6163 | 1.6750 |
| Algeria | Sep-04 | 0.5 | 58.2 | 62.8 | 4.1 | 2.06 | 72.5972 | 3.4596 |
| Algeria | Oct-04 | 3.2 | 58.6 | 62.8 | 3.4 | 1.9 | 72.9911 | 2.9508 |
| Algeria | Nov-04 | 0.0 | 57.5 | 63.3 | 1.0 | 0.15 | 73.0315 | 1.6051 |
| Algeria | Dec-04 | 0.8 | 56.7 | 63.2 | 1.5 | 0.15 | 72.6811 | 1.9355 |
| Algeria | Jan-05 | -1.7 | 58.4 | 64.8 | 3.2 | 1.1 | 72.6676 | 3.0207 |
| Algeria | Feb-05 | -1.7 | 58.1 | 65.1 | 2.9 | 0.6 | 72.776 | 5.3398 |
| Algeria | Mar-05 | 2.3 | 58.7 | 65.4 | 1.5 | 0.5 | 72.4317 | 4.4728 |
| Algeria | Apr-05 | 2.4 | 59.1 | 65.6 | 1.9 | 0.69 | 72.9271 | 5.4662 |
| Algeria | May-05 | 3.4 | 57.3 | 64.7 | -0.4 | 0.6 | 73.4457 | 3.6859 |
| Algeria | Jun-05 | 1.1 | 56.5 | 64.5 | -0.4 | 0.6 | 73.9264 | 4.8780 |
| Algeria | Jul-05 | 2.3 | 55.1 | 64.1 | -3.1 | 0.9 | 73.1903 | 5.7756 |
| Algeria | Aug-05 | 1.3 | 54.9 | 63.9 | -2.7 | 0.55 | 73.6652 | 5.2718 |
| Algeria | Sep-05 | 0.9 | 54.7 | 63.8 | -6.0 | 0.48 | 73.2956 | 1.5924 |
| Algeria | Oct-05 | 2.7 | 55.3 | 64.1 | -5.6 | 0.5 | 73.4165 | 2.0701 |
| Algeria | Nov-05 | 1.7 | 55.5 | 64.1 | -3.4 | 1.2 | 74.0588 | 1.2638 |
| Algeria | Dec-05 | 0.3 | 56.4 | 64.6 | -0.6 | 1.3 | 73.5148 | 2.2152 |
| Algeria | Jan-06 | -1.1 | 57.6 | 65.2 | -1.4 | 2.23 | 73.0752 | 0.6173 |
| Algeria | Feb-06 | -0.3 | 57.1 | 65.0 | -1.7 | 2.24 | 73.2182 | -0.1536 |
| Algeria | Mar-06 | 2.3 | 58.5 | 65.6 | -0.3 | 2.25 | 74.0224 | 0.3058 |
| Algeria | Apr-06 | 3.9 | 60.0 | 66.3 | 1.4 | 2.24 | 73.127 | 1.0671 |
| Algeria | May-06 | 2.9 | 59.7 | 66.1 | 4.1 | 2.3 | 72.163 | 2.1638 |
| Algeria | Jun-06 | 1.5 | 59.0 | 65.9 | 4.3 | 2.3 | 73.3709 | 2.1705 |
| Algeria | Jul-06 | 1.6 | 57.7 | 65.4 | 4.7 | 2.3 | 73.3469 | 2.0281 |
| Algeria | Aug-06 | 1.7 | 58.5 | 66.0 | 6.6 |  | 72.3466 | 3.2864 |
| Algeria | Sep-06 | 0.3 | 59.6 | 66.5 | 8.9 | 2.1 | 72.0547 | 4.2320 |
| Algeria | Oct-06 | 2.7 | 60.8 | 67.1 | 9.9 | 2.04 | 72.2519 | 4.6802 |
| Algeria | Nov-06 | 1.4 | 59.8 | 66.7 | 7.7 | 1.87 | 71.711 | 4.0562 |
| Algeria | Dec-06 | 0.4 | 60.5 | 67.1 | 7.4 | 1.65 | 71.0716 | 3.8700 |
| Algeria | Jan-07 | 1.2 | 59.7 | 66.7 | 3.7 | 2.2 | 71.4982 | 2.3006 |
| Algeria | Feb-07 | 2.0 | 60.2 | 67.1 | 5.4 | 2.25 | 71.366 | 3.2308 |
| Algeria | Mar-07 | 0.1 | 61.2 | 67.6 | 4.6 | 2 | 71.0443 | 3.0488 |
| Algeria | Apr-07 | 1.3 | 60.6 | 67.3 | 1.0 | 1.46 | 70.5412 | 1.5083 |
| Algeria | May-07 | 1.1 | 59.9 | 67.0 | 0.4 | 0.99 | 70.4897 | 1.3616 |
| Algeria | Jun-07 | 1.7 | 62.1 | 68.0 | 5.2 | 0.79 | 70.2473 | 3.1866 |
| Algeria | Jul-07 | 1.3 | 63.0 | 68.5 | 9.3 | 0.48 | 68.9819 | 4.7401 |
| Algeria | Aug-07 | 1.3 | 63.6 | 68.9 | 8.7 | . | 67.7135 | 4.3939 |
| Algeria | Sep-07 | 1.8 | 65.8 | 70.1 | 10.6 | 0.23 | 68.1568 | 5.4135 |
| Algeria | Oct-07 | 2.0 | 66.5 | 70.4 | 9.3 | 0.14 | 67.5758 | 4.9180 |
| Algeria | Nov-07 | 0.7 | 65.2 | 69.9 | 9.0 | 0.28 | 66.8223 | 4.7976 |
| Algeria | Dec-07 | 0.1 | 65.9 | 70.3 | 8.9 | 0.33 | 67.0718 | 4.7690 |
| Algeria | Jan-08 | -0.2 | 64.7 | 69.7 | 8.4 | 1.53 | 66.8474 | 4.4978 |
| Algeria | Feb-08 | 0.8 | 65.8 | 70.3 | 9.3 | 0.63 | 66.7689 | 4.7690 |
| Algeria | Mar-08 | 1.7 | 67.9 | 71.6 | 10.9 | 0.31 | 65.6725 | 5.9172 |
| Algeria | Apr-08 | 2.4 | 67.9 | 71.6 | 12.0 | 0.24 | 64.9695 | 6.3893 |
| Algeria | May-08 | 1.7 | 67.5 | 71.5 | 12.6 | 0.17 | 63.4761 | 6.7164 |
| Algeria | Jun-08 | 1.4 | 66.1 | 70.8 | 6.5 | 0.15 | 62.3983 | 4.1176 |
| Algeria | Jul-08 | 2.4 | 65.0 | 70.3 | 3.1 | 0.13 | 61.4996 | 2.6277 |
| Algeria | Aug-08 | 1.4 | 67.2 | 72.0 | 5.5 | 0.13 | 61.0767 | 4.4993 |
| Algeria | Sep-08 | 1.7 | 69.5 | 73.3 | 5.5 | 0.09 | 60.9788 | 4.5649 |
| Algeria | Oct-08 | 1.2 | 69.4 | 73.3 | 4.5 | 0.24 | 62.2584 | 4.1193 |
| Algeria | Nov-08 | -0.2 | 69.6 | 73.6 | 6.8 | 0.2 | 68.133 | 5.2933 |
| Algeria | Dec-08 | -0.5 | 69.9 | 73.7 | 6.0 | 0.19 | 70.9144 | 4.8364 |
| Algeria | Jan-09 | 0.3 | 69.5 | 73.6 | 7.5 | 1 | 71.3942 | 5.5954 |
| Algeria | Feb-09 | 0.3 | 71.3 | 74.7 | 8.5 | 0.8 | 72.2223 | 6.2589 |
| Algeria | Mar-09 | 1.6 | 73.0 | 75.6 | 7.5 | 0.49 | 72.9287 | 5.5866 |
| Algeria | Apr-09 | 0.1 | 73.3 | 75.8 | 8.0 | 0.3 | 73.1233 | 5.8659 |
| Algeria | May-09 | 1.3 | 69.6 | 74.1 | 3.0 | 0.17 | 72.6256 | 3.6364 |
| Algeria | Jun-09 | 1.7 | 69.8 | 74.3 | 5.5 | 1.18 | 73.169 | 4.9435 |
| Algeria | Jul-09 | 2.4 | 71.6 | 75.4 | 10.2 | 1.23 | 73.2694 | 7.2546 |
| Algeria | Aug-09 | 1.5 | 74.1 | 76.7 | 10.3 | . | 72.9897 | 6.5278 |
| Algeria | Sep-09 | -0.2 | 76.0 | 77.6 | 9.4 | 0.97 | 72.5953 | 5.8663 |
| Algeria | Oct-09 | 1.7 | 76.3 | 77.8 | 9.9 | 0.5 | 72.2669 | 6.1392 |
| Algeria | Nov-09 | 1.3 | 76.4 | 77.9 | 9.7 | 0.38 | 72.2505 | 5.8424 |
| Algeria | Dec-09 | 3.1 | 76.2 | 77.9 | 9.1 | 0.35 | 72.9341 | 5.6988 |
| Algeria | Jan-10 | 2.2 | 74.9 | 77.3 | 7.7 | 0.45 | 72.9231 | 5.0272 |
| Algeria | Feb-10 | 4.0 | 75.4 | 77.8 | 5.8 | 0.38 | 73.5226 | 4.1499 |
| Algeria | Mar-10 | 3.4 | 76.3 | 78.4 | 4.5 | 0.35 | 73.4539 | 3.7037 |
| Algeria | Apr-10 | 3.8 | 74.6 | 77.6 | 1.8 | 0.33 | 73.6161 | 2.3747 |
| Algeria | May-10 | 1.1 | 76.3 | 78.7 | 9.7 | 0.31 | 74.8382 | 6.2078 |
| Algeria | Jun-10 | 1.5 | 76.5 | 78.8 | 9.7 | 0.28 | 75.6394 | 6.0565 |
| Algeria | Jul-10 | 2.0 | 74.3 | 78.4 | 3.7 | 0.29 | 74.9686 | 3.9788 |
| Algeria | Aug-10 | 1.7 | 76.9 | 79.7 | 3.7 | 0.31 | 75.4292 | 3.9113 |
| Algeria | Sep-10 | 1.3 | 76.3 | 79.5 | 0.4 | 0.3 | 75.3622 | 2.4485 |
| Algeria | Oct-10 | 1.6 | 77.1 | 79.9 | 1.1 | 0.27 | 74.246 | 2.6992 |
| Algeria | Nov-10 | 1.8 | 78.4 | 80.6 | 2.7 | 0.25 | 74.246 | 3.4660 |
| Algeria | Dec-10 | 2.6 | 77.2 | 80.1 | 1.3 | 0.24 | 74.3865 | 2.8241 |
| Algeria | Jan-11 | 1.5 | 77.0 | 80.1 | 2.9 | 0.23 | 73.3933 | 3.6223 |
| Algeria | Feb-11 | -0.7 | 77.5 | 80.5 | 2.7 | 0.23 | 72.8748 | 3.4704 |
| Algeria | Mar-11 | 1.8 | 77.8 | 81.1 | 2.0 | 0.22 | 72.3133 | 3.4439 |
| Algeria | Apr-11 | 2.2 | 77.8 | 81.2 | 4.3 | 0.22 | 71.7144 | 4.6392 |
| Algeria | May-11 | 1.1 | 78.9 | 81.8 | 3.4 | 0.22 | 72.1663 | 3.9390 |
| Algeria | Jun-11 | 0.8 | 78.3 | 81.7 | 2.4 | 0.22 | 71.9411 | 3.6802 |
| Algeria | Jul-11 | 1.8 | 79.4 | 82.5 | 6.8 | 0.2 | 72.1261 | 5.2296 |
| Algeria | Aug-11 | 1.5 | 82.4 | 84.3 | 7.1 | 0.2 | 72.0939 | 5.7716 |
| Algeria | Sep-11 | 2.5 | 80.6 | 83.6 | 5.7 | 0.18 | 73.3364 | 5.1572 |
| Algeria | Oct-11 | 0.3 | 81.9 | 84.3 | 6.1 | 0.17 | 73.6152 | 5.5069 |
| Algeria | Nov-11 | 1.4 | 81.1 | 84.6 | 3.5 | 0.2 | 74.8399 | 4.9628 |
| Algeria | Dec-11 | 0.3 | 80.1 | 84.2 | 3.6 | 0.25 | 74.8399 | 5.1186 |
| Algeria | Jan-12 | -1.2 | 83.8 | 86.1 | 8.8 | 0.41 | 76.2329 | 7.4906 |
| Algeria | Feb-12 | -2.8 | 86.6 | 88.0 | 11.8 | 0.43 | 74.7916 | 9.3168 |
| Algeria | Mar-12 | 0.1 | 88.9 | 89.3 | 14.2 | 0.51 | 74.4193 | 10.1110 |
| Algeria | Apr-12 | 1.1 | 90.2 | 90.1 | 15.9 | 0.55 | 74.1482 | 10.9606 |
| Algeria | May-12 | 2.4 | 87.8 | 89.1 | 11.3 | 0.58 | 75.0036 | 8.9242 |
| Algeria | Jun-12 | 3.1 | 86.8 | 88.8 | 10.8 | 0.6 | 77.7197 | 8.6903 |
| Algeria | Jul-12 | 2.3 | 87.8 | 89.3 | 10.7 | 0.63 | 80.6906 | 8.2424 |
| Algeria | Aug-12 | 1.8 | 89.9 | 90.3 | 9.2 | 0.64 | 81.1461 | 7.1174 |
| Algeria | Sep-12 | 1.2 | 90.8 | 90.9 | 12.5 | 0.6 | 79.4696 | 8.7321 |
| Algeria | Oct-12 | 2.7 | 94.4 | 92.7 | 15.3 | 0.45 | 79.2075 | 9.9644 |
| Algeria | Nov-12 | 2.9 | 90.8 | 91.4 | 12.0 | 0.27 | 79.4157 | 8.0378 |
| Algeria | Dec-12 | 0.7 | 91.5 | 91.8 | 14.3 | 0.2 | 78.1868 | 9.0261 |
| Algeria | Jan-13 | 0.2 | 92.6 | 92.9 | 10.5 | 0.17 | 77.9321 | 7.8978 |
| Algeria | Feb-13 | -0.9 | 91.8 | 92.7 | 6.0 | 0.19 | 77.8611 | 5.3409 |
| Algeria | Mar-13 | 2.9 | 92.9 | 93.2 | 4.6 | 0.51 | 78.7383 | 4.3673 |
| Algeria | Apr-13 | 2.1 | 92.0 | 92.8 | 2.0 | 0.22 | 78.6664 | 2.9967 |
| Algeria | May-13 | 1.3 | 90.6 | 92.2 | 3.2 | 0.23 | 78.8896 | 3.4792 |
| Algeria | Jun-13 | 0.8 | 91.9 | 92.8 | 5.9 | 0.23 | 78.7423 | 4.5045 |
| Algeria | Jul-13 | 1.6 | 91.5 | 92.4 | 4.2 | 0.23 | 79.384 | 3.4714 |
| Algeria | Aug-13 | 0.4 | 93.0 | 93.3 | 3.5 | 0.24 | 80.3006 | 3.3223 |
| Algeria | Sep-13 | 1.0 | 91.8 | 92.9 | 1.2 | 0.24 | 81.5903 | 2.2002 |
| Algeria | Oct-13 | 3.8 | 91.5 | 92.8 | -3.0 | 0.23 | 81.3421 | 0.1079 |
| Algeria | Nov-13 | 0.7 | 90.9 | 92.0 | 0.1 | 0.24 | 80.3171 | 0.6565 |
| Algeria | Dec-13 | -0.1 | 92.6 | 92.9 | 1.2 | 0.24 | 78.6569 | 1.1983 |
| Algeria | Jan-14 | 1.4 | 93.8 | 93.5 | 1.4 | 0.24 | 78.1073 | 0.6459 |
| Algeria | Feb-14 | 1.0 | 92.4 | 93.3 | 0.7 | 0.23 | 77.9306 | 0.6472 |
| Algeria | Mar-14 | 0.3 | 93.5 | 94.0 | 0.6 | 0.23 | 77.644 | 0.8584 |
| Algeria | Apr-14 | 2.8 | 92.2 | 93.5 | 0.1 | 0.24 | 78.5992 | 0.7543 |
| Algeria | May-14 | 2.4 | 92.5 | 93.7 | 2.1 | 0.28 | 78.7997 | 1.6269 |
| Algeria | Jun-14 | 0.8 | 95.8 | 95.4 | 4.3 | 0.27 | 79.294 | 2.8017 |
| Algeria | Jul-14 | 2.1 | 95.1 | 95.5 | 3.9 | 0.3 | 79.8322 | 3.3550 |
| Algeria | Aug-14 | 2.2 | 95.5 | 95.8 | 2.7 | 0.3 | 80.3249 | 2.6795 |
| Algeria | Sep-14 | 2.9 | 96.7 | 96.5 | 5.3 | 0.32 | 81.1859 | 3.8751 |
| Algeria | Oct-14 | 2.4 | 100.7 | 98.5 | 10.0 | 0.34 | 83.4736 | 6.1422 |
| Algeria | Nov-14 | 2.3 | 99.3 | 97.9 | 9.2 | 0.35 | 84.7706 | 6.4130 |
| Algeria | Dec-14 | -0.1 | 98.7 | 97.7 | 6.6 | 0.35 | 86.9862 | 5.1668 |
| Algeria | Jan-15 | -0.5 | 99.1 | 97.8 | 5.6 | 0.37 | 92.9012 | 4.5989 |
| Algeria | Feb-15 | -0.6 | 99.4 | 98.5 | 7.5 | 0.38 | 93.9586 | 5.5734 |
| Algeria | Mar-15 | -0.3 | 100.2 | 99.2 | 7.3 | 0.42 | 96.4794 | 5.5319 |
| Algeria | Apr-15 | 3.1 | 98.9 | 98.8 | 7.4 | 0.44 | 98.0243 | 5.6684 |
| Algeria | May-15 | 3.1 | 99.4 | 99.3 | 7.5 | 0.46 | 98.3472 | 5.9765 |
| Algeria | Jun-15 | 0.9 | 100.0 | 99.7 | 4.4 | 0.5 | 98.5561 | 4.5073 |
| Algeria | Jul-15 | 1.0 | 97.5 | 99.0 | 2.5 | 0.53 | 99.5452 | 3.6649 |
| Algeria | Aug-15 | 1.8 | 99.1 | 100.1 | 3.7 | 0.53 | 103.4645 | 4.4885 |
| Algeria | Sep-15 | 1.4 | 103.4 | 102.4 | 6.9 | 0.55 | 105.9867 | 6.1140 |
| Algeria | Oct-15 | 2.7 | 102.1 | 102.0 | 1.4 | 0.57 | 105.9767 | 3.5533 |
| Algeria | Nov-15 | 1.2 | 99.9 | 101.3 | 0.6 | 0.6 | 107.7834 | 3.4729 |
| Algeria | Dec-15 | 0.9 | 101.1 | 102.0 | 2.4 | 0.62 | 107.2739 | 4.4012 |
| Algeria | Jan-16 | 2.3 | 101.1 | 102.8 | 2.0 | 0.63 | 107.4411 | 5.1125 |
| Algeria | Feb-16 | 1.6 | 99.2 | 102.6 | -0.2 | 0.66 | 106.5487 | 4.1624 |
| Algeria | Mar-16 | 0.0 | 101.2 | 104.5 | 0.9 | 0.79 | 109.5423 | 5.3427 |
| Algeria | Apr-16 | 3.6 | 102.9 | 105.5 | 4.0 | 0.91 | 108.7676 | 6.7814 |
| Algeria | May-16 | 2.5 | 103.2 | 106.2 | 3.9 | 1.06 | 109.787 | 6.9486 |
| Algeria | Jun-16 | 1.9 | 105.9 | 107.6 | 5.9 | 1.24 | 110.1451 | 7.9238 |
| Algeria | Jul-16 | 1.4 | 104.5 | 107.1 | 7.1 | 1.36 | 110.6086 | 8.1818 |
| Algeria | Aug-16 | 0.8 | 104.5 | 107.6 | 5.5 | 1.49 | 109.4703 | 7.4925 |
| Algeria | Sep-16 | 1.6 | 104.8 | 108.0 | 1.3 | 1.55 | 109.3162 | 5.4688 |
| Algeria | Oct-16 | 3.3 | 102.9 | 107.2 | 0.8 | 1.55 | 110.1722 | 5.0980 |
| Algeria | Nov-16 | 1.6 | 105.3 | 108.9 | 5.4 | 1.55 | 110.6175 | 7.5025 |
| Algeria | Dec-16 | 1.7 | 105.4 | 109.1 | 4.3 | 1.55 | 110.9002 | 6.9608 |
| Algeria | Jan-17 | -1.7 | 108.0 | 111.1 | 6.9 | 1.55 | 110.0475 | 8.0739 |
| Algeria | Feb-17 | 2.0 | 106.4 | 110.4 | 7.2 | 1.55 | 109.9259 | 7.6023 |
| Algeria | Mar-17 | 2.0 | 108.3 | 111.7 | 7.1 | 1.55 | 109.8772 | 6.8900 |
| Algeria | Apr-17 | 2.1 | 108.8 | 112.1 | 5.8 | 1.55 | 109.9478 | 6.2559 |
| Algeria | May-17 | 4.0 | 107.6 | 111.7 | 4.2 | 1.55 | 108.937 | 5.1789 |
| Algeria | Jun-17 | 2.0 | 107.9 | 112.0 | 1.8 | 1.55 | 108.4008 | 4.0892 |
| Algeria | Jul-17 | 1.7 | 104.3 | 110.4 | -0.1 | 1.56 | 108.7211 | 3.0812 |
| Algeria | Aug-17 | 1.6 | 108.1 | 112.4 | 3.5 | 1.75 | 109.6243 | 4.4610 |
| Algeria | Sep-17 | 1.4 | 110.3 | 113.6 | 5.3 | 2 | 111.7448 | 5.1852 |
| Algeria | Oct-17 | 0.9 | 111.4 | 114.2 | 8.3 | 2.07 | 114.1349 | 6.5299 |
| Algeria | Nov-17 | 0.7 | 110.7 | 114.2 | 5.2 | 2.1 | 115.073 | 4.8669 |
| Algeria | Dec-17 | -0.2 | 111.0 | 114.5 | 5.3 | 2.17 | 115.2419 | 4.9496 |
| Algeria | Jan-18 | 1.5 | 110.0 | 115.0 | 1.8 | 2.2 | 114.2968 | 3.5104 |
| Algeria | Feb-18 | -0.4 | 107.9 | 114.4 | 1.4 | 2.23 | 113.8607 | 3.6232 |
| Algeria | Mar-18 | 2.0 | 109.4 | 115.2 | 1.0 | 2.25 | 114.0351 | 3.1334 |
| Algeria | Apr-18 | 2.1 | 110.7 | 115.9 | 1.7 | 2.25 | 114.2165 | 3.3898 |
| Algeria | May-18 | 0.9 | 114.8 | 118.0 | 6.7 | 2.28 | 115.974 | 5.6401 |
| Algeria | Jun-18 | 1.0 | 117.1 | 119.3 | 8.5 | 2.3 | 116.9667 | 6.5179 |
| Algeria | Jul-18 | 3.4 | 111.8 | 117.0 | 7.2 | 2.38 | 117.6772 | 5.9783 |
| Algeria | Aug-18 | 0.5 | 112.5 | 117.5 | 4.0 | 2.43 | 118.3993 | 4.5374 |
| Algeria | Sep-18 | 1.4 | 112.8 | 117.9 | 2.2 | 2.49 | 117.9911 | 3.7852 |
| Algeria | Oct-18 | 1.2 | 115.4 | 119.3 | 3.6 | 2.5 | 118.67 | 4.4658 |
| Algeria | Nov-18 | 1.5 | 113.8 | 118.8 | 2.8 | 2.5 | 118.5182 | 4.0280 |
| Algeria | Dec-18 | 1.1 | 110.9 | 117.6 | -0.1 | 2.5 | 118.5199 | 2.7074 |
| Algeria | Jan-19 | -0.9 | 111.5 | 118.0 | 1.3 | 2.5 | 118.3305 | 2.6087 |
| Algeria | Feb-19 | -1.2 | 110.0 | 117.4 | 2.0 | 2.69 | 118.6176 | 2.6224 |
| Algeria | Mar-19 | 0.7 | 112.0 | 118.5 | 2.4 | 2.9 | 118.9239 | 2.8646 |
| Algeria | Apr-19 | 1.0 | 111.8 | 118.6 | 1.0 | 2.92 | 119.3079 | 2.3296 |
| Algeria | May-19 | 1.6 | 113.1 | 119.7 | -1.5 | 2.94 | 119.5117 | 1.4407 |
| Algeria | Jun-19 | 1.7 | 111.9 | 119.4 | -4.4 | 2.97 | 119.0572 | 0.0838 |
| Algeria | Jul-19 | 2.2 | 109.2 | 118.6 | -2.4 | 2.98 | 119.2523 | 1.3675 |
| Algeria | Aug-19 | 2.2 | 111.3 | 119.8 | -1.1 | 2.98 | 119.6585 | 1.9574 |
| Algeria | Sep-19 | 2.0 | 113.9 | 121.3 | 1.0 | 2.97 | 120.1191 | 2.8838 |
| Algeria | Oct-19 | 2.0 | 113.3 | 121.1 | -1.8 | 3 | 119.9325 | 1.5088 |
| Algeria | Nov-19 | 0.8 | 111.0 | 120.4 | -2.5 | 3 | 119.8986 | 1.3468 |
| Algeria | Dec-19 | 2.2 | 110.6 | 120.4 | -0.2 | 3 | 119.6329 | 2.3810 |
| Algeria | Jan-20 | 0.0 | 110.2 | 120.3 | -1.2 | 3 | 119.6404 | 1.9492 |
| Algeria | Feb-20 | 2.2 | 108.4 | 119.6 | -1.4 | 3 | 120.5274 | 1.8739 |
| Algeria | Mar-20 | 1.6 | 111.1 | 121.1 | -0.8 | 3 | 121.3511 | 2.1941 |
| Algeria | Apr-20 | 2.9 | 111.7 | 121.4 | -0.1 | 3 | 127.5983 | 2.3609 |
| Algeria | May-20 | 2.8 | 114.0 | 122.6 | 0.8 | 3 | 128.6924 | 2.4227 |
| Algeria | Jun-20 | 2.0 | 113.8 | 122.8 | 1.7 | 3 | 128.7417 | 2.8476 |
| Algeria | Jul-20 | 1.8 | 109.9 | 121.4 | 0.6 | 3 | 128.4361 | 2.3609 |
| Algeria | Aug-20 | 2.1 | 109.2 | 121.6 | -1.9 | 3 | 128.3389 | 1.5025 |
| Algeria | Sep-20 | 1.8 | 111.6 | 123.0 | -2.0 | 3 | 128.7995 | 1.4015 |
| Algeria | Oct-20 | 1.9 | 115.4 | 125.0 | 1.8 | 3 | 128.9041 | 3.2205 |
| Algeria | Nov-20 | 1.5 | 113.6 | 124.5 | 2.4 | 3 | 128.6956 | 3.4053 |
| Algeria | Dec-20 | 1.7 | 113.3 | 124.7 | 2.5 | 3 | 131.5961 | 3.5714 |
| Algeria | Jan-21 | 2.0 | 114.5 | 125.4 | 3.9 | 3 | 132.6737 | 4.2394 |
| Algeria | Feb-21 | 2.1 | 115.3 | 125.9 | 6.3 | 3 | 132.9357 | 5.2676 |
| Algeria | Mar-21 | 0.8 | 119.5 | 128.2 | 7.5 | 3 | 133.7369 | 5.8629 |
| Algeria | Apr-21 | 3.1 | 122.6 | 130.1 | 9.7 | 3 | 132.9544 | 7.1664 |
| Algeria | May-21 | 3.6 | 121.8 | 130.0 | 6.8 | 3 | 133.5319 | 6.0359 |
| Algeria | Jun-21 | 3.2 | 120.2 | 129.4 | 5.6 | 3 | 134.0023 | 5.3746 |
| Algeria | Jul-21 | 2.9 | 121.4 | 130.3 | 10.5 | 3 | 134.8851 | 7.3311 |
| Algeria | Aug-21 | 2.5 | 124.4 | 132.2 | 13.9 | 3 | 135.338 | 8.7171 |
| Algeria | Sep-21 | 3.3 | 129.1 | 134.8 | 15.7 | 3 | 136.4595 | 9.5935 |
| Algeria | Oct-21 | 1.8 | 132.1 | 136.5 | 14.4 | 3 | 137.1327 | 9.2000 |
| Algeria | Nov-21 | 1.3 | 130.0 | 136.1 | 14.5 | 3 | 138.2231 | 9.3173 |
| Algeria | Dec-21 | 1.5 | 127.5 | 135.2 | 12.5 | 3 | 138.8954 | 8.4202 |
| Algeria | Jan-22 | -0.5 | 129.7 | 136.7 | 13.3 | 3 | 139.5433 | 9.0112 |
| Algeria | Feb-22 | 1.3 | 130.8 | 137.5 | 13.4 | 2.99 | 140.5461 | 9.2137 |
| Algeria | Mar-22 | 1.2 | 135.5 | 140.1 | 13.5 | 2.99 | 142.4888 | 9.2824 |
| Algeria | Apr-22 | 1.4 | 141.4 | 143.1 | 15.3 | 2.99 | 143.5684 | 9.9923 |
| Algeria | May-22 | 1.6 | 138.9 | 142.4 | 14.1 | 2.99 | 145.5707 | 9.5385 |
| Algeria | Jun-22 | 3.0 | 140.5 | 143.4 | 16.9 | 2.99 | 145.8061 | 10.8192 |
| Algeria | Jul-22 | 1.8 | 137.7 | 142.5 | 13.4 | 2.99 | 146.3142 | 9.3630 |
| Algeria | Aug-22 | 2.2 | 142.6 | 145.2 | 14.6 | 2.99 | 142.4505 | 9.8336 |
| Algeria | Sep-22 | 3.1 | 143.9 | 146.4 | 11.4 | 2.99 | 140.5599 | 8.6053 |
| Algeria | Oct-22 | 2.9 | 145.9 | 147.5 | 10.5 | 2.99 | 140.2631 | 8.0586 |
| Algeria | Nov-22 | 1.9 | 144.5 | 147.2 | 11.2 | 2.99 | 139.2511 | 8.1558 |
| Algeria | Dec-22 | 4.1 | 144.8 | 147.8 | 13.6 | 2.99 | 137.5775 | 9.3195 |
| Algeria | Jan-23 | 0.0 | 147.5 | 149.4 | 13.7 | 2.99 | 136.2533 | 9.2904 |
| Algeria | Feb-23 | 0.1 | 150.4 | 151.1 | 15.0 | 2.99 | 136.4209 | 9.8909 |
| Algeria | Mar-23 | 3.4 | 155.8 | 154.2 | 14.9 | 2.99 | 135.9905 | 10.0642 |
| Algeria | Apr-23 | 2.9 | 161.3 | 157.3 | 14.1 | 2.99 | 135.4444 | 9.9231 |
| Algeria | May-23 | 0.7 | 159.4 | 156.8 | 14.8 | 2.99 | 135.9522 | 10.1124 |
| Algeria | Jun-23 | 1.5 | 157.9 | 156.5 | 12.4 | 2.99 | 135.9001 | 9.1353 |
| Algeria | Jul-23 | 3.7 | 155.9 | 155.9 | 13.2 | 2.99 | 135.0427 | 9.4035 |
| Algeria | Aug-23 | 1.9 | 162.4 | 159.3 | 13.9 | 2.99 | 136.0441 | 9.7107 |
| Algeria | Sep-23 | 2.5 | 163.9 | 160.4 | 13.9 | 2.99 | 137.0188 | 9.5628 |
| Algeria | Oct-23 | 3.5 | 161.9 | 159.6 | 10.9 | 2.99 | 137.1677 | 8.2034 |
| Algeria | Nov-23 | 3.0 | 162.3 | 160.4 | 12.3 | 2.99 | 134.548 | 8.9674 |
| Algeria | Dec-23 | 1.7 | 159.7 | 159.4 | 10.3 | 2.97 | 134.3325 | 7.8484 |
| Egypt | Jan-01 | 0.9 | 20.5 | 29.2 | 0.8 | 9.1 | 3.7 | 2.8169 |
| Egypt | Feb-01 | -0.5 | 20.5 | 29.2 | 1.1 | 9.1 | 3.84 | 2.4561 |
| Egypt | Mar-01 | 2.6 | 20.6 | 29.2 | 1.1 | 9.1 | 3.84 | 2.0979 |
| Egypt | Apr-01 | 0.7 | 20.6 | 29.3 | 0.7 | 9.1 | 3.84 | 2.4476 |
| Egypt | May-01 | 0.5 | 20.6 | 29.3 | 0.6 | 9 | 3.841 | 2.0906 |
| Egypt | Jun-01 | -0.4 | 20.7 | 29.4 | 0.9 | 9.1 | 3.85 | 2.4390 |
| Egypt | Jul-01 | 0.9 | 20.8 | 29.5 | 1.1 | 9 | 3.85 | 2.4306 |
| Egypt | Aug-01 | 1.7 | 20.8 | 29.5 | 1.0 | 8.8 | 4.14 | 2.0761 |
| Egypt | Sep-01 | 1.4 | 20.9 | 29.5 | 1.0 | 8.7 | 4.14 | 2.0761 |
| Egypt | Oct-01 | -0.5 | 21.0 | 29.6 | 1.7 | 8.6 | 4.14 | 2.4221 |
| Egypt | Nov-01 | -0.3 | 20.9 | 29.6 | 1.1 | 7.9 | 4.14 | 2.0690 |
| Egypt | Dec-01 | 0.0 | 21.0 | 29.7 | 1.8 | 7.2 | 4.355 | 2.4138 |
| Egypt | Jan-02 | -1.1 | 21.2 | 29.9 | 3.6 | 7.2 | 4.496 | 2.3973 |
| Egypt | Feb-02 | 1.2 | 21.2 | 29.9 | 3.5 | 7.2 | 4.5 | 2.3973 |
| Egypt | Mar-02 | 1.3 | 21.4 | 30.0 | 3.7 | 7.2 | 4.5 | 2.7397 |
| Egypt | Apr-02 | 0.0 | 21.4 | 30.0 | 4.1 | 7.2 | 4.5 | 2.3891 |
| Egypt | May-02 | -0.1 | 21.5 | 30.1 | 4.4 | 7.2 | 4.5 | 2.7304 |
| Egypt | Jun-02 | -0.3 | 21.5 | 30.2 | 4.2 | 7.2 | 4.5 | 2.7211 |
| Egypt | Jul-02 | 2.3 | 21.6 | 30.2 | 3.9 | 7.2 | 4.5 | 2.3729 |
| Egypt | Aug-02 | 1.7 | 21.7 | 30.3 | 4.1 | 7.1 | 4.5 | 2.7119 |
| Egypt | Sep-02 | 2.3 | 21.8 | 30.4 | 4.6 | 7 | 4.5 | 3.0508 |
| Egypt | Oct-02 | 0.8 | 21.9 | 30.4 | 4.3 | 6.6 | 4.5 | 2.7027 |
| Egypt | Nov-02 | 1.1 | 22.0 | 30.6 | 5.3 | 6.1 | 4.5 | 3.3784 |
| Egypt | Dec-02 | 0.7 | 22.0 | 30.6 | 4.9 | 5.5 | 4.5 | 3.0303 |
| Egypt | Jan-03 | 2.1 | 22.2 | 30.8 | 4.4 | 6.3 | 4.5586 | 3.0100 |
| Egypt | Feb-03 | -1.4 | 22.3 | 30.9 | 4.9 | 7.5 | 5.4635 | 3.3445 |
| Egypt | Mar-03 | -2.0 | 22.5 | 31.1 | 5.4 | 13.6 | 5.6505 | 3.6667 |
| Egypt | Apr-03 | 0.4 | 22.7 | 31.2 | 6.0 | 12.1 | 5.8199 | 4.0000 |
| Egypt | May-03 | 1.4 | 22.9 | 31.3 | 6.5 | 10.1 | 5.9377 | 3.9867 |
| Egypt | Jun-03 | 0.9 | 23.0 | 31.4 | 6.7 | 10.3 | 5.9868 | 3.9735 |
| Egypt | Jul-03 | 0.9 | 23.1 | 31.5 | 7.1 | 9.9 | 6.094 | 4.3046 |
| Egypt | Aug-03 | 1.3 | 23.2 | 31.7 | 7.1 | 9 | 6.1408 | 4.6205 |
| Egypt | Sep-03 | 1.1 | 23.5 | 32.0 | 7.5 | 7.4 | 6.1397 | 5.2632 |
| Egypt | Oct-03 | 1.5 | 23.6 | 32.2 | 7.8 | 6.9 | 6.1358 | 5.9211 |
| Egypt | Nov-03 | 1.0 | 23.7 | 32.4 | 7.5 | 7.1 | 6.1352 | 5.8824 |
| Egypt | Dec-03 | 0.0 | 23.9 | 32.6 | 8.4 | 6.9 | 6.148 | 6.5359 |
| Egypt | Jan-04 | 0.4 | 24.6 | 33.2 | 11.2 | 6.8 | 6.1568 | 7.7922 |
| Egypt | Feb-04 | 0.4 | 25.8 | 33.9 | 15.6 | 7 | 6.1674 | 9.7087 |
| Egypt | Mar-04 | 1.4 | 26.6 | 34.5 | 18.2 | 7.9 | 6.1772 | 10.9325 |
| Egypt | Apr-04 | 0.0 | 26.9 | 35.0 | 18.4 | 9.5 | 6.1819 | 12.1795 |
| Egypt | May-04 | 1.3 | 26.9 | 35.0 | 17.5 | 10.5 | 6.1904 | 11.8211 |
| Egypt | Jun-04 | -0.3 | 26.9 | 35.1 | 17.1 | 11.3 | 6.1938 | 11.7834 |
| Egypt | Jul-04 | 1.0 | 26.9 | 35.3 | 16.1 | 11.2 | 6.1973 | 12.0635 |
| Egypt | Aug-04 | 0.3 | 26.7 | 35.3 | 15.2 | 10.9 | 6.2065 | 11.3565 |
| Egypt | Sep-04 | 0.5 | 25.5 | 35.8 | 8.6 | 10.7 | 6.2175 | 11.8750 |
| Egypt | Oct-04 | 0.9 | 26.2 | 36.2 | 11.1 | 10.9 | 6.2322 | 12.4224 |
| Egypt | Nov-04 | 0.9 | 26.0 | 36.2 | 9.7 | 10.2 | 6.2264 | 11.7284 |
| Egypt | Dec-04 | 0.2 | 26.1 | 36.3 | 9.3 | 9.9 | 6.2075 | 11.3497 |
| Egypt | Jan-05 | 0.7 | 26.0 | 36.4 | 5.5 | 9.7 | 5.8726 | 9.6386 |
| Egypt | Feb-05 | 0.8 | 25.7 | 36.2 | -0.1 | 9.1 | 5.7937 | 6.7847 |
| Egypt | Mar-05 | 0.6 | 26.0 | 36.4 | -2.2 | 10.8 | 5.7896 | 5.5072 |
| Egypt | Apr-05 | 0.6 | 26.2 | 36.7 | -2.7 | 10.19 | 5.7867 | 4.8571 |
| Egypt | May-05 | -0.6 | 26.4 | 36.8 | -1.8 | 9.8 | 5.7866 | 5.1429 |
| Egypt | Jun-05 | 0.0 | 26.3 | 36.7 | -2.4 | 9.1 | 5.7867 | 4.5584 |
| Egypt | Jul-05 | 1.0 | 26.4 | 36.9 | -1.6 | 9 | 5.7695 | 4.5326 |
| Egypt | Aug-05 | 1.4 | 26.5 | 36.9 | -0.8 | 9.3 | 5.7624 | 4.5326 |
| Egypt | Sep-05 | 1.4 | 26.8 | 37.1 | 5.3 | 9.2 | 5.7573 | 3.6313 |
| Egypt | Oct-05 | -0.2 | 27.1 | 37.3 | 3.3 | 9.316 | 5.751 | 3.0387 |
| Egypt | Nov-05 | -0.7 | 27.1 | 37.4 | 4.6 | 9.104 | 5.7519 | 3.3149 |
| Egypt | Dec-05 | 1.8 | 27.2 | 37.4 | 4.1 | 8.572 | 5.738 | 3.0303 |
| Egypt | Jan-06 | 1.0 | 27.3 | 37.6 | 5.0 | 8.095 | 5.7275 | 3.2967 |
| Egypt | Feb-06 | 1.2 | 27.4 | 37.7 | 6.3 | 8.05 | 5.7257 | 4.1436 |
| Egypt | Mar-06 | 0.8 | 27.4 | 37.8 | 5.2 | 8.613 | 5.7314 | 3.8462 |
| Egypt | Apr-06 | -0.1 | 27.6 | 38.3 | 5.7 | 8.663 | 5.7396 | 4.3597 |
| Egypt | May-06 | -0.2 | 28.4 | 38.8 | 7.7 | 8.662 | 5.755 | 5.4348 |
| Egypt | Jun-06 | 0.6 | 29.3 | 39.4 | 11.6 | 8.843 | 5.7534 | 7.3569 |
| Egypt | Jul-06 | 0.3 | 29.3 | 40.0 | 10.9 | 8.861 | 5.7414 | 8.4011 |
| Egypt | Aug-06 | 1.9 | 29.2 | 40.2 | 10.2 | 8.846 | 5.7367 | 8.9431 |
| Egypt | Sep-06 | 1.6 | 30.0 | 40.6 | 11.7 | 9.327 | 5.7326 | 9.4340 |
| Egypt | Oct-06 | 0.2 | 31.1 | 41.7 | 15.1 | 9.415 | 5.7299 | 11.7962 |
| Egypt | Nov-06 | -1.5 | 31.3 | 41.9 | 15.4 | 9.453 | 5.7162 | 12.0321 |
| Egypt | Dec-06 | -0.6 | 31.5 | 42.1 | 16.0 | 9.534 | 5.7086 | 12.5668 |
| Egypt | Jan-07 | -0.1 | 31.5 | 42.2 | 15.4 | 9.442 | 5.6971 | 12.2340 |
| Egypt | Feb-07 | 0.3 | 31.8 | 42.4 | 16.3 | 8.372 | 5.6906 | 12.4668 |
| Egypt | Mar-07 | 0.4 | 32.1 | 42.6 | 17.1 | 8.285 | 5.6921 | 12.6984 |
| Egypt | Apr-07 | 0.2 | 32.2 | 42.7 | 16.6 | 7.531 | 5.6836 | 11.4883 |
| Egypt | May-07 | 1.0 | 32.1 | 42.6 | 12.7 | 7.12 | 5.6822 | 9.7938 |
| Egypt | Jun-07 | 0.7 | 32.3 | 42.7 | 10.1 | 6.781 | 5.6886 | 8.3756 |
| Egypt | Jul-07 | 1.0 | 32.6 | 43.0 | 11.3 | 6.976 | 5.6721 | 7.5000 |
| Egypt | Aug-07 | 1.3 | 33.3 | 43.5 | 14.0 | 7.817 | 5.6477 | 8.2090 |
| Egypt | Sep-07 | 0.2 | 34.9 | 44.3 | 16.4 | 7.203 | 5.619 | 9.1133 |
| Egypt | Oct-07 | 1.1 | 34.2 | 44.8 | 9.9 | 6.924 | 5.5322 | 7.4341 |
| Egypt | Nov-07 | 0.7 | 34.0 | 44.7 | 8.5 | 7.124 | 5.5 | 6.6826 |
| Egypt | Dec-07 | 0.3 | 34.3 | 44.9 | 8.6 | 6.852 | 5.52 | 6.6508 |
| Egypt | Jan-08 | -0.6 | 35.7 | 46.7 | 13.5 | 5.993 | 5.5 | 10.6635 |
| Egypt | Feb-08 | -1.2 | 37.2 | 47.5 | 16.8 | 5.258 | 5.5 | 12.0283 |
| Egypt | Mar-08 | 3.0 | 38.7 | 48.7 | 20.5 | 5.733 | 5.46 | 14.3192 |
| Egypt | Apr-08 | 1.1 | 39.3 | 49.7 | 22.0 | 5.943 | 5.42 | 16.3934 |
| Egypt | May-08 | 0.3 | 40.7 | 51.0 | 27.0 | 9.21 | 5.34 | 19.7183 |
| Egypt | Jun-08 | 1.5 | 41.0 | 51.3 | 27.1 | 9.675 | 5.34 | 20.1405 |
| Egypt | Jul-08 | 1.1 | 42.3 | 52.5 | 29.9 | 10.306 | 5.31 | 22.0930 |
| Egypt | Aug-08 | 1.6 | 43.6 | 53.7 | 30.9 | 12.115 | 5.33 | 23.4483 |
| Egypt | Sep-08 | 2.0 | 43.7 | 53.9 | 25.4 | 13.497 | 5.42 | 21.6704 |
| Egypt | Oct-08 | -0.2 | 43.1 | 53.8 | 25.9 | 12.762 | 5.55 | 20.0893 |
| Egypt | Nov-08 | 1.2 | 43.0 | 53.8 | 26.4 | 11.901 | 5.52 | 20.3579 |
| Egypt | Dec-08 | 1.7 | 41.8 | 53.1 | 21.9 | 11.369 | 5.5 | 18.2628 |
| Egypt | Jan-09 | 1.6 | 41.5 | 53.4 | 16.2 | 11.369 | 5.52 | 14.3469 |
| Egypt | Feb-09 | 0.7 | 42.5 | 53.9 | 14.4 | 10.511 | 5.57 | 13.4737 |
| Egypt | Mar-09 | -0.7 | 43.6 | 54.6 | 12.7 | 10.259 | 5.63 | 12.1150 |
| Egypt | Apr-09 | 1.3 | 44.7 | 55.5 | 13.7 | 10.413 | 5.62 | 11.6700 |
| Egypt | May-09 | -0.8 | 45.8 | 56.2 | 12.5 | 10.176 | 5.61 | 10.1961 |
| Egypt | Jun-09 | 1.2 | 46.0 | 56.4 | 12.2 | 10.156 | 5.6 | 9.9415 |
| Egypt | Jul-09 | 1.5 | 48.0 | 57.7 | 13.4 | 10.179 | 5.56568 | 9.9048 |
| Egypt | Aug-09 | 0.6 | 49.5 | 59.1 | 13.4 | 9.629 | 5.52811818 | 10.0559 |
| Egypt | Sep-09 | 1.3 | 51.3 | 60.1 | 17.4 | 9.611 | 5.50396 | 11.5028 |
| Egypt | Oct-09 | 1.5 | 52.6 | 60.9 | 22.2 | 9.715 | 5.46267 | 13.1970 |
| Egypt | Nov-09 | 0.2 | 52.5 | 60.8 | 22.1 | 10.116 | 5.450355 | 13.0112 |
| Egypt | Dec-09 | 1.5 | 51.1 | 60.3 | 22.3 | 9.842 | 5.47385652 | 13.5593 |
| Egypt | Jan-10 | 3.1 | 51.6 | 60.7 | 24.2 | 9.708 | 5.43729474 | 13.6704 |
| Egypt | Feb-10 | 3.3 | 52.0 | 60.8 | 22.3 | 9.629 | 5.471595 | 12.8015 |
| Egypt | Mar-10 | 2.8 | 52.7 | 61.2 | 21.0 | 9.707 | 5.47502174 | 12.0879 |
| Egypt | Apr-10 | 1.7 | 53.6 | 61.6 | 19.9 | 9.743 | 5.51184444 | 10.9910 |
| Egypt | May-10 | 0.5 | 54.2 | 61.9 | 18.2 | 10.208 | 5.60420455 | 10.1423 |
| Egypt | Jun-10 | 1.6 | 54.6 | 62.2 | 18.6 | 10.179 | 5.66435455 | 10.2837 |
| Egypt | Jul-10 | 1.7 | 56.7 | 63.7 | 18.1 | 10.159 | 5.6853 | 10.3986 |
| Egypt | Aug-10 | 2.5 | 60.3 | 65.6 | 21.8 | 9.662 | 5.6811 | 10.9983 |
| Egypt | Sep-10 | 2.3 | 62.6 | 66.7 | 21.9 | 9.422 | 5.6909 | 10.9817 |
| Egypt | Oct-10 | 3.0 | 62.4 | 67.6 | 18.6 | 9.348 | 5.7148 | 11.0016 |
| Egypt | Nov-10 | 2.8 | 61.1 | 67.0 | 16.3 | 8.681 | 5.7442 | 10.1974 |
| Egypt | Dec-10 | 2.0 | 59.9 | 66.6 | 17.3 | 9.277 | 5.7827 | 10.4478 |
| Egypt | Jan-11 | 1.2 | 61.3 | 67.3 | 18.9 | 9.499 | 5.7973 | 10.8731 |
| Egypt | Feb-11 | 1.6 | 61.5 | 67.3 | 18.2 | 10.941 | 5.8835 | 10.6908 |
| Egypt | Mar-11 | 0.1 | 63.5 | 68.3 | 20.5 | 11.201 | 5.9187 | 11.6013 |
| Egypt | Apr-11 | -0.3 | 65.2 | 69.1 | 21.7 | 11.348 | 5.9466 | 12.1753 |
| Egypt | May-11 | 0.2 | 64.9 | 69.2 | 19.8 | 11.561 | 5.93453182 | 11.7932 |
| Egypt | Jun-11 | 0.1 | 65.0 | 69.5 | 19.0 | 12.095 | 5.9371 | 11.7363 |
| Egypt | Jul-11 | 1.5 | 66.1 | 70.4 | 16.7 | 11.756 | 5.9452 | 10.5181 |
| Egypt | Aug-11 | 1.0 | 67.7 | 71.1 | 12.3 | 11.995 | 5.9491 | 8.3841 |
| Egypt | Sep-11 | 0.8 | 68.2 | 72.2 | 8.9 | 13.024 | 5.9508 | 8.2459 |
| Egypt | Oct-11 | 0.1 | 67.9 | 72.4 | 8.7 | 12.242 | 5.9557 | 7.1006 |
| Egypt | Nov-11 | -1.8 | 68.1 | 73.1 | 11.6 | 14.258 | 5.9715 | 9.1045 |
| Egypt | Dec-11 | 0.0 | 67.8 | 72.9 | 13.2 | 13.95 | 6.0039 | 9.4595 |
| Egypt | Jan-12 | -1.0 | 68.2 | 73.0 | 11.2 | 13.834 | 6.0226 | 8.4695 |
| Egypt | Feb-12 | 0.0 | 69.2 | 73.5 | 12.6 | 13.834 | 6.0226 | 9.2125 |
| Egypt | Mar-12 | -1.1 | 70.5 | 74.4 | 10.9 | 13.834 | 6.0223 | 8.9312 |
| Egypt | Apr-12 | 1.5 | 72.3 | 75.2 | 10.8 | 13.938 | 6.029 | 8.8278 |
| Egypt | May-12 | 1.3 | 71.9 | 75.0 | 10.8 | 14.388 | 6.028 | 8.3815 |
| Egypt | Jun-12 | 1.6 | 71.0 | 74.6 | 9.2 | 14.753 | 6.0333 | 7.3381 |
| Egypt | Jul-12 | 2.2 | 71.4 | 74.9 | 8.1 | 14.196 | 6.0465 | 6.3920 |
| Egypt | Aug-12 | 1.5 | 73.3 | 75.7 | 8.2 | 14.227 | 6.0664 | 6.4698 |
| Egypt | Sep-12 | 0.8 | 74.5 | 76.6 | 9.3 | 12.396 | 6.0794 | 6.0942 |
| Egypt | Oct-12 | 1.7 | 73.9 | 77.2 | 8.8 | 12.414 | 6.0854 | 6.6298 |
| Egypt | Nov-12 | 1.8 | 71.9 | 76.2 | 5.5 | 12.494 | 6.0898 | 4.2408 |
| Egypt | Dec-12 | 0.8 | 71.7 | 76.3 | 5.8 | 12.964 | 6.1474 | 4.6639 |
| Egypt | Jan-13 | 1.5 | 73.5 | 77.6 | 7.9 | 12.825 | 6.5456 | 6.3014 |
| Egypt | Feb-13 | 1.2 | 75.7 | 79.6 | 9.3 | 12.723 | 6.7138 | 8.2993 |
| Egypt | Mar-13 | 2.6 | 76.7 | 80.1 | 8.9 | 12.868 | 6.7712 | 7.6613 |
| Egypt | Apr-13 | -0.1 | 78.8 | 81.3 | 9.0 | 13.018 | 6.86 | 8.1117 |
| Egypt | May-13 | 1.9 | 78.3 | 81.1 | 9.0 | 14.008 | 6.9636 | 8.1333 |
| Egypt | Jun-13 | 0.7 | 80.0 | 81.8 | 12.7 | 14.211 | 6.9906 | 9.6515 |
| Egypt | Jul-13 | 0.0 | 81.3 | 82.5 | 13.9 | 12.513 | 7.0065 | 10.1469 |
| Egypt | Aug-13 | 1.1 | 82.7 | 83.1 | 12.9 | 11.613 | 7.0187 | 9.7754 |
| Egypt | Sep-13 | 1.2 | 84.2 | 84.4 | 13.0 | 10.949 | 6.9053 | 10.1828 |
| Egypt | Oct-13 | -1.1 | 85.2 | 85.3 | 15.3 | 11.003 | 6.8857 | 10.4922 |
| Egypt | Nov-13 | 1.9 | 85.6 | 86.1 | 19.1 | 10.662 | 6.8843 | 12.9921 |
| Egypt | Dec-13 | 0.4 | 84.3 | 85.2 | 17.5 | 11.003 | 6.8986 | 11.6645 |
| Egypt | Jan-14 | 1.9 | 87.2 | 86.5 | 18.6 | 10.373 | 6.943 | 11.4691 |
| Egypt | Feb-14 | 1.2 | 87.5 | 87.3 | 15.7 | 10.151 | 6.9562 | 9.6734 |
| Egypt | Mar-14 | 1.6 | 88.7 | 87.8 | 15.6 | 10.226 | 6.95830273 | 9.6130 |
| Egypt | Apr-14 | 1.8 | 89.3 | 88.5 | 13.4 | 10.423 | 6.9781 | 8.8561 |
| Egypt | May-14 | 1.0 | 87.5 | 87.8 | 11.7 | 10.423 | 7.0808 | 8.2614 |
| Egypt | Jun-14 | 0.5 | 89.0 | 88.6 | 11.3 | 10.636 | 7.1429 | 8.3130 |
| Egypt | Jul-14 | 1.5 | 91.4 | 91.7 | 12.4 | 11.912 | 7.1453 | 11.1515 |
| Egypt | Aug-14 | 2.0 | 92.3 | 92.7 | 11.6 | 11.683 | 7.145 | 11.5523 |
| Egypt | Sep-14 | 1.6 | 94.2 | 93.8 | 11.8 | 11.605 | 7.145 | 11.1374 |
| Egypt | Oct-14 | 0.0 | 95.0 | 95.4 | 11.5 | 11.579 | 7.1463 | 11.8406 |
| Egypt | Nov-14 | 0.6 | 91.7 | 93.9 | 7.1 | 11.573 | 7.1454 | 9.0592 |
| Egypt | Dec-14 | 2.0 | 91.4 | 93.9 | 8.4 | 11.652 | 7.145 | 10.2113 |
| Egypt | Jan-15 | 0.3 | 92.3 | 94.8 | 5.8 | 11.11 | 7.2593 | 9.5954 |
| Egypt | Feb-15 | 0.2 | 94.5 | 96.6 | 8.0 | 10.849 | 7.5995 | 10.6529 |
| Egypt | Mar-15 | 1.3 | 96.8 | 98.1 | 9.2 | 11.179 | 7.6023 | 11.7312 |
| Egypt | Apr-15 | -1.0 | 98.1 | 98.2 | 9.8 | 11.227 | 7.6038 | 10.9605 |
| Egypt | May-15 | 0.0 | 100.4 | 99.3 | 14.8 | 11.499 | 7.6058 | 13.0979 |
| Egypt | Jun-15 | -0.2 | 98.7 | 98.6 | 10.9 | 11.196 | 7.6056 | 11.2867 |
| Egypt | Jul-15 | 0.9 | 99.0 | 99.3 | 8.3 | 11.432 | 7.8006 | 8.2879 |
| Egypt | Aug-15 | 3.4 | 99.9 | 100.0 | 8.2 | 11.159 | 7.8076 | 7.8749 |
| Egypt | Sep-15 | 3.3 | 104.5 | 102.4 | 11.0 | 11.245 | 7.8076 | 9.1684 |
| Egypt | Oct-15 | 2.1 | 106.9 | 104.7 | 12.5 | 11.322 | 7.9158 | 9.7484 |
| Egypt | Nov-15 | 1.2 | 105.1 | 104.4 | 14.7 | 11.209 | 7.8794 | 11.1821 |
| Egypt | Dec-15 | 0.3 | 104.7 | 104.3 | 14.6 | 11.336 | 7.8078 | 11.0756 |
| Egypt | Jan-16 | 0.2 | 104.6 | 104.4 | 13.4 | 11.409 | 7.8077 | 10.1266 |
| Egypt | Feb-16 | 1.5 | 106.4 | 105.4 | 12.5 | 11.344 | 7.8073 | 9.1097 |
| Egypt | Mar-16 | 2.2 | 108.5 | 106.9 | 12.1 | 13.217 | 8.452 | 8.9704 |
| Egypt | Apr-16 | 0.3 | 110.6 | 108.2 | 12.7 | 13.35 | 8.857247 | 10.1833 |
| Egypt | May-16 | 1.5 | 114.8 | 111.5 | 14.3 | 12.858 | 8.8573 | 12.2860 |
| Egypt | Jun-16 | 2.4 | 116.1 | 112.4 | 17.6 | 13.96 | 8.85732857 | 13.9959 |
| Egypt | Jul-16 | 1.5 | 117.2 | 113.2 | 18.4 | 14.272 | 8.8574 | 13.9980 |
| Egypt | Aug-16 | 1.5 | 119.2 | 115.4 | 19.3 | 14.03 | 8.8581 | 15.4000 |
| Egypt | Sep-16 | 1.5 | 120.0 | 116.9 | 14.8 | 14.775 | 8.85726 | 14.1602 |
| Egypt | Oct-16 | 1.2 | 121.7 | 118.9 | 13.8 | 14.594 | 8.85814389 | 13.5626 |
| Egypt | Nov-16 | 1.7 | 127.7 | 124.6 | 21.5 | 18.186 | 15.81253 | 19.3487 |
| Egypt | Dec-16 | -0.8 | 134.4 | 128.5 | 28.3 | 19.036 | 18.4225 | 23.2023 |
| Egypt | Jan-17 | 0.2 | 143.6 | 133.7 | 37.2 | 18.938 | 18.5991 | 28.0651 |
| Egypt | Feb-17 | -0.9 | 149.5 | 137.3 | 40.5 | 19.094 | 16.90407 | 30.2657 |
| Egypt | Mar-17 | 0.2 | 153.9 | 139.9 | 41.8 | 19.375 | 17.6472995 | 30.8700 |
| Egypt | Apr-17 | 0.6 | 158.8 | 142.3 | 43.6 | 19.35 | 18.02582 | 31.5157 |
| Egypt | May-17 | 1.1 | 162.0 | 144.7 | 41.1 | 20.519 | 18.0310591 | 29.7758 |
| Egypt | Jun-17 | 1.0 | 162.9 | 145.9 | 40.3 | 20.375 | 18.0398765 | 29.8043 |
| Egypt | Jul-17 | 2.0 | 166.9 | 150.6 | 42.3 | 21.564 | 17.8615905 | 33.0389 |
| Egypt | Aug-17 | 2.3 | 168.7 | 152.3 | 41.6 | 19.379 | 17.7138 | 31.9757 |
| Egypt | Sep-17 | 1.3 | 169.4 | 153.8 | 41.2 | 18.46 | 17.6133737 | 31.5654 |
| Egypt | Oct-17 | -0.4 | 169.8 | 155.5 | 39.6 | 18.571 | 17.59748 | 30.7822 |
| Egypt | Nov-17 | -0.3 | 169.0 | 157.0 | 32.3 | 18.869 | 17.6138 | 26.0032 |
| Egypt | Dec-17 | 2.2 | 168.3 | 156.7 | 25.2 | 18.77 | 17.743133 | 21.9455 |
| Egypt | Jan-18 | 0.9 | 167.8 | 156.6 | 16.9 | 18.006 | 17.6529757 | 17.1279 |
| Egypt | Feb-18 | 3.3 | 169.2 | 157.0 | 13.2 | 17.989 | 17.6235583 | 14.3481 |
| Egypt | Mar-18 | 4.2 | 172.2 | 158.6 | 11.8 | 17.999 | 17.5763111 | 13.3667 |
| Egypt | Apr-18 | 1.9 | 176.3 | 161.0 | 11.1 | 17.651 | 17.63139 | 13.1413 |
| Egypt | May-18 | 2.7 | 175.9 | 161.3 | 8.6 | 19.158 | 17.7829 | 11.4720 |
| Egypt | Jun-18 | 1.8 | 179.4 | 166.9 | 10.1 | 19.468 | 17.8251682 | 14.3934 |
| Egypt | Jul-18 | 1.6 | 183.0 | 170.9 | 9.6 | 18.713 | 17.84004 | 13.4794 |
| Egypt | Aug-18 | 1.9 | 188.4 | 174.0 | 11.6 | 19.059 | 17.8340741 | 14.2482 |
| Egypt | Sep-18 | 1.8 | 197.3 | 178.4 | 16.5 | 19.62 | 17.8556076 | 15.9948 |
| Egypt | Oct-18 | 1.1 | 204.2 | 183.0 | 20.2 | 19.709 | 17.85922 | 17.6849 |
| Egypt | Nov-18 | 0.6 | 200.5 | 181.6 | 18.7 | 19.359 | 17.86144 | 15.6688 |
| Egypt | Dec-18 | 0.3 | 187.1 | 175.4 | 11.2 | 19.848 | 17.8648 | 11.9336 |
| Egypt | Jan-19 | -0.4 | 188.7 | 176.5 | 12.5 | 18.114 | 17.80474 | 12.7075 |
| Egypt | Feb-19 | -0.2 | 195.3 | 179.6 | 15.4 | 17.663 | 17.529705 | 14.3949 |
| Egypt | Mar-19 | -0.7 | 198.3 | 181.0 | 15.2 | 17.248 | 17.3338857 | 14.1236 |
| Egypt | Apr-19 | -0.3 | 199.3 | 181.9 | 13.0 | 17.375 | 17.2229105 | 12.9814 |
| Egypt | May-19 | 1.3 | 202.4 | 184.0 | 15.1 | 17.593 | 16.9720714 | 14.0732 |
| Egypt | Jun-19 | 1.9 | 198.0 | 182.5 | 10.3 | 17.724 | 16.6839824 | 9.3469 |
| Egypt | Jul-19 | 2.1 | 199.5 | 185.7 | 9.0 | 17.895 | 16.5553429 | 8.6600 |
| Egypt | Aug-19 | 1.8 | 201.4 | 186.9 | 6.9 | 16.443 | 16.5192389 | 7.4138 |
| Egypt | Sep-19 | 1.3 | 197.9 | 186.9 | 0.3 | 15.052 | 16.3482818 | 4.7646 |
| Egypt | Oct-19 | 2.0 | 194.3 | 188.7 | -4.8 | 15.905 | 16.1733727 | 3.1148 |
| Egypt | Nov-19 | 2.0 | 191.4 | 188.2 | -4.6 | 15.35 | 16.0734421 | 3.6344 |
| Egypt | Dec-19 | 0.9 | 190.4 | 187.9 | 1.8 | 15.065 | 16.0300087 | 7.1266 |
| Egypt | Jan-20 | -0.5 | 193.7 | 189.1 | 2.6 | 14.362 | 15.828995 | 7.1388 |
| Egypt | Feb-20 | -0.3 | 193.6 | 189.1 | -0.9 | 13.157 | 15.62848 | 5.2895 |
| Egypt | Mar-20 | 0.9 | 194.8 | 190.3 | -1.7 | 12.533 | 15.66497 | 5.1381 |
| Egypt | Apr-20 | -0.3 | 201.9 | 192.6 | 1.3 | 12.439 | 15.69869 | 5.8824 |
| Egypt | May-20 | 1.0 | 201.0 | 192.6 | -0.7 | 12.371 | 15.72356 | 4.6739 |
| Egypt | Jun-20 | 0.9 | 198.2 | 192.8 | 0.1 | 13.294 | 16.1018 | 5.6438 |
| Egypt | Jul-20 | 1.4 | 196.4 | 193.5 | -1.5 | 13.418 | 15.9431 | 4.2003 |
| Egypt | Aug-20 | 1.8 | 193.2 | 193.2 | -4.1 | 13.525 | 15.88034 | 3.3708 |
| Egypt | Sep-20 | 3.5 | 192.7 | 193.8 | -2.6 | 13.152 | 15.72332 | 3.6918 |
| Egypt | Oct-20 | 2.9 | 193.0 | 197.3 | -0.7 | 13.388 | 15.65602 | 4.5575 |
| Egypt | Nov-20 | 0.2 | 198.2 | 198.9 | 3.6 | 13.135 | 15.6162 | 5.6854 |
| Egypt | Dec-20 | 2.3 | 195.8 | 198.1 | 2.8 | 12.627 | 15.6446 | 5.4284 |
| Egypt | Jan-21 | 2.5 | 192.7 | 197.2 | -0.5 | 12.678 | 15.6529 | 4.2834 |
| Egypt | Feb-21 | 0.8 | 192.6 | 197.5 | -0.5 | 12.793 | 15.6155 | 4.4421 |
| Egypt | Mar-21 | 0.8 | 197.0 | 198.7 | 1.1 | 13.105 | 15.6632 | 4.4141 |
| Egypt | Apr-21 | 0.8 | 201.4 | 200.5 | -0.2 | 13.135 | 15.6393 | 4.1018 |
| Egypt | May-21 | 3.0 | 204.6 | 202.0 | 1.8 | 13.165 | 15.6162545 | 4.8806 |
| Egypt | Jun-21 | 0.8 | 205.0 | 202.3 | 3.4 | 13.165 | 15.6163 | 4.9274 |
| Egypt | Jul-21 | 2.1 | 205.9 | 204.1 | 4.8 | 13.127 | 15.6403625 | 5.4780 |
| Egypt | Aug-21 | 1.8 | 205.9 | 204.1 | 6.6 | 12.627 | 15.6499682 | 5.6418 |
| Egypt | Sep-21 | 1.4 | 213.2 | 206.4 | 10.6 | 12.316 | 15.6583045 | 6.5015 |
| Egypt | Oct-21 | 1.1 | 215.3 | 209.7 | 11.6 | 12.384 | 15.6601421 | 6.2848 |
| Egypt | Nov-21 | 2.3 | 214.3 | 209.9 | 8.1 | 12.405 | 15.6605864 | 5.5304 |
| Egypt | Dec-21 | 0.6 | 212.2 | 209.7 | 8.4 | 11.58 | 15.6615091 | 5.8556 |
| Egypt | Jan-22 | -0.8 | 216.7 | 211.5 | 12.4 | 11.295 | 15.66098 | 7.2515 |
| Egypt | Feb-22 | -1.0 | 226.6 | 214.9 | 17.6 | 11.325 | 15.660865 | 8.8101 |
| Egypt | Mar-22 | -1.7 | 235.8 | 219.7 | 19.7 | 11.49 | 16.6836913 | 10.5687 |
| Egypt | Apr-22 | 3.0 | 253.8 | 226.9 | 26.0 | 13.02 | 18.3578111 | 13.1671 |
| Egypt | May-22 | 0.3 | 255.3 | 229.2 | 24.8 | 14.664 | 18.3806222 | 13.4653 |
| Egypt | Jun-22 | 1.0 | 250.8 | 228.8 | 22.4 | 15.268 | 18.6756286 | 13.0994 |
| Egypt | Jul-22 | 0.8 | 252.0 | 231.9 | 22.4 | 15.779 | 18.8599063 | 13.6208 |
| Egypt | Aug-22 | 1.9 | 253.6 | 233.8 | 23.1 | 16.065 | 19.0982826 | 14.5517 |
| Egypt | Sep-22 | 2.2 | 259.4 | 237.6 | 21.7 | 15.891 | 19.3442143 | 15.1163 |
| Egypt | Oct-22 | 0.2 | 266.8 | 243.7 | 23.9 | 16.182 | 20.1971571 | 16.2136 |
| Egypt | Nov-22 | 0.4 | 278.5 | 249.2 | 30.0 | 18.031 | 24.3823136 | 18.7232 |
| Egypt | Dec-22 | 2.3 | 291.2 | 254.4 | 37.3 | 18.088 | 24.6238048 | 21.3162 |
| Egypt | Jan-23 | 1.9 | 320.5 | 266.3 | 47.9 | 20.671 | 28.713535 | 25.9102 |
| Egypt | Feb-23 | -0.6 | 366.6 | 283.6 | 61.8 | 20.29 | 30.43372 | 31.9684 |
| Egypt | Mar-23 | 3.8 | 384.3 | 291.3 | 63.0 | 20.968 | 30.8001864 | 32.5899 |
| Egypt | Apr-23 | 1.7 | 392.7 | 296.2 | 54.8 | 21.858 | 30.838 | 30.5421 |
| Egypt | May-23 | 1.1 | 408.6 | 304.2 | 60.0 | 22.901 | 30.8386136 | 32.7225 |
| Egypt | Jun-23 | 1.3 | 416.0 | 310.7 | 65.8 | 23.29 | 30.8423056 | 35.7955 |
| Egypt | Jul-23 | 2.3 | 424.2 | 316.4 | 68.3 | 23.511 | 30.84225 | 36.4381 |
| Egypt | Aug-23 | 2.6 | 434.5 | 321.4 | 71.4 | 24.771 | 30.8421826 | 37.4679 |
| Egypt | Sep-23 | 2.9 | 450.3 | 327.7 | 73.6 | 25.217 | 30.8423 | 37.9209 |
| Egypt | Oct-23 | 2.2 | 457.0 | 331.1 | 71.3 | 25.468 | 30.8416045 | 35.8638 |
| Egypt | Nov-23 | 2.7 | 458.1 | 335.4 | 64.5 | 25.602 | 30.8414 | 34.5907 |
| Egypt | Dec-23 | 3.1 | 467.5 | 340.0 | 60.5 | 25.625 | 30.8408667 | 33.6478 |
| Nigeria | Jan-01 | -0.5 | 18.3 | 20.0 | 12.7 | 14.5 | 110.005 | 17.6471 |
| Nigeria | Feb-01 | -0.8 | 18.8 | 20.4 | 13.9 | 14.5 | 110.205 | 18.6047 |
| Nigeria | Mar-01 | 0.5 | 19.4 | 20.5 | 16.7 | 14.5 | 110.155 | 17.8161 |
| Nigeria | Apr-01 | 1.1 | 21.5 | 21.8 | 27.1 | 17 | 113.2 | 23.1638 |
| Nigeria | May-01 | 1.2 | 22.6 | 22.5 | 30.8 | 17 | 113.07 | 22.9508 |
| Nigeria | Jun-01 | 0.3 | 23.0 | 22.1 | 31.4 | 17 | 111.98 | 15.7068 |
| Nigeria | Jul-01 | 0.5 | 23.5 | 22.6 | 33.8 | 18 | 111.35 | 18.9474 |
| Nigeria | Aug-01 | 0.3 | 23.8 | 23.0 | 34.5 | 18 | 111.2 | 18.5567 |
| Nigeria | Sep-01 | 0.2 | 24.4 | 23.4 | 39.7 | 19 | 110.11 | 18.7817 |
| Nigeria | Oct-01 | 0.5 | 24.1 | 23.6 | 37.6 | 20 | 110.11 | 19.1919 |
| Nigeria | Nov-01 | 0.8 | 23.2 | 22.9 | 32.7 | 20 | 111.49 | 17.4359 |
| Nigeria | Dec-01 | 1.3 | 23.0 | 22.8 | 29.3 | 20.5 | 111.9 | 16.3265 |
| Nigeria | Jan-02 | -1.0 | 23.6 | 23.7 | 29.2 | 21.1 | 113.5 | 18.5000 |
| Nigeria | Feb-02 | -0.3 | 24.1 | 24.1 | 28.5 | 22.5 | 114.3 | 18.1373 |
| Nigeria | Mar-02 | 1.0 | 24.4 | 24.1 | 26.0 | 24.5 | 115.54 | 17.5610 |
| Nigeria | Apr-02 | 1.5 | 24.8 | 24.6 | 15.7 | 22.5 | 115.63 | 12.8440 |
| Nigeria | May-02 | 1.5 | 25.3 | 24.8 | 11.9 | 21.9 | 116.04 | 10.2222 |
| Nigeria | Jun-02 | 1.2 | 25.6 | 24.8 | 11.1 | 20.72 | 118.045 | 12.2172 |
| Nigeria | Jul-02 | 1.3 | 27.1 | 26.1 | 15.5 | 17.5 | 124.137 | 15.4867 |
| Nigeria | Aug-02 | 1.1 | 26.5 | 25.9 | 11.7 | 17.25 | 125.0086 | 12.6087 |
| Nigeria | Sep-02 | 1.1 | 26.1 | 25.8 | 7.0 | 16.49 | 125.9653 | 10.2564 |
| Nigeria | Oct-02 | 0.3 | 24.5 | 24.9 | 1.8 | 15.25 | 126.055 | 5.5085 |
| Nigeria | Nov-02 | 0.9 | 24.9 | 25.7 | 7.6 | 14.88 | 126.329 | 12.2271 |
| Nigeria | Dec-02 | 0.7 | 25.2 | 25.6 | 9.6 | 13.8 | 126.388 | 12.2807 |
| Nigeria | Jan-03 | 0.8 | 25.6 | 26.2 | 8.7 | 14.6 | 126.5718 | 10.5485 |
| Nigeria | Feb-03 | 1.5 | 25.4 | 25.8 | 5.1 | 15 | 126.9844 | 7.0539 |
| Nigeria | Mar-03 | 0.2 | 24.6 | 25.5 | 0.7 | 15.2 | 130.352 | 5.8091 |
| Nigeria | Apr-03 | 1.2 | 25.8 | 26.7 | 3.7 | 15.13 | 126.983 | 8.5366 |
| Nigeria | May-03 | 0.8 | 26.2 | 26.9 | 3.5 | 15.44 | 127.168 | 8.4677 |
| Nigeria | Jun-03 | 0.2 | 27.2 | 28.3 | 6.4 | 15.88 | 127.401 | 14.1129 |
| Nigeria | Jul-03 | 0.8 | 27.3 | 29.5 | 0.7 | 15.99 | 127.323 | 13.0268 |
| Nigeria | Aug-03 | 0.7 | 26.8 | 29.1 | 0.8 | 13.49 | 127.605 | 12.3552 |
| Nigeria | Sep-03 | 0.8 | 27.7 | 30.5 | 6.4 | 14.5 | 128.174 | 18.2171 |
| Nigeria | Oct-03 | 1.4 | 27.8 | 30.8 | 13.3 | 13.46 | 129.276 | 23.6948 |
| Nigeria | Nov-03 | 1.4 | 28.1 | 31.2 | 12.7 | 14.3 | 136.107 | 21.4008 |
| Nigeria | Dec-03 | 0.3 | 29.2 | 31.7 | 15.7 | 14.5 | 136.723 | 23.8281 |
| Nigeria | Jan-04 | 0.8 | 28.7 | 32.1 | 12.0 | 14.3 | 135.5823 | 22.5191 |
| Nigeria | Feb-04 | 0.1 | 28.8 | 32.2 | 13.8 | 14.2 | 134.655 | 24.8062 |
| Nigeria | Mar-04 | -0.1 | 28.3 | 31.2 | 15.2 | 13.99 | 133.082 | 22.3529 |
| Nigeria | Apr-04 | 1.1 | 29.5 | 31.3 | 14.5 | 14.25 | 132.99 | 17.2285 |
| Nigeria | May-04 | 0.9 | 30.9 | 32.2 | 18.2 | 14.25 | 132.012 | 19.7026 |
| Nigeria | Jun-04 | 0.9 | 31.2 | 32.3 | 14.5 | 14.25 | 132.25 | 14.1343 |
| Nigeria | Jul-04 | 0.6 | 30.6 | 32.6 | 12.3 | 14.25 | 132.299 | 10.5085 |
| Nigeria | Aug-04 | 1.0 | 31.2 | 32.9 | 16.5 | 14.25 | 132.329 | 13.0584 |
| Nigeria | Sep-04 | 1.3 | 31.8 | 33.3 | 14.7 | 14.25 | 132.345 | 9.1803 |
| Nigeria | Oct-04 | 1.1 | 32.0 | 34.1 | 15.2 | 15.13 | 132.382 | 10.7143 |
| Nigeria | Nov-04 | 1.5 | 32.3 | 34.3 | 14.9 | 14.55 | 132.372 | 9.9359 |
| Nigeria | Dec-04 | 1.3 | 32.6 | 34.9 | 11.9 | 14.4 | 132.358 | 10.0946 |
| Nigeria | Jan-05 | 0.0 | 33.0 | 35.2 | 14.9 | 15.48 | 132.36 | 9.6573 |
| Nigeria | Feb-05 | 3.3 | 34.2 | 35.7 | 18.5 | 12.65 | 132.471 | 10.8696 |
| Nigeria | Mar-05 | 2.4 | 35.3 | 36.3 | 24.6 | 12.5 | 132.353 | 16.3462 |
| Nigeria | Apr-05 | 1.5 | 35.4 | 36.9 | 20.0 | 7.99 | 132.353 | 17.8914 |
| Nigeria | May-05 | 0.9 | 35.7 | 37.6 | 15.5 | 8.75 | 132.32 | 16.7702 |
| Nigeria | Jun-05 | 0.4 | 36.8 | 38.3 | 18.0 | 5.03 | 132.368 | 18.5759 |
| Nigeria | Jul-05 | 1.0 | 41.4 | 41.1 | 35.2 | 5.14 | 132.37 | 26.0736 |
| Nigeria | Aug-05 | 1.0 | 43.0 | 42.1 | 38.0 | 3 | 131.75 | 27.9635 |
| Nigeria | Sep-05 | 1.2 | 41.1 | 41.4 | 29.2 | 2.15 | 129.02 | 24.3243 |
| Nigeria | Oct-05 | 0.8 | 39.8 | 40.4 | 24.3 | 2.45 | 129.54 | 18.4751 |
| Nigeria | Nov-05 | 1.2 | 38.6 | 39.5 | 19.5 | 7.96 | 129.387 | 15.1603 |
| Nigeria | Dec-05 | 1.8 | 37.6 | 38.9 | 15.3 | 10.83 | 129 | 11.4613 |
| Nigeria | Jan-06 | 2.7 | 37.8 | 39.0 | 14.6 | 13.68 | 130.29 | 10.7955 |
| Nigeria | Feb-06 | 2.7 | 37.7 | 39.6 | 10.4 | 11.49 | 129.59 | 10.9244 |
| Nigeria | Mar-06 | 1.0 | 38.6 | 40.7 | 9.3 | 9.86 | 128.7 | 12.1212 |
| Nigeria | Apr-06 | 0.6 | 38.7 | 41.6 | 9.5 | 8.6 | 128.47 | 12.7371 |
| Nigeria | May-06 | 0.7 | 38.8 | 41.6 | 8.7 | 6.65 | 128.45 | 10.6383 |
| Nigeria | Jun-06 | 1.2 | 39.1 | 41.5 | 6.2 | 8.41 | 128.46 | 8.3551 |
| Nigeria | Jul-06 | 1.6 | 40.0 | 42.4 | -3.6 | 9.79 | 128.38 | 3.1630 |
| Nigeria | Aug-06 | 1.0 | 42.1 | 43.7 | -2.1 | 8.4 | 128.33 | 3.8005 |
| Nigeria | Sep-06 | 0.7 | 42.8 | 44.0 | 4.2 | 6.98 | 128.29 | 6.2802 |
| Nigeria | Oct-06 | 1.3 | 41.6 | 42.9 | 4.7 | 7.94 | 128.28 | 6.1881 |
| Nigeria | Nov-06 | 0.1 | 40.7 | 42.6 | 5.5 | 7 | 128.29 | 7.8481 |
| Nigeria | Dec-06 | -0.3 | 39.1 | 42.3 | 4.0 | 7.75 | 128.29 | 8.7404 |
| Nigeria | Jan-07 | -0.7 | 37.9 | 42.1 | 0.0 | 7.1 | 128.2772 | 7.9487 |
| Nigeria | Feb-07 | 1.2 | 39.0 | 42.4 | 3.4 | 6.9 | 128.2687 | 7.0707 |
| Nigeria | Mar-07 | 0.6 | 39.3 | 42.8 | 1.8 | 6.85 | 128.1513 | 5.1597 |
| Nigeria | Apr-07 | 1.8 | 39.7 | 43.3 | 2.4 | 7.23 | 127.9814 | 4.0865 |
| Nigeria | May-07 | 1.3 | 39.8 | 43.5 | 2.5 | 7.2 | 127.5596 | 4.5673 |
| Nigeria | Jun-07 | 0.9 | 40.4 | 44.2 | 3.5 | 6.59 | 127.109 | 6.5060 |
| Nigeria | Jul-07 | 1.3 | 40.5 | 44.4 | 1.5 | 5.98 | 127.1859 | 4.7170 |
| Nigeria | Aug-07 | 0.8 | 41.7 | 45.6 | -0.9 | 6.24 | 126.6753 | 4.3478 |
| Nigeria | Sep-07 | 0.9 | 42.6 | 45.8 | -0.4 | 7.1 | 125.8826 | 4.0909 |
| Nigeria | Oct-07 | 1.5 | 41.7 | 44.8 | 0.3 | 6.23 | 124.276 | 4.4289 |
| Nigeria | Nov-07 | 1.9 | 42.2 | 44.8 | 3.5 | 6.67 | 120.1206 | 5.1643 |
| Nigeria | Dec-07 | 1.4 | 42.5 | 45.0 | 8.6 | 7.75 | 118.2097 | 6.3830 |
| Nigeria | Jan-08 | -1.0 | 42.7 | 45.7 | 12.8 | 8.58 | 117.98 | 8.5511 |
| Nigeria | Feb-08 | -0.7 | 42.5 | 45.8 | 8.8 | 8.63 | 118.21 | 8.0189 |
| Nigeria | Mar-08 | 1.6 | 44.2 | 46.2 | 12.5 | 8.5 | 117.92 | 7.9439 |
| Nigeria | Apr-08 | 0.6 | 44.9 | 46.9 | 13.1 | 8.17 | 117.87 | 8.3141 |
| Nigeria | May-08 | 0.8 | 45.6 | 47.8 | 14.6 | 8.27 | 117.83 | 9.8851 |
| Nigeria | Jun-08 | 1.1 | 47.7 | 49.5 | 17.9 | 8.64 | 117.81 | 11.9910 |
| Nigeria | Jul-08 | 0.8 | 48.9 | 50.6 | 20.6 | 9.21 | 117.77 | 13.9640 |
| Nigeria | Aug-08 | 0.8 | 49.4 | 51.2 | 18.6 | 9.13 | 117.74 | 12.2807 |
| Nigeria | Sep-08 | 0.9 | 49.9 | 51.8 | 16.9 | 9.08 | 117.73 | 13.1004 |
| Nigeria | Oct-08 | 1.2 | 49.6 | 51.4 | 18.9 | 7.72 | 117.72 | 14.7321 |
| Nigeria | Nov-08 | 1.0 | 49.7 | 51.4 | 17.8 | 6.9 | 117.74 | 14.7321 |
| Nigeria | Dec-08 | 1.9 | 49.9 | 51.8 | 17.6 | 5.61 | 126.48 | 15.1111 |
| Nigeria | Jan-09 | 1.6 | 50.4 | 52.1 | 18.1 | 3.88 | 145.78 | 14.0044 |
| Nigeria | Feb-09 | 2.0 | 50.8 | 52.5 | 19.7 | 2 | 147.14 | 14.6288 |
| Nigeria | Mar-09 | 1.4 | 51.3 | 52.8 | 16.0 | 2.53 | 147.72 | 14.2857 |
| Nigeria | Apr-09 | 1.3 | 51.6 | 53.1 | 15.1 | 3.33 | 147.23 | 13.2196 |
| Nigeria | May-09 | 0.9 | 52.7 | 54.1 | 15.5 | 3.27 | 147.84 | 13.1799 |
| Nigeria | Jun-09 | 1.5 | 53.8 | 55.1 | 13.0 | 3.32 | 148.2 | 11.3131 |
| Nigeria | Jul-09 | 1.4 | 55.1 | 56.2 | 12.7 | 3.93 | 148.59 | 11.0672 |
| Nigeria | Aug-09 | 1.3 | 55.7 | 56.9 | 12.6 | 4.8 | 151.86 | 11.1328 |
| Nigeria | Sep-09 | 1.1 | 56.0 | 57.1 | 12.3 | 4.8 | 152.3 | 10.2317 |
| Nigeria | Oct-09 | 1.2 | 56.3 | 57.4 | 13.5 | 5.08 | 149.36 | 11.6732 |
| Nigeria | Nov-09 | 1.1 | 56.3 | 57.8 | 13.4 | 4.48 | 150.85 | 12.4514 |
| Nigeria | Dec-09 | 1.6 | 57.8 | 59.0 | 15.8 | 4 | 149.69 | 13.8996 |
| Nigeria | Jan-10 | 1.7 | 58.5 | 59.6 | 16.1 | 3.72 | 149.78 | 14.3954 |
| Nigeria | Feb-10 | 3.3 | 59.4 | 60.7 | 16.8 | 2.33 | 150.22 | 15.6190 |
| Nigeria | Mar-10 | 1.6 | 59.3 | 60.6 | 15.8 | 1.04 | 149.83 | 14.7727 |
| Nigeria | Apr-10 | 2.6 | 60.1 | 61.1 | 16.4 | 1.2 | 149.89 | 15.0659 |
| Nigeria | May-10 | 1.6 | 59.8 | 61.1 | 13.6 | 1.63 | 150.31 | 12.9390 |
| Nigeria | Jun-10 | 1.1 | 62.0 | 62.8 | 15.2 | 2.29 | 150.19 | 13.9746 |
| Nigeria | Jul-10 | 0.8 | 63.1 | 63.5 | 14.4 | 2.94 | 150.1 | 12.9893 |
| Nigeria | Aug-10 | 0.8 | 64.3 | 64.6 | 15.4 | 2.63 | 150.27 | 13.5325 |
| Nigeria | Sep-10 | 0.4 | 63.9 | 64.9 | 14.2 | 6.6 | 151.03 | 13.6602 |
| Nigeria | Oct-10 | 0.8 | 64.3 | 65.1 | 14.2 | 6.75 | 151.25 | 13.4146 |
| Nigeria | Nov-10 | 1.9 | 64.4 | 65.2 | 14.3 | 7.58 | 150.22 | 12.8028 |
| Nigeria | Dec-10 | 0.7 | 65.0 | 66.0 | 12.4 | 7.47 | 150.48 | 11.8644 |
| Nigeria | Jan-11 | -0.2 | 66.9 | 66.8 | 14.2 | 7.49 | 151.55 | 12.0805 |
| Nigeria | Feb-11 | 2.0 | 66.4 | 67.4 | 11.9 | 7.09 | 151.94 | 11.0379 |
| Nigeria | Mar-11 | 1.7 | 66.6 | 68.4 | 12.2 | 8.27 | 152.51 | 12.8713 |
| Nigeria | Apr-11 | 0.9 | 66.5 | 68.0 | 10.6 | 9.52 | 153.97 | 11.2930 |
| Nigeria | May-11 | 1.2 | 66.7 | 68.6 | 11.6 | 8.63 | 154.8 | 12.2750 |
| Nigeria | Jun-11 | 1.2 | 67.3 | 69.3 | 8.5 | 8.2 | 154.5 | 10.3503 |
| Nigeria | Jul-11 | 1.2 | 67.7 | 69.5 | 7.4 | 7.08 | 151.86 | 9.4488 |
| Nigeria | Aug-11 | 0.7 | 69.5 | 70.7 | 8.1 | 7.41 | 152.72 | 9.4427 |
| Nigeria | Sep-11 | 0.7 | 70.0 | 71.6 | 9.4 | 8.92 | 155.26 | 10.3236 |
| Nigeria | Oct-11 | 0.6 | 70.0 | 72.0 | 8.9 | 15 | 153.26 | 10.5991 |
| Nigeria | Nov-11 | 0.4 | 70.5 | 72.0 | 9.5 | 14.53 | 155.77 | 10.4294 |
| Nigeria | Dec-11 | -0.3 | 72.0 | 72.8 | 10.7 | 14.27 | 158.21 | 10.3030 |
| Nigeria | Jan-12 | 0.1 | 73.7 | 75.2 | 10.2 | 14.85 | 158.39 | 12.5749 |
| Nigeria | Feb-12 | 1.8 | 72.7 | 75.4 | 9.5 | 14.76 | 157.87 | 11.8694 |
| Nigeria | Mar-12 | 0.5 | 74.3 | 76.6 | 11.6 | 14.49 | 157.59 | 11.9883 |
| Nigeria | Apr-12 | 1.7 | 74.4 | 76.7 | 12.0 | 13.92 | 157.33 | 12.7941 |
| Nigeria | May-12 | 0.3 | 75.3 | 77.3 | 12.8 | 13.34 | 157.28 | 12.6822 |
| Nigeria | Jun-12 | 0.2 | 75.7 | 78.2 | 12.4 | 14.08 | 157.44 | 12.8427 |
| Nigeria | Jul-12 | 0.1 | 75.9 | 78.4 | 12.1 | 13.86 | 157.43 | 12.8058 |
| Nigeria | Aug-12 | 0.1 | 76.4 | 78.9 | 10.0 | 14.26 | 157.38 | 11.5983 |
| Nigeria | Sep-12 | 0.1 | 77.3 | 79.7 | 10.5 | 12.75 | 157.34 | 11.3128 |
| Nigeria | Oct-12 | 0.9 | 78.0 | 80.4 | 11.5 | 12.94 | 157.32 | 11.6667 |
| Nigeria | Nov-12 | 1.5 | 78.6 | 80.9 | 11.6 | 12.6 | 157.31 | 12.3611 |
| Nigeria | Dec-12 | 0.7 | 79.4 | 81.5 | 10.3 | 11.77 | 157.32 | 11.9505 |
| Nigeria | Jan-13 | 1.2 | 80.0 | 82.0 | 8.6 | 11.17 | 157.3 | 9.0426 |
| Nigeria | Feb-13 | 1.5 | 80.5 | 82.6 | 10.8 | 9.9 | 157.3 | 9.5491 |
| Nigeria | Mar-13 | 2.0 | 81.3 | 83.2 | 9.4 | 10.17 | 157.31 | 8.6162 |
| Nigeria | Apr-13 | 0.6 | 81.8 | 83.7 | 9.9 | 10.41 | 157.31 | 9.1265 |
| Nigeria | May-13 | 0.9 | 82.3 | 84.2 | 9.3 | 10.64 | 157.3 | 8.9263 |
| Nigeria | Jun-13 | 1.1 | 82.9 | 84.7 | 9.5 | 11.6 | 157.31 | 8.3120 |
| Nigeria | Jul-13 | 1.4 | 83.4 | 85.2 | 9.9 | 11.56 | 157.32 | 8.6735 |
| Nigeria | Aug-13 | 0.0 | 83.8 | 85.4 | 9.7 | 11.3 | 157.31 | 8.2383 |
| Nigeria | Sep-13 | 0.4 | 84.5 | 86.0 | 9.4 | 10.91 | 157.32 | 7.9046 |
| Nigeria | Oct-13 | 0.7 | 85.2 | 86.7 | 9.2 | 10.8 | 157.42 | 7.8358 |
| Nigeria | Nov-13 | 1.3 | 85.9 | 87.3 | 9.3 | 10.8 | 157.27 | 7.9110 |
| Nigeria | Dec-13 | 1.2 | 86.7 | 88.0 | 9.2 | 10.97 | 157.27 | 7.9755 |
| Nigeria | Jan-14 | 1.0 | 87.4 | 88.6 | 9.2 | 10.81 | 157.2916 | 8.0488 |
| Nigeria | Feb-14 | 0.8 | 87.9 | 89.0 | 9.2 | 11.82 | 157.3075 | 7.7482 |
| Nigeria | Mar-14 | 1.1 | 88.8 | 89.7 | 9.2 | 11.92 | 157.3008 | 7.8125 |
| Nigeria | Apr-14 | 1.2 | 89.5 | 90.2 | 9.4 | 11.71 | 157.2918 | 7.7658 |
| Nigeria | May-14 | 0.5 | 90.2 | 91.0 | 9.7 | 10.24 | 157.2873 | 8.0760 |
| Nigeria | Jun-14 | 1.5 | 91.0 | 91.7 | 9.7 | 9.98 | 157.2873 | 8.2645 |
| Nigeria | Jul-14 | 1.5 | 91.6 | 92.2 | 9.8 | 9.88 | 157.2873 | 8.2160 |
| Nigeria | Aug-14 | 0.4 | 92.1 | 92.7 | 9.9 | 9.95 | 157.2873 | 8.5480 |
| Nigeria | Sep-14 | 0.5 | 92.7 | 93.2 | 9.6 | 9.75 | 157.3006 | 8.3721 |
| Nigeria | Oct-14 | 1.1 | 93.2 | 93.7 | 9.3 | 9.83 | 157.3141 | 8.0738 |
| Nigeria | Nov-14 | 1.3 | 93.7 | 94.2 | 9.1 | 9.82 | 159.9961 | 7.9038 |
| Nigeria | Dec-14 | 1.1 | 94.6 | 95.0 | 9.1 | 10.8 | 169.68 | 7.9545 |
| Nigeria | Jan-15 | -0.2 | 95.4 | 95.8 | 9.2 | 11.2 | 169.68 | 8.1264 |
| Nigeria | Feb-15 | 1.8 | 96.1 | 96.4 | 9.3 | 10.88 | 169.68 | 8.3146 |
| Nigeria | Mar-15 | 1.3 | 97.1 | 97.3 | 9.3 | 10.77 | 197.0727 | 8.4727 |
| Nigeria | Apr-15 | 0.3 | 98.0 | 98.1 | 9.5 | 10.23 | 197 | 8.7583 |
| Nigeria | May-15 | 1.7 | 99.0 | 99.1 | 9.7 | 10.03 | 197 | 8.9011 |
| Nigeria | Jun-15 | 2.0 | 100.0 | 100.1 | 10.0 | 9.95 | 196.9159 | 9.1603 |
| Nigeria | Jul-15 | 1.7 | 100.8 | 100.8 | 10.0 | 10 | 196.9714 | 9.3275 |
| Nigeria | Aug-15 | 1.2 | 101.4 | 101.3 | 10.1 | 10 | 197 | 9.2772 |
| Nigeria | Sep-15 | 0.7 | 102.1 | 102.0 | 10.1 | 10.36 | 196.9975 | 9.4421 |
| Nigeria | Oct-15 | 1.5 | 102.6 | 102.4 | 10.1 | 9.11 | 196.9886 | 9.2850 |
| Nigeria | Nov-15 | 1.1 | 103.4 | 103.1 | 10.3 | 5.62 | 196.9914 | 9.4480 |
| Nigeria | Dec-15 | -1.0 | 104.6 | 104.1 | 10.5 | 4.57 | 196.9865 | 9.5789 |
| Nigeria | Jan-16 | 0.3 | 105.5 | 105.0 | 10.6 | 4.12 | 197 | 9.6033 |
| Nigeria | Feb-16 | 0.5 | 107.0 | 107.4 | 11.3 | 4.91 | 197 | 11.4108 |
| Nigeria | Mar-16 | 2.7 | 109.4 | 109.7 | 12.7 | 5.53 | 197 | 12.7441 |
| Nigeria | Apr-16 | 1.9 | 110.8 | 111.5 | 13.1 | 7.27 | 197 | 13.6595 |
| Nigeria | May-16 | 1.3 | 113.6 | 114.6 | 14.8 | 8.04 | 197 | 15.6408 |
| Nigeria | Jun-16 | 0.8 | 115.2 | 116.5 | 15.2 | 8.32 | 231.7614 | 16.3836 |
| Nigeria | Jul-16 | 1.0 | 116.6 | 118.0 | 15.7 | 12.34 | 294.5722 | 17.0635 |
| Nigeria | Aug-16 | 1.0 | 118.0 | 119.2 | 16.3 | 15.25 | 309.7304 | 17.6703 |
| Nigeria | Sep-16 | 1.3 | 118.9 | 120.2 | 16.5 | 14 | 305.23 | 17.8431 |
| Nigeria | Oct-16 | 1.6 | 119.9 | 121.2 | 16.9 | 13.96 | 305.21 | 18.3594 |
| Nigeria | Nov-16 | 2.4 | 121.0 | 122.1 | 17.0 | 13.99 | 305.18 | 18.4287 |
| Nigeria | Dec-16 | 1.6 | 122.6 | 123.4 | 17.2 | 13.96 | 305.22 | 18.5399 |
| Nigeria | Jan-17 | 1.8 | 124.2 | 124.6 | 17.7 | 13.94870411 | 305.202381 | 18.6667 |
| Nigeria | Feb-17 | 0.3 | 126.6 | 126.5 | 18.4 | 13.75344685 | 305.3125 | 17.7840 |
| Nigeria | Mar-17 | 1.1 | 129.4 | 128.7 | 18.3 | 13.59893941 | 306.402174 | 17.3200 |
| Nigeria | Apr-17 | 1.4 | 132.0 | 130.7 | 19.2 | 13.58 | 306.052778 | 17.2197 |
| Nigeria | May-17 | 1.2 | 135.4 | 133.2 | 19.1 | 13.50341967 | 305.538095 | 16.2304 |
| Nigeria | Jun-17 | 0.9 | 138.1 | 135.3 | 19.8 | 13.49995811 | 305.715 | 16.1373 |
| Nigeria | Jul-17 | 0.9 | 140.2 | 137.0 | 20.2 | 13.35 | 305.861905 | 16.1017 |
| Nigeria | Aug-17 | 0.8 | 141.8 | 138.3 | 20.2 | 13.20015719 | 305.667391 | 16.0235 |
| Nigeria | Sep-17 | 1.1 | 143.0 | 139.4 | 20.2 | 13.18017471 | 305.886842 | 15.9734 |
| Nigeria | Oct-17 | 1.5 | 144.2 | 140.4 | 20.2 | 13.18 | 305.62381 | 15.8416 |
| Nigeria | Nov-17 | 1.3 | 145.5 | 141.5 | 20.2 | 13.05 | 305.904545 | 15.8886 |
| Nigeria | Dec-17 | 1.5 | 146.3 | 142.4 | 19.4 | . | 306.313889 | 15.3971 |
| Nigeria | Jan-18 | -0.3 | 147.6 | 143.5 | 18.9 | 12.27 | 305.777273 | 15.1685 |
| Nigeria | Feb-18 | 2.2 | 148.9 | 144.6 | 17.6 | 11.88 | 305.895 | 14.3083 |
| Nigeria | Mar-18 | 1.3 | 150.2 | 145.8 | 16.1 | 11.84 | 305.742857 | 13.2867 |
| Nigeria | Apr-18 | 1.2 | 151.6 | 147.1 | 14.8 | 11.43 | 305.61 | 12.5478 |
| Nigeria | May-18 | 1.2 | 153.6 | 148.7 | 13.4 | 10 | 305.82619 | 11.6366 |
| Nigeria | Jun-18 | 0.7 | 156.0 | 150.5 | 13.0 | 10.11 | 305.871053 | 11.2343 |
| Nigeria | Jul-18 | 0.8 | 158.1 | 152.2 | 12.8 | 10 | 305.814286 | 11.0949 |
| Nigeria | Aug-18 | 0.8 | 160.4 | 153.8 | 13.1 | 10.63745316 | 306.057143 | 11.2075 |
| Nigeria | Sep-18 | 0.7 | 162.0 | 155.1 | 13.3 | 11.00000132 | 306.2725 | 11.2626 |
| Nigeria | Oct-18 | 1.0 | 163.3 | 156.2 | 13.2 | 10.94054078 | 306.505 | 11.2536 |
| Nigeria | Nov-18 | 1.6 | 164.8 | 157.5 | 13.3 | 10.91 | 306.711905 | 11.3074 |
| Nigeria | Dec-18 | 0.4 | 166.1 | 158.7 | 13.5 | - | 306.921053 | 11.4466 |
| Nigeria | Jan-19 | 1.0 | 167.5 | 159.8 | 13.5 | 10.98 | 306.845455 | 11.3589 |
| Nigeria | Feb-19 | 0.2 | 168.8 | 161.0 | 13.4 | 10.91 | 306.768182 | 11.3416 |
| Nigeria | Mar-19 | 1.9 | 170.3 | 162.3 | 13.4 | 10.42 | 306.92381 | 11.3169 |
| Nigeria | Apr-19 | 1.8 | 172.2 | 163.8 | 13.6 | 10.24 | 306.9625 | 11.3528 |
| Nigeria | May-19 | 1.6 | 174.7 | 165.6 | 13.7 | 10 | 306.95 | 11.3652 |
| Nigeria | Jun-19 | 1.1 | 177.0 | 167.4 | 13.5 | 9.93 | 306.947059 | 11.2292 |
| Nigeria | Jul-19 | 1.1 | 179.2 | 169.1 | 13.3 | 9.92 | 306.936957 | 11.1038 |
| Nigeria | Aug-19 | 0.9 | 181.4 | 170.7 | 13.1 | 10.89 | 306.9325 | 10.9883 |
| Nigeria | Sep-19 | 1.2 | 183.7 | 172.5 | 13.4 | 11.1 | 306.919048 | 11.2186 |
| Nigeria | Oct-19 | 0.3 | 186.2 | 174.4 | 14.0 | 10.03 | 306.963636 | 11.6517 |
| Nigeria | Nov-19 | 2.3 | 188.5 | 176.2 | 14.4 | 6.73 | 306.952273 | 11.8730 |
| Nigeria | Dec-19 | 0.8 | 190.3 | 177.7 | 14.6 | 4.47 | 306.95 | 11.9723 |
| Nigeria | Jan-20 | 0.3 | 192.2 | 179.2 | 14.8 | 3.45 | 306.959091 | 12.1402 |
| Nigeria | Feb-20 | 0.0 | 193.9 | 180.6 | 14.8 | 3 | 306.955 | 12.1739 |
| Nigeria | Mar-20 | 1.5 | 195.7 | 182.1 | 14.9 | 2.39 | 326.625 | 12.1996 |
| Nigeria | Apr-20 | 2.0 | 198.0 | 184.0 | 14.9 | 1.91 | 361 | 12.3321 |
| Nigeria | May-20 | 1.8 | 200.8 | 186.2 | 15.0 | 2.45 | 361 | 12.4396 |
| Nigeria | Jun-20 | 1.5 | 203.7 | 188.4 | 15.1 | 1.9 | 361 | 12.5448 |
| Nigeria | Jul-20 | 1.0 | 206.8 | 190.8 | 15.4 | 1.3 | 377.190476 | 12.8326 |
| Nigeria | Aug-20 | 0.8 | 210.3 | 193.3 | 15.9 | 1.17 | 381 | 13.2396 |
| Nigeria | Sep-20 | 0.4 | 214.2 | 196.2 | 16.6 | 1.1 | 381 | 13.7391 |
| Nigeria | Oct-20 | 1.0 | 218.4 | 199.2 | 17.3 | 0.86 | 381 | 14.2202 |
| Nigeria | Nov-20 | 1.5 | 222.8 | 202.4 | 18.2 | 0.03 | 381 | 14.8695 |
| Nigeria | Dec-20 | 2.5 | 227.3 | 205.7 | 19.5 | 0.03 | 381 | 15.7569 |
| Nigeria | Jan-21 | 2.6 | 231.5 | 208.7 | 20.4 | 0.52 | 381 | 16.4621 |
| Nigeria | Feb-21 | 0.2 | 235.8 | 211.9 | 21.7 | 1.49 | 381 | 17.3311 |
| Nigeria | Mar-21 | 1.4 | 240.3 | 215.2 | 22.8 | 2 | 381 | 18.1768 |
| Nigeria | Apr-21 | 1.2 | 242.7 | 217.3 | 22.6 | 2 | 381 | 18.0978 |
| Nigeria | May-21 | 1.4 | 245.3 | 219.5 | 22.2 | 2.5 | 410 | 17.8840 |
| Nigeria | Jun-21 | 1.5 | 248.0 | 221.9 | 21.7 | 2.5 | 410.15 | 17.7813 |
| Nigeria | Jul-21 | 1.2 | 250.1 | 223.9 | 20.9 | 2.5 | 410.1 | 17.3480 |
| Nigeria | Aug-21 | 1.0 | 252.8 | 226.2 | 20.2 | 2.5 | 411 | 17.0202 |
| Nigeria | Sep-21 | 1.3 | 255.9 | 228.8 | 19.5 | 2.5 | 411 | 16.6157 |
| Nigeria | Oct-21 | 1.7 | 258.3 | 231.0 | 18.3 | 2.5 | 411.5 | 15.9639 |
| Nigeria | Nov-21 | 2.4 | 261.0 | 233.5 | 17.2 | 2.5 | 411.738636 | 15.3656 |
| Nigeria | Dec-21 | 1.4 | 266.7 | 237.8 | 17.3 | 2.49 | 414.335714 | 15.6053 |
| Nigeria | Jan-22 | 0.2 | 271.0 | 241.3 | 17.1 | 2.49 | 415.96 | 15.6205 |
| Nigeria | Feb-22 | -0.1 | 276.1 | 245.2 | 17.1 | 2.3 | 416.95 | 15.7150 |
| Nigeria | Mar-22 | 1.5 | 281.6 | 249.5 | 17.2 | 1.75 | 415.721739 | 15.9387 |
| Nigeria | Apr-22 | 1.2 | 287.2 | 253.9 | 18.3 | 1.74 | 415.526316 | 16.8431 |
| Nigeria | May-22 | 1.3 | 292.9 | 258.4 | 19.4 | 2.47 | 415.95 | 17.7221 |
| Nigeria | Jun-22 | 0.9 | 298.9 | 263.1 | 20.5 | 2.41 | 415.614286 | 18.5669 |
| Nigeria | Jul-22 | 1.0 | 305.0 | 267.9 | 21.9 | 2.76 | 417.260526 | 19.6516 |
| Nigeria | Aug-22 | 0.5 | 311.0 | 272.6 | 23.0 | 3.83 | 426.06087 | 20.5128 |
| Nigeria | Sep-22 | 0.3 | 315.5 | 276.3 | 23.3 | 5.68 | 435.570455 | 20.7605 |
| Nigeria | Oct-22 | 0.6 | 319.3 | 279.8 | 23.6 | 6.49 | 440.846842 | 21.1255 |
| Nigeria | Nov-22 | 0.9 | 323.7 | 283.7 | 24.0 | 6.5 | 445.581364 | 21.4989 |
| Nigeria | Dec-22 | 0.8 | 329.8 | 288.5 | 23.7 | 4.35 | 450.7075 | 21.3204 |
| Nigeria | Jan-23 | 0.9 | 336.6 | 293.9 | 24.2 | 1.39 | 460.992857 | 21.7986 |
| Nigeria | Feb-23 | 0.8 | 343.0 | 298.9 | 24.2 | 2.09 | 460.835 | 21.9005 |
| Nigeria | Mar-23 | 1.4 | 350.0 | 304.5 | 24.3 | 3.81 | 460.973478 | 22.0441 |
| Nigeria | Apr-23 | 1.3 | 357.4 | 310.3 | 24.5 | 5.73 | 460.95625 | 22.2135 |
| Nigeria | May-23 | 1.8 | 365.2 | 316.3 | 24.7 | 2.98 | 462.01 | 22.4071 |
| Nigeria | Jun-23 | 0.8 | 373.9 | 323.1 | 25.1 | 3.87 | 610.71 | 22.8050 |
| Nigeria | Jul-23 | 1.8 | 386.8 | 332.4 | 26.8 | 4.45 | 770.316571 | 24.0761 |
| Nigeria | Aug-23 | 1.5 | 401.6 | 343.0 | 29.1 | 5.13 | 762.120087 | 25.8254 |
| Nigeria | Sep-23 | 1.5 | 411.4 | 350.2 | 30.4 | 5.29 | 762.01855 | 26.7463 |
| Nigeria | Oct-23 | 1.7 | 419.3 | 356.2 | 31.3 | 5.39 | ... | 27.3052 |
| Nigeria | Nov-23 | 2.7 | 429.4 | 363.7 | 32.6 | 7.73 | ... | 28.1988 |
| Nigeria | Dec-23 | 1.2 | 441.0 | 372.0 | 33.7 | 8.93 | ... | 28.9428 |
| South Africa | Jan-01 | 0.1 | 35.9 | 47.7 | 5.0 | 10.15 | 7.78307 | 6.9507 |
| South Africa | Feb-01 | 0.3 | 36.3 | 47.9 | 5.3 | 10.12 | 7.81555 | 7.8829 |
| South Africa | Mar-01 | 1.0 | 36.4 | 48.2 | 4.6 | 10.17 | 7.87869 | 7.3497 |
| South Africa | Apr-01 | 0.6 | 36.6 | 48.4 | 4.0 | 10.34 | 8.08213 | 6.3736 |
| South Africa | May-01 | 0.9 | 36.8 | 48.6 | 3.9 | 10.38 | 7.97279 | 6.3457 |
| South Africa | Jun-01 | 1.3 | 36.8 | 48.8 | 3.2 | 9.99 | 8.05355 | 6.0870 |
| South Africa | Jul-01 | 0.0 | 37.0 | 48.8 | 3.7 | 9.53 | 8.15002 | 5.1724 |
| South Africa | Aug-01 | 0.6 | 37.2 | 48.7 | 4.0 | 9.37 | 8.31388 | 4.7312 |
| South Africa | Sep-01 | -0.4 | 37.5 | 48.8 | 4.6 | 9.24 | 8.63656 | 4.2735 |
| South Africa | Oct-01 | 1.3 | 38.0 | 48.8 | 6.3 | 8.87 | 9.2751 | 4.0512 |
| South Africa | Nov-01 | 0.3 | 38.7 | 49.0 | 8.5 | 8.76 | 9.75383 | 4.2553 |
| South Africa | Dec-01 | 0.0 | 39.7 | 49.3 | 11.4 | 9.2 | 11.595 | 4.6709 |
| South Africa | Jan-02 | 0.0 | 40.3 | 50.1 | 12.3 | 9.44 | 11.58786 | 5.0314 |
| South Africa | Feb-02 | 0.5 | 40.7 | 50.7 | 12.1 | 9.43 | 11.4786 | 5.8455 |
| South Africa | Mar-02 | 1.2 | 41.5 | 51.3 | 13.8 | 9.92 | 11.51097 | 6.4315 |
| South Africa | Apr-02 | 1.7 | 42.3 | 52.1 | 15.5 | 10.6 | 11.05925 | 7.6446 |
| South Africa | May-02 | 0.6 | 42.6 | 52.4 | 15.6 | 11.16 | 10.17727 | 7.8189 |
| South Africa | Jun-02 | -0.2 | 43.3 | 52.8 | 17.8 | 11.44 | 10.12834 | 8.1967 |
| South Africa | Jul-02 | -0.3 | 43.9 | 53.7 | 18.8 | 11.37 | 10.13105 | 10.0410 |
| South Africa | Aug-02 | 1.1 | 44.7 | 54.0 | 20.3 | 11.43 | 10.5842 | 10.8830 |
| South Africa | Sep-02 | 0.8 | 45.6 | 54.4 | 21.5 | 12.11 | 10.59582 | 11.4754 |
| South Africa | Oct-02 | 1.1 | 46.3 | 55.2 | 21.8 | 12.61 | 10.3286 | 13.1148 |
| South Africa | Nov-02 | -0.2 | 46.7 | 55.9 | 20.5 | 12.17 | 9.9585 | 14.0816 |
| South Africa | Dec-02 | 0.9 | 46.8 | 56.0 | 18.0 | 12.27 | 8.9485 | 13.5903 |
| South Africa | Jan-03 | 1.0 | 47.4 | 56.4 | 17.4 | 12.34 | 8.69182 | 12.5749 |
| South Africa | Feb-03 | 1.9 | 46.9 | 56.4 | 15.3 | 12.63 | 8.292 | 11.2426 |
| South Africa | Mar-03 | 0.9 | 47.1 | 56.7 | 13.6 | 12.73 | 8.03775 | 10.5263 |
| South Africa | Apr-03 | 2.4 | 47.1 | 56.8 | 11.5 | 12.74 | 7.72417 | 9.0211 |
| South Africa | May-03 | 1.1 | 47.4 | 56.8 | 11.3 | 12.55 | 7.69211 | 8.3969 |
| South Africa | Jun-03 | 0.4 | 47.2 | 56.7 | 9.0 | 11.21 | 7.9025 | 7.3864 |
| South Africa | Jul-03 | 0.6 | 47.2 | 56.4 | 7.5 | 10.89 | 7.54783 | 5.0279 |
| South Africa | Aug-03 | -0.7 | 47.3 | 56.6 | 5.7 | 10.25 | 7.39667 | 4.8148 |
| South Africa | Sep-03 | 0.3 | 47.2 | 56.2 | 3.6 | 9.42 | 7.3105 | 3.3088 |
| South Africa | Oct-03 | 1.3 | 47.4 | 55.7 | 2.3 | 8.22 | 6.95237 | 0.9058 |
| South Africa | Nov-03 | 1.2 | 47.8 | 55.1 | 2.4 | 7.69 | 6.72868 | -1.4311 |
| South Africa | Dec-03 | 0.8 | 47.6 | 55.1 | 1.6 | 7.31 | 6.50059 | -1.6071 |
| South Africa | Jan-04 | 0.5 | 48.0 | 55.3 | 1.3 | 7.6 | 6.97525 | -1.9504 |
| South Africa | Feb-04 | 1.0 | 48.1 | 55.5 | 2.4 | 7.51 | 6.76675 | -1.5957 |
| South Africa | Mar-04 | 0.2 | 48.1 | 55.7 | 2.1 | 7.72 | 6.64075 | -1.7637 |
| South Africa | Apr-04 | 1.1 | 48.1 | 55.8 | 2.1 | 7.77 | 6.58779 | -1.7606 |
| South Africa | May-04 | 2.1 | 48.2 | 55.8 | 1.7 | 7.71 | 6.826 | -1.7606 |
| South Africa | Jun-04 | 1.0 | 47.9 | 55.8 | 1.4 | 7.83 | 6.4185 | -1.5873 |
| South Africa | Jul-04 | -0.4 | 47.7 | 56.0 | 0.9 | 7.89 | 6.12071 | -0.7092 |
| South Africa | Aug-04 | 1.7 | 47.6 | 55.8 | 0.6 | 7.44 | 6.45778 | -1.4134 |
| South Africa | Sep-04 | -0.2 | 47.6 | 55.9 | 0.8 | 7.12 | 6.56406 | -0.5338 |
| South Africa | Oct-04 | 1.0 | 47.9 | 56.0 | 1.0 | 7.28 | 6.36214 | 0.5386 |
| South Africa | Nov-04 | 2.8 | 48.3 | 56.3 | 1.2 | 7.25 | 6.08658 | 2.1779 |
| South Africa | Dec-04 | 1.6 | 48.2 | 56.3 | 1.3 | 7.27 | 5.71 | 2.1779 |
| South Africa | Jan-05 | 0.9 | 48.6 | 56.4 | 1.2 | 7.29 | 5.915 | 1.9892 |
| South Africa | Feb-05 | 1.4 | 48.6 | 56.5 | 1.1 | 7.23 | 6.04429 | 1.8018 |
| South Africa | Mar-05 | 0.3 | 48.5 | 56.8 | 0.9 | 7.2 | 5.965 | 1.9749 |
| South Africa | Apr-05 | 0.3 | 48.6 | 57.0 | 0.9 | 6.95 | 6.16206 | 2.1505 |
| South Africa | May-05 | 1.2 | 48.5 | 56.8 | 0.7 | 6.75 | 6.35133 | 1.7921 |
| South Africa | Jun-05 | 1.2 | 48.3 | 56.7 | 0.7 | 6.76 | 6.74712 | 1.6129 |
| South Africa | Jul-05 | 1.7 | 48.6 | 57.1 | 2.1 | 6.73 | 6.70533 | 1.9643 |
| South Africa | Aug-05 | 1.2 | 48.7 | 57.2 | 2.4 | 6.72 | 6.455 | 2.5090 |
| South Africa | Sep-05 | 1.8 | 48.9 | 57.3 | 2.7 | 6.74 | 6.35143 | 2.5045 |
| South Africa | Oct-05 | 1.2 | 49.0 | 57.4 | 2.2 | 6.79 | 6.5925 | 2.5000 |
| South Africa | Nov-05 | 0.8 | 49.3 | 57.3 | 1.9 | 6.89 | 6.67645 | 1.7762 |
| South Africa | Dec-05 | -0.1 | 49.9 | 57.4 | 3.4 | 6.82 | 6.34643 | 1.9538 |
| South Africa | Jan-06 | 0.8 | 50.3 | 57.6 | 3.6 | 6.74 | 6.09639 | 2.1277 |
| South Africa | Feb-06 | 0.9 | 50.4 | 57.7 | 3.8 | 6.68 | 6.10733 | 2.1239 |
| South Africa | Mar-06 | -0.7 | 50.7 | 57.9 | 4.4 | 6.53 | 6.22808 | 1.9366 |
| South Africa | Apr-06 | 0.5 | 50.9 | 58.0 | 4.7 | 6.65 | 6.06231 | 1.7544 |
| South Africa | May-06 | -1.4 | 51.1 | 58.2 | 5.3 | 6.8 | 6.31342 | 2.4648 |
| South Africa | Jun-06 | 0.8 | 51.4 | 58.7 | 6.4 | 7.11 | 6.99639 | 3.5273 |
| South Africa | Jul-06 | 1.5 | 51.6 | 59.1 | 6.1 | 7.28 | 7.07788 | 3.5026 |
| South Africa | Aug-06 | -0.1 | 51.9 | 59.5 | 6.5 | 7.69 | 6.96029 | 4.0210 |
| South Africa | Sep-06 | 0.8 | 52.4 | 59.6 | 7.2 | 7.75 | 7.455 | 4.0140 |
| South Africa | Oct-06 | 1.5 | 53.0 | 59.9 | 8.3 | 8.22 | 7.65975 | 4.3554 |
| South Africa | Nov-06 | 0.7 | 53.2 | 59.9 | 8.0 | 8.23 | 7.26147 | 4.5375 |
| South Africa | Dec-06 | 0.8 | 53.3 | 60.2 | 6.9 | 8.39 | 7.04028 | 4.8780 |
| South Africa | Jan-07 | 1.2 | 54.2 | 60.6 | 7.7 | 8.7 | 7.16694 | 5.2083 |
| South Africa | Feb-07 | 1.4 | 54.1 | 60.5 | 7.3 | 8.66 | 7.17472 | 4.8527 |
| South Africa | Mar-07 | 0.9 | 54.5 | 60.9 | 7.5 | 8.28 | 7.34905 | 5.1813 |
| South Africa | Apr-07 | 1.6 | 55.1 | 61.5 | 8.4 | 8.28 | 7.10267 | 6.0345 |
| South Africa | May-07 | 0.7 | 55.7 | 61.7 | 9.0 | 8.5 | 7.00222 | 6.0137 |
| South Africa | Jun-07 | 0.5 | 56.1 | 62.2 | 9.1 | 9.02 | 7.167 | 5.9625 |
| South Africa | Jul-07 | 0.3 | 56.7 | 62.7 | 9.8 | 9.03 | 6.9725 | 6.0914 |
| South Africa | Aug-07 | 0.6 | 57.5 | 63.1 | 10.8 | 9.13 | 7.23 | 6.0504 |
| South Africa | Sep-07 | 2.0 | 58.5 | 63.5 | 11.7 | 9.31 | 7.1175 | 6.5436 |
| South Africa | Oct-07 | 0.1 | 59.5 | 64.1 | 12.1 | 9.77 | 6.76091 | 7.0117 |
| South Africa | Nov-07 | -0.3 | 60.1 | 64.3 | 12.8 | 10.22 | 6.69472 | 7.3456 |
| South Africa | Dec-07 | 0.1 | 60.3 | 64.7 | 13.1 | 10.48 | 6.80615 | 7.4751 |
| South Africa | Jan-08 | 0.2 | 61.3 | 65.6 | 13.2 | 10.47 | 7.0131 | 8.2508 |
| South Africa | Feb-08 | 0.9 | 61.5 | 65.9 | 13.6 | 10.32 | 7.66325 | 8.9256 |
| South Africa | Mar-08 | 0.3 | 62.5 | 66.8 | 14.7 | 10.18 | 7.97056 | 9.6880 |
| South Africa | Apr-08 | 0.0 | 63.2 | 67.3 | 14.7 | 10.27 | 7.785 | 9.4309 |
| South Africa | May-08 | 1.9 | 64.4 | 67.8 | 15.5 | 10.6 | 7.621 | 9.8865 |
| South Africa | Jun-08 | 1.0 | 65.5 | 68.7 | 16.9 | 11.42 | 7.92 | 10.4502 |
| South Africa | Jul-08 | 0.9 | 66.7 | 69.8 | 17.6 | 11.38 | 7.62568 | 11.3238 |
| South Africa | Aug-08 | 1.5 | 67.4 | 70.2 | 17.3 | 11.35 | 7.6614 | 11.2520 |
| South Africa | Sep-08 | -0.5 | 68.4 | 70.6 | 16.9 | 11.15 | 8.05714 | 11.1811 |
| South Africa | Oct-08 | 1.2 | 69.0 | 70.9 | 16.0 | 10.93 | 9.7448 | 10.6084 |
| South Africa | Nov-08 | 1.0 | 69.4 | 70.9 | 15.6 | 10.85 | 10.0985 | 10.2644 |
| South Africa | Dec-08 | 1.5 | 69.7 | 70.8 | 15.6 | 10.77 | 9.97425 | 9.4281 |
| South Africa | Jan-09 | 0.5 | 71.1 | 71.2 | 16.0 | 10.66 | 9.8993 | 8.5366 |
| South Africa | Feb-09 | 0.4 | 71.3 | 71.8 | 15.9 | 9.22 | 10.0063 | 8.9530 |
| South Africa | Mar-09 | 0.4 | 71.7 | 72.9 | 14.7 | 8.62 | 10.471 | 9.1317 |
| South Africa | Apr-09 | 1.5 | 72.0 | 73.2 | 13.9 | 8.28 | 8.99633333 | 8.7667 |
| South Africa | May-09 | 0.8 | 72.3 | 73.5 | 12.3 | 7.68 | 8.37861111 | 8.4071 |
| South Africa | Jun-09 | 1.0 | 72.1 | 73.7 | 10.1 | 7.23 | 8.04625 | 7.2780 |
| South Africa | Jul-09 | -0.1 | 71.7 | 74.4 | 7.6 | 7.39 | 7.95129545 | 6.5903 |
| South Africa | Aug-09 | 0.9 | 71.9 | 74.7 | 6.6 | 7.16 | 7.935 | 6.4103 |
| South Africa | Sep-09 | 0.7 | 71.9 | 74.8 | 5.1 | 6.94 | 7.52119048 | 5.9490 |
| South Africa | Oct-09 | 1.0 | 72.4 | 74.8 | 5.0 | 6.95 | 7.48309524 | 5.5007 |
| South Africa | Nov-09 | 0.0 | 72.3 | 74.8 | 4.1 | 7.01 | 7.51714286 | 5.5007 |
| South Africa | Dec-09 | 0.6 | 72.1 | 75.1 | 3.5 | 7.07 | 7.47857143 | 6.0734 |
| South Africa | Jan-10 | 0.5 | 72.6 | 75.4 | 2.0 | 7.11 | 7.4555 | 5.8989 |
| South Africa | Feb-10 | 1.1 | 72.4 | 75.8 | 1.6 | 7.08 | 7.66525 | 5.5710 |
| South Africa | Mar-10 | 1.7 | 72.4 | 76.4 | 1.0 | 6.95 | 7.41119048 | 4.8011 |
| South Africa | Apr-10 | 1.1 | 72.6 | 76.4 | 0.8 | 6.59 | 7.35210526 | 4.3716 |
| South Africa | May-10 | 1.8 | 72.7 | 76.6 | 0.6 | 6.58 | 7.651 | 4.2177 |
| South Africa | Jun-10 | 0.6 | 72.4 | 76.6 | 0.4 | 6.54 | 7.63309524 | 3.9349 |
| South Africa | Jul-10 | 1.7 | 72.6 | 77.1 | 1.2 | 6.48 | 7.52857143 | 3.6290 |
| South Africa | Aug-10 | 1.5 | 73.0 | 77.1 | 1.6 | 6.42 | 7.2997619 | 3.2129 |
| South Africa | Sep-10 | 1.9 | 72.9 | 77.2 | 1.4 | 6.08 | 7.12625 | 3.2086 |
| South Africa | Oct-10 | 0.9 | 73.1 | 77.4 | 1.0 | 5.97 | 6.90921053 | 3.4759 |
| South Africa | Nov-10 | 0.8 | 73.1 | 77.5 | 1.2 | 5.65 | 6.98305238 | 3.6096 |
| South Africa | Dec-10 | 0.4 | 73.3 | 77.7 | 1.6 | 5.59 | 6.83967632 | 3.4621 |
| South Africa | Jan-11 | 0.3 | 75.0 | 78.1 | 3.4 | 5.54 | 6.9231225 | 3.5809 |
| South Africa | Feb-11 | 0.9 | 75.0 | 78.5 | 3.6 | 5.53 | 7.17304688 | 3.5620 |
| South Africa | Mar-11 | 1.7 | 75.9 | 79.4 | 4.8 | 5.5 | 6.9188381 | 3.9267 |
| South Africa | Apr-11 | 0.2 | 76.0 | 79.7 | 4.8 | 5.46 | 6.73722188 | 4.3194 |
| South Africa | May-11 | 0.8 | 77.2 | 80.1 | 6.1 | 5.45 | 6.86783611 | 4.5692 |
| South Africa | Jun-11 | 0.0 | 77.5 | 80.5 | 7.0 | 5.46 | 6.7972 | 5.0914 |
| South Africa | Jul-11 | -0.6 | 78.0 | 81.2 | 7.5 | 5.49 | 6.798615 | 5.3178 |
| South Africa | Aug-11 | 0.4 | 78.2 | 81.2 | 7.1 | 5.49 | 7.0684 | 5.3178 |
| South Africa | Sep-11 | 1.0 | 79.0 | 81.7 | 8.5 | 5.49 | 7.5702 | 5.8290 |
| South Africa | Oct-11 | 0.7 | 80.6 | 82.2 | 10.2 | 5.49 | 7.9690025 | 6.2016 |
| South Africa | Nov-11 | -0.3 | 81.1 | 82.2 | 10.8 | 5.49 | 8.13348158 | 6.0645 |
| South Africa | Dec-11 | 0.4 | 81.5 | 82.5 | 11.2 | 5.46 | 8.17662105 | 6.1776 |
| South Africa | Jan-12 | 1.6 | 82.6 | 82.9 | 10.2 | 5.47 | 8.006665 | 6.1460 |
| South Africa | Feb-12 | 0.9 | 82.4 | 83.3 | 9.8 | 5.5 | 7.65891 | 6.1146 |
| South Africa | Mar-12 | 1.1 | 82.5 | 84.3 | 8.7 | 5.54 | 7.61069286 | 6.1713 |
| South Africa | Apr-12 | 0.1 | 82.5 | 84.7 | 8.5 | 5.56 | 7.83351389 | 6.2735 |
| South Africa | May-12 | 1.4 | 82.5 | 84.7 | 6.9 | 5.56 | 8.1533 | 5.7428 |
| South Africa | Jun-12 | 0.5 | 82.2 | 85.1 | 6.1 | 5.58 | 8.38894762 | 5.7143 |
| South Africa | Jul-12 | 0.5 | 82.2 | 85.1 | 5.4 | 5.37 | 8.24854 | 4.8030 |
| South Africa | Aug-12 | 0.4 | 82.1 | 85.5 | 5.0 | 5.05 | 8.27611818 | 5.2956 |
| South Africa | Sep-12 | -0.3 | 83.7 | 86.2 | 5.8 | 4.94 | 8.27497222 | 5.5080 |
| South Africa | Oct-12 | 0.3 | 85.7 | 86.7 | 6.3 | 4.94 | 8.6290275 | 5.4745 |
| South Africa | Nov-12 | 0.9 | 86.8 | 87.0 | 7.1 | 4.93 | 8.7944675 | 5.8394 |
| South Africa | Dec-12 | 0.9 | 86.8 | 87.2 | 6.5 | 4.99 | 8.64446875 | 5.6970 |
| South Africa | Jan-13 | 0.8 | 87.5 | 87.5 | 5.9 | 5.06 | 8.79497381 | 5.5489 |
| South Africa | Feb-13 | 1.1 | 87.1 | 88.3 | 5.8 | 5.04 | 8.89311316 | 6.0024 |
| South Africa | Mar-13 | 1.1 | 87.0 | 89.4 | 5.4 | 5.05 | 9.19231389 | 6.0498 |
| South Africa | Apr-13 | 0.1 | 87.4 | 89.5 | 5.9 | 5.12 | 9.1110825 | 5.6671 |
| South Africa | May-13 | 1.1 | 87.4 | 89.4 | 5.9 | 5.03 | 9.36957857 | 5.5490 |
| South Africa | Jun-13 | 1.1 | 87.4 | 89.7 | 6.3 | 5.12 | 10.0253706 | 5.4054 |
| South Africa | Jul-13 | 1.2 | 87.7 | 90.6 | 6.7 | 5.12 | 9.9043325 | 6.4630 |
| South Africa | Aug-13 | 0.1 | 88.1 | 90.9 | 7.4 | 5.09 | 10.0935476 | 6.3158 |
| South Africa | Sep-13 | 0.0 | 88.8 | 91.3 | 6.2 | 5.06 | 9.97582895 | 5.9165 |
| South Africa | Oct-13 | 0.5 | 89.6 | 91.6 | 4.5 | 5.04 | 9.91449286 | 5.6517 |
| South Africa | Nov-13 | 0.9 | 90.3 | 91.7 | 4.0 | 5.07 | 10.2035737 | 5.4023 |
| South Africa | Dec-13 | 0.2 | 90.3 | 91.8 | 4.0 | 5.14 | 10.3824647 | 5.2752 |
| South Africa | Jan-14 | 1.5 | 91.9 | 92.5 | 4.9 | 5.22 | 10.8935775 | 5.7143 |
| South Africa | Feb-14 | 1.3 | 92.3 | 93.5 | 6.0 | 5.56 | 10.9810447 | 5.8890 |
| South Africa | Mar-14 | 0.2 | 93.7 | 94.7 | 7.8 | 5.73 | 10.7458278 | 5.9284 |
| South Africa | Apr-14 | 0.4 | 95.0 | 95.2 | 8.7 | 5.74 | 10.5501263 | 6.3687 |
| South Africa | May-14 | 1.5 | 95.8 | 95.5 | 9.6 | 5.74 | 10.4097861 | 6.8233 |
| South Africa | Jun-14 | 0.6 | 95.9 | 95.7 | 9.7 | 5.79 | 10.6711875 | 6.6890 |
| South Africa | Jul-14 | 0.3 | 95.8 | 96.6 | 9.2 | 6.03 | 10.7041 | 6.6225 |
| South Africa | Aug-14 | 1.2 | 96.5 | 96.8 | 9.5 | 6.01 | 10.6628857 | 6.4906 |
| South Africa | Sep-14 | 1.7 | 96.5 | 96.8 | 8.6 | 6 | 10.97778 | 6.0241 |
| South Africa | Oct-14 | 1.0 | 96.5 | 97.0 | 7.7 | 5.9 | 11.0701 | 5.8952 |
| South Africa | Nov-14 | -0.1 | 96.9 | 97.0 | 7.3 | 5.84 | 11.1049222 | 5.7797 |
| South Africa | Dec-14 | 0.6 | 96.6 | 96.8 | 7.0 | 6.04 | 11.4605289 | 5.4466 |
| South Africa | Jan-15 | 1.4 | 97.5 | 96.7 | 6.1 | 6 | 11.5728947 | 4.5405 |
| South Africa | Feb-15 | 0.3 | 97.9 | 97.4 | 6.1 | 5.88 | 11.5825278 | 4.1711 |
| South Africa | Mar-15 | 1.1 | 98.6 | 98.6 | 5.2 | 5.8 | 12.0681795 | 4.1183 |
| South Africa | Apr-15 | 1.0 | 99.2 | 99.5 | 4.4 | 5.8 | 12.0057842 | 4.5168 |
| South Africa | May-15 | 2.4 | 99.8 | 99.7 | 4.2 | 5.73 | 11.9683289 | 4.3979 |
| South Africa | Jun-15 | 0.5 | 99.8 | 100.2 | 4.1 | 5.76 | 12.3010524 | 4.7022 |
| South Africa | Jul-15 | 0.9 | 99.8 | 101.0 | 4.2 | 6.03 | 12.4601432 | 4.5549 |
| South Africa | Aug-15 | 2.4 | 100.5 | 101.1 | 4.2 | 6.16 | 12.92008 | 4.4421 |
| South Africa | Sep-15 | 1.4 | 100.6 | 101.1 | 4.3 | 6.24 | 13.6130625 | 4.4421 |
| South Africa | Oct-15 | 3.1 | 101.4 | 101.4 | 5.1 | 6.16 | 13.5095381 | 4.5361 |
| South Africa | Nov-15 | 0.5 | 102.2 | 101.6 | 5.5 | 6.33 | 14.1151816 | 4.7423 |
| South Africa | Dec-15 | 2.8 | 102.8 | 101.8 | 6.4 | 6.74 | 14.9903976 | 5.1653 |
| South Africa | Jan-16 | 2.0 | 104.5 | 102.6 | 7.2 | 6.86 | 16.3650306 | 6.1013 |
| South Africa | Feb-16 | 1.9 | 107.1 | 104.1 | 9.4 | 6.93 | 15.76568 | 6.8789 |
| South Africa | Mar-16 | 1.1 | 109.1 | 104.9 | 10.7 | 7.04 | 15.3868395 | 6.3895 |
| South Africa | Apr-16 | 1.9 | 111.4 | 106.0 | 12.3 | 7.18 | 14.622425 | 6.5327 |
| South Africa | May-16 | 1.3 | 111.7 | 106.1 | 12.0 | 7.16 | 15.3764475 | 6.4193 |
| South Africa | Jun-16 | 1.8 | 111.7 | 106.7 | 12.0 | 7.2 | 15.08105 | 6.4870 |
| South Africa | Jul-16 | 0.5 | 112.3 | 107.6 | 12.6 | 7.35 | 14.4095 | 6.5347 |
| South Africa | Aug-16 | 1.9 | 113.3 | 107.5 | 12.8 | 7.3 | 13.7607929 | 6.3304 |
| South Africa | Sep-16 | 1.0 | 113.5 | 107.6 | 12.7 | 7.29 | 14.01824 | 6.4293 |
| South Africa | Oct-16 | 1.2 | 114.3 | 108.2 | 12.8 | 7.35 | 13.9733235 | 6.7061 |
| South Africa | Nov-16 | 1.5 | 114.9 | 108.6 | 12.4 | 7.48 | 13.9174675 | 6.8898 |
| South Africa | Dec-16 | 2.1 | 115.9 | 109.0 | 12.7 | 7.61 | 13.8385342 | 7.0727 |
| South Africa | Jan-17 | 0.3 | 117.6 | 109.7 | 12.5 | 7.46 | 13.5434417 | 6.9201 |
| South Africa | Feb-17 | 0.9 | 118.4 | 110.9 | 10.5 | 7.26 | 13.2642289 | 6.5322 |
| South Africa | Mar-17 | 1.1 | 118.9 | 111.4 | 9.0 | 7.23 | 12.9392976 | 6.1964 |
| South Africa | Apr-17 | 1.6 | 118.8 | 111.5 | 6.6 | 7.41 | 13.5128353 | 5.1887 |
| South Africa | May-17 | 1.8 | 119.4 | 111.8 | 6.8 | 7.43 | 13.28518 | 5.3723 |
| South Africa | Jun-17 | 1.6 | 119.4 | 112.1 | 6.8 | 7.34 | 12.91721 | 5.0609 |
| South Africa | Jul-17 | 1.4 | 119.5 | 112.2 | 6.4 | 7.32 | 13.1338763 | 4.2751 |
| South Africa | Aug-17 | 0.6 | 119.2 | 112.4 | 5.2 | 7.17 | 13.230926 | 4.5581 |
| South Africa | Sep-17 | 2.1 | 119.2 | 112.9 | 5.1 | 7.1 | 13.1345118 | 4.9257 |
| South Africa | Oct-17 | -0.1 | 119.9 | 113.3 | 4.9 | 7.26 | 13.6756251 | 4.7135 |
| South Africa | Nov-17 | 0.2 | 120.4 | 113.3 | 4.8 | 7.45 | 14.0781818 | 4.3278 |
| South Africa | Dec-17 | 0.5 | 120.8 | 113.8 | 4.2 | 7.53 | 13.1703025 | 4.4037 |
| South Africa | Jan-18 | 1.3 | 122.2 | 114.4 | 3.9 | 7.32 | 12.2040815 | 4.2844 |
| South Africa | Feb-18 | 0.9 | 122.4 | 115.1 | 3.4 | 7.12 | 11.8219659 | 3.7872 |
| South Africa | Mar-18 | 0.6 | 122.4 | 115.6 | 2.9 | 7.06 | 11.8355661 | 3.7702 |
| South Africa | Apr-18 | 1.7 | 122.8 | 116.3 | 3.4 | 6.98 | 12.0840518 | 4.3049 |
| South Africa | May-18 | 1.5 | 122.7 | 116.7 | 2.8 | 7.01 | 12.5293814 | 4.3828 |
| South Africa | Jun-18 | 1.4 | 122.8 | 117.0 | 2.9 | 7.04 | 13.2854879 | 4.3711 |
| South Africa | Jul-18 | 1.1 | 123.1 | 117.9 | 3.0 | 7.07 | 13.414464 | 5.0802 |
| South Africa | Aug-18 | 0.3 | 123.0 | 117.8 | 3.1 | 7.12 | 14.0889719 | 4.8043 |
| South Africa | Sep-18 | 0.5 | 123.4 | 118.4 | 3.5 | 7.14 | 14.7797376 | 4.8716 |
| South Africa | Oct-18 | 1.7 | 123.5 | 119.0 | 3.0 | 7.2 | 14.4963042 | 5.0309 |
| South Africa | Nov-18 | 0.9 | 124.1 | 119.1 | 3.1 | 7.32 | 14.0865903 | 5.1192 |
| South Africa | Dec-18 | 1.9 | 124.1 | 119.0 | 2.7 | 7.5 | 14.1805151 | 4.5694 |
| South Africa | Jan-19 | 1.1 | 125.8 | 118.8 | 2.9 | 7.51 | 13.8614932 | 3.8462 |
| South Africa | Feb-19 | 1.3 | 125.8 | 119.8 | 2.8 | 7.25 | 13.7956152 | 4.0834 |
| South Africa | Mar-19 | 2.0 | 126.3 | 120.7 | 3.2 | 7.04 | 14.3830671 | 4.4118 |
| South Africa | Apr-19 | 1.5 | 126.6 | 121.4 | 3.0 | 7.11 | 14.1543618 | 4.3852 |
| South Africa | May-19 | 2.9 | 127.0 | 121.8 | 3.5 | 7.17 | 14.4369725 | 4.3702 |
| South Africa | Jun-19 | 2.0 | 127.4 | 122.2 | 3.8 | 7.11 | 14.5664726 | 4.4444 |
| South Africa | Jul-19 | 2.2 | 127.4 | 122.6 | 3.5 | 7.03 | 14.0465615 | 3.9864 |
| South Africa | Aug-19 | 2.2 | 128.1 | 122.9 | 4.2 | 6.99 | 15.1422921 | 4.3294 |
| South Africa | Sep-19 | 2.0 | 128.4 | 123.3 | 4.1 | 6.85 | 14.8485154 | 4.1385 |
| South Africa | Oct-19 | 2.0 | 128.3 | 123.3 | 3.8 | 6.81 | 14.9065286 | 3.6134 |
| South Africa | Nov-19 | 2.1 | 128.7 | 123.4 | 3.7 | 7 | 14.8035641 | 3.6104 |
| South Africa | Dec-19 | 0.6 | 129.0 | 123.7 | 3.9 | 7.13 | 14.4356804 | 3.9496 |
| South Africa | Jan-20 | 1.1 | 130.6 | 124.0 | 3.8 | 6.86 | 14.3971689 | 4.3771 |
| South Africa | Feb-20 | 1.2 | 131.2 | 125.2 | 4.2 | 6.33 | 15.0153081 | 4.5075 |
| South Africa | Mar-20 | 0.9 | 131.5 | 125.7 | 4.1 | 6.09 | 16.6111853 | 4.1425 |
| South Africa | Apr-20 | 0.9 | 131.9 | 124.9 | 4.2 | 5.1 | 18.5760009 | 2.8830 |
| South Africa | May-20 | 1.4 | 132.3 | 124.2 | 4.2 | 4.16 | 18.1425643 | 1.9704 |
| South Africa | Jun-20 | 1.2 | 132.6 | 124.8 | 4.1 | 4.1 | 17.1331745 | 2.1277 |
| South Africa | Jul-20 | 1.1 | 132.8 | 126.5 | 4.2 | 3.95 | 16.771376 | 3.1811 |
| South Africa | Aug-20 | -0.1 | 132.9 | 126.7 | 3.7 | 3.69 | 17.2308032 | 3.0919 |
| South Africa | Sep-20 | 0.6 | 133.0 | 126.8 | 3.6 | 3.45 | 16.7158391 | 2.8386 |
| South Africa | Oct-20 | 1.8 | 134.9 | 127.3 | 5.2 | 3.47 | 16.4613389 | 3.2441 |
| South Africa | Nov-20 | 1.0 | 135.8 | 127.3 | 5.5 | 3.59 | 15.5486658 | 3.1605 |
| South Africa | Dec-20 | 1.0 | 136.6 | 127.5 | 5.9 | 3.76 | 14.9058398 | 3.0719 |
| South Africa | Jan-21 | 0.7 | 137.5 | 128.0 | 5.3 | 3.81 | 15.1254581 | 3.2258 |
| South Africa | Feb-21 | 0.5 | 137.8 | 128.8 | 5.0 | 3.77 | 14.7521346 | 2.8754 |
| South Africa | Mar-21 | 0.5 | 138.9 | 129.6 | 5.7 | 3.82 | 14.9866843 | 3.1026 |
| South Africa | Apr-21 | 2.1 | 140.2 | 130.6 | 6.3 | 3.78 | 14.4078725 | 4.5637 |
| South Africa | May-21 | 1.0 | 141.3 | 130.7 | 6.7 | 3.61 | 14.0602086 | 5.2335 |
| South Africa | Jun-21 | 1.9 | 141.4 | 131.1 | 6.6 | 3.76 | 13.9167223 | 5.0481 |
| South Africa | Jul-21 | -0.4 | 141.7 | 132.5 | 6.7 | 3.88 | 14.5329404 | 4.7431 |
| South Africa | Aug-21 | 0.5 | 142.0 | 133.0 | 6.8 | 3.72 | 14.7889834 | 4.9724 |
| South Africa | Sep-21 | 1.4 | 142.0 | 133.3 | 6.7 | 3.83 | 14.5323279 | 5.1262 |
| South Africa | Oct-21 | 0.2 | 143.3 | 133.7 | 6.2 | 3.76 | 14.8587085 | 5.0275 |
| South Africa | Nov-21 | 0.3 | 143.4 | 134.2 | 5.6 | 3.81 | 15.5125582 | 5.4203 |
| South Africa | Dec-21 | 0.7 | 144.0 | 135.0 | 5.4 | 3.87 | 15.8695397 | 5.8824 |
| South Africa | Jan-22 | 0.9 | 145.3 | 135.3 | 5.7 | 3.87 | 15.4911708 | 5.7031 |
| South Africa | Feb-22 | 1.1 | 146.7 | 136.1 | 6.5 | 3.95 | 15.2133829 | 5.6677 |
| South Africa | Mar-22 | 0.5 | 147.6 | 137.6 | 6.2 | 4.22 | 14.9786031 | 6.1728 |
| South Africa | Apr-22 | -0.2 | 148.6 | 138.4 | 6.0 | 4.3 | 14.9538077 | 5.9724 |
| South Africa | May-22 | 1.0 | 151.9 | 139.4 | 7.5 | 4.47 | 15.9010573 | 6.6565 |
| South Africa | Jun-22 | 1.0 | 153.9 | 140.9 | 8.9 | 5.13 | 15.7712537 | 7.4752 |
| South Africa | Jul-22 | 1.6 | 155.7 | 143.1 | 9.9 | 5.4 | 16.842701 | 8.0000 |
| South Africa | Aug-22 | 0.8 | 158.4 | 143.6 | 11.6 | 5.74 | 16.6845191 | 7.9699 |
| South Africa | Sep-22 | 1.9 | 159.0 | 143.7 | 12.0 | 5.78 | 17.549802 | 7.8020 |
| South Africa | Oct-22 | 3.2 | 160.4 | 144.1 | 12.0 | 6.12 | 18.1226067 | 7.7786 |
| South Africa | Nov-22 | 0.7 | 161.3 | 144.6 | 12.4 | 6.19 | 17.4796144 | 7.7496 |
| South Africa | Dec-22 | 1.0 | 162.0 | 145.2 | 12.5 | 6.6 | 17.2817232 | 7.5556 |
| South Africa | Jan-23 | 1.3 | 165.0 | 145.0 | 13.6 | 6.47 | 17.0875326 | 7.1693 |
| South Africa | Feb-23 | 0.8 | 166.6 | 146.1 | 13.5 | 6.58 | 17.8856321 | 7.3475 |
| South Africa | Mar-23 | 0.8 | 168.3 | 147.6 | 14.0 | 7.1 | 18.2692782 | 7.2674 |
| South Africa | Apr-23 | 1.1 | 169.3 | 148.3 | 14.0 | 7.72 | 18.1764313 | 7.1532 |
| South Africa | May-23 | 1.0 | 169.9 | 148.6 | 11.8 | 8.05 | 19.0526998 | 6.5997 |
| South Africa | Jun-23 | 1.4 | 170.8 | 148.8 | 10.9 | 8.49 | 18.7560254 | 5.6068 |
| South Africa | Jul-23 | 0.6 | 171.1 | 150.0 | 9.9 | 8.57 | 18.1840995 | 4.8218 |
| South Africa | Aug-23 | 1.3 | 171.1 | 150.4 | 8.0 | 8.44 | 18.7538094 | 4.7354 |
| South Africa | Sep-23 | 0.6 | 172.1 | 151.5 | 8.2 | 8.33 | 18.9784291 | 5.4280 |
| South Africa | Oct-23 | 1.2 | 174.8 | 152.9 | 9.0 | 8.55 | 19.0508123 | 6.1069 |
| South Africa | Nov-23 | 2.3 | 176.1 | 152.7 | 9.2 | 8.52 | 18.537949 | 5.6017 |
| South Africa | Dec-23 | 0.9 | 176.0 | 152.7 | 8.6 | 8.45 | 18.6702314 | 5.1653 |
